# Ligand-Enabled Photocatalytic Reactivity of Iron(III) Halide Salts with Cyan and Green Light

**DOI:** 10.1021/acs.orglett.5c04928

**Published:** 2026-01-14

**Authors:** Mikayla M. Wymore, David B.C. Martin

**Affiliations:** Department of Chemistry, 4083 University of Iowa, Iowa City, Iowa 52242, United States

## Abstract

Iron­(III) halide salts have emerged as cheap and efficient catalysts for photocatalytic transformations, including C–H alkylation, C–H and alkene oxidation, azidation/amination, and others. The majority of these methods require UV light (λ ≤ 390 nm), and systematic efforts to enable the use of lower-energy visible light remain valuable. We report the use of simple Fe­(III) salts that enable C–H functionalizations with longer wavelength visible light up to 525 nm (green light). The one-pot conversion of aldehydes to amides, esters, and ketones as well as direct alkylation are described using blue, cyan, and green light.

## Graphical abstract

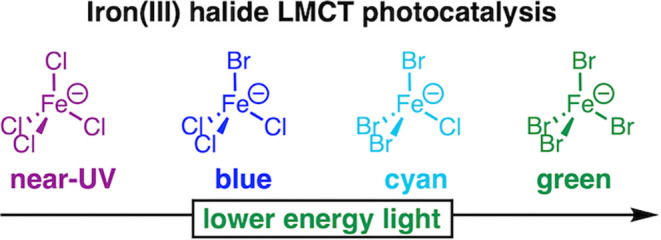

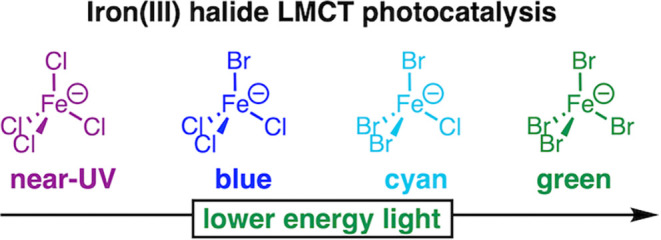

Photochemistry has been a powerful tool in organic synthesis since the original reports by Ciamician of the photochemical [2 + 2] cycloaddition of carvone.^1,2^ Numerous developments have focused on the discovery of new photoactive species and, in particular, facilitating photochemical reactions with longer wavelength visible light.^3,4^ The recent resurgence of photoredox catalysis can be attributed in part to the convenient use of simple visible light sources including fluorescent bulbs and blue LEDs; however, the absorption maxima of many widely used photocatalysts are in the near-ultraviolet (NUV) range and productive absorption beyond blue wavelengths is uncommon.^5–7^ The use of high-energy NUV and blue light in many photocatalytic systems leads to some disadvantages, such as reactive functional group incompatibility and rapid light attenuation at the surface of the reaction vessel according to the Beer–Lambert law.^8,9^ Recent work by the Rovis group has led to photoredox catalysts that are driven by red and near-infrared light, and reports from König, Gansäuer, Gianetti, and others have made advances in the use of visible light in the green to red (525–700 nm) region of the spectrum.^10–16^ Our group has reported strategies for shifting the reactivity of TiO_2_ to visible wavelengths of up to 525 nm.^17,18^ Despite these widespread efforts, new strategies to enable photocatalysis with low energy visible light remain valuable.

We recently became interested in the use of iron catalysts for visible light photocatalysis.^19^ Building on the early work of Shul’pin and Kats (Figure 1A),^20,21^ many recent reports have used iron trichloride as a source of chlorine radicals via ligand-to-metal charge transfer (LMCT, Figure 1B), including applications in C–C bond formations, C–X bond formation, selective oxidations and polymer degradation.^22–39^ A drawback of these methods is the requirement for NUV light with wavelengths ≤390 nm in the vast majority of cases. In 2018, Jiang and co-workers reported a new crystalline salt TBA­[FeCl_3_Br] (**1b**, TBA = tetra-*n*-butylammonium) that facilitated a benzylic oxidation reaction under visible light (blue LED, Figure 1C).^40^ While alternative mechanisms have been put forward involving photoredox processes,^40,41^ recent work by our group showed that catalyst **1b** exhibits wavelength-selective LMCT behavior, producing either chlorine or bromine radicals based on wavelength-dependent excitations.^19^ We wondered how far this ligand-dependent reactivity could be pushed given the ongoing importance of developing photocatalytic systems that respond to longer wavelengths. In 2017, Matyjaszewski and co-workers described the use of TBA­[FeBr_4_] (**1d**) for atom transfer radical polymerization driven by blue light, with a slower rate being observed with green light irradiation (520 nm).^42^ Herein, we report the development of simple iron halide-based photocatalysts including a new heteroleptic species TBA­[FeClBr_3_] (**1c**) that performs C–H functionalization driven by lower-energy light such as cyan and green light.

**Figure 1:**
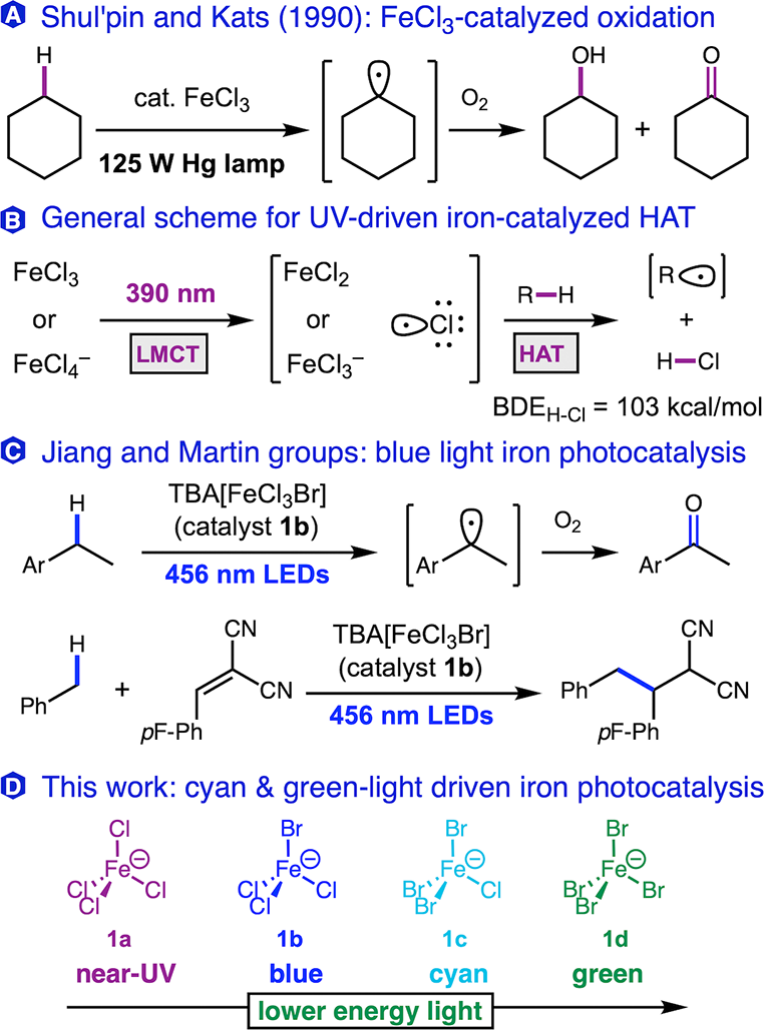
Examples of photocatalytic iron C–H functionalization with simple Fe­(III) halide salts.

We began our investigations by identifying a suitable model reaction for C–H functionalization mediated by halogen radicals. Radical H atom transfer (HAT) is an attractive proving ground due to the versatility of the resulting carbon-centered radicals,^43,44^ and light-driven HAT methods are particularly suited for this process.^45,46^ We focused on the conversion of aldehydes to acyl radicals that could be leveraged in several reactions including alkylation.^19,25^ Initial studies were conducted with the conversion to acyl halide intermediates, which can be readily converted to other carboxylic acid derivatives such as amides in a one-pot process (Table 1). Beginning with the conditions of our recent alkylation work, *N*-chlorosuccinimide (NCS) was effective for high conversion to the acid chloride intermediate after 20 h,^47–49^ which was then treated with benzylamine to give benzamide **4** in good yield by ^1^H NMR (entry 1, 74% yield). Catalyst **1d** was slightly less efficient with 456 nm light (entry 2, 61% yield). NBS was also tested and showed poor results under these conditions (see Table S1).^49,50^ A control reaction with NCS at 456 nm showed no background reaction, indicating that the reaction is promoted by the ferrate catalyst (entry 3, 0% yield, 94% remaining aldehyde). The iron catalysts **1** are nonhygroscopic crystalline solids and tolerate air, offering some advantages over methods with precious metals and peroxide reagents;^47^ however, other methods using NCS or NBS may have complementary advantages.^48,49^

**Table 1:**
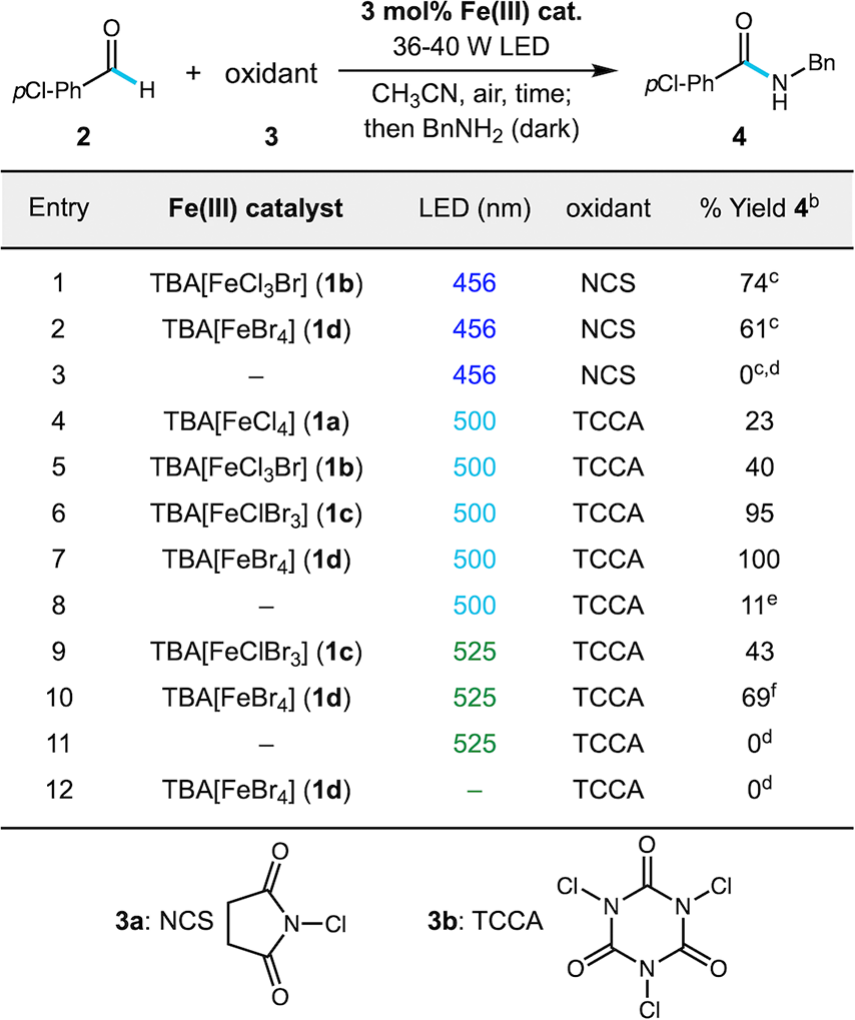
Optimization Studies of Aldehyde Amidation Using Simple Iron­(III) Halide Salts^a^

a Reactions performed on a 0.2 mmol scale with NCS (1.3 equiv) or TCCA (0.5 equiv), 0.2 M in CH_3_CN under air using one 40 W Kessil LED lamp or 36 W 500 nm LED lamp over 4 h with fan cooling. Stage 2 is performed by adding 3 equiv BnNH_2_ and stirring without irradiation for 2 h.

b Yields determined by ^1^H NMR using dibromomethane or mesitylene as an internal standard

c Reaction time with NCS is 20 h.

d ≥94% aldehyde remaining.

e 89% aldehyde remaining.

f 20% aldehyde remaining.

We also tested trichloroisocyanuric acid (TCCA, **3b**) as an oxidant due to its low cost and ability to contribute up to three chlorine atoms.^51–54^ Using 0.5 equiv of TCCA, up to quantitative conversion could be obtained, providing amide **4** in up to quantitative yield by ^1^H NMR in a catalyst-dependent manner with 500 nm cyan light (Table 1, entries 4–7). Catalysts **1c** and **1d** provided complete conversion in only 4 h, validating that additional bromide ligands red-shift LMCT reactivity to enable the use of low energy light. Moving even further to green light (525 nm), catalysts **1c** and **1d** provided substantial conversion in 4 h (entries 9–10, 43–69% yield), with some aldehyde remaining (e.g., 20% for Entry 10). Extending the reaction time to 7 h allowed for complete conversion and isolation of amide **4** in 59% yield (Scheme 1). Control reactions provided some important insight: TCCA gave some background reaction at 500 nm (entry 8, 11% yield after 4 h) that increased at shorter wavelengths, as reported by De Luca using blue LEDs and sunlight.^53^ This background reactivity was completely absent at 525 nm (entry 11, 0% yield, 95% remaining aldehyde). Control reactions without light led to complete recovery of starting materials (e.g., entry 12, 0% yield). We note that all reactions in Table 1 were carried out under air with no effort to exclude atmospheric oxygen, and in fact slower conversion was observed under N_2_ (see Table S1 and discussion below).

**Scheme 1:**
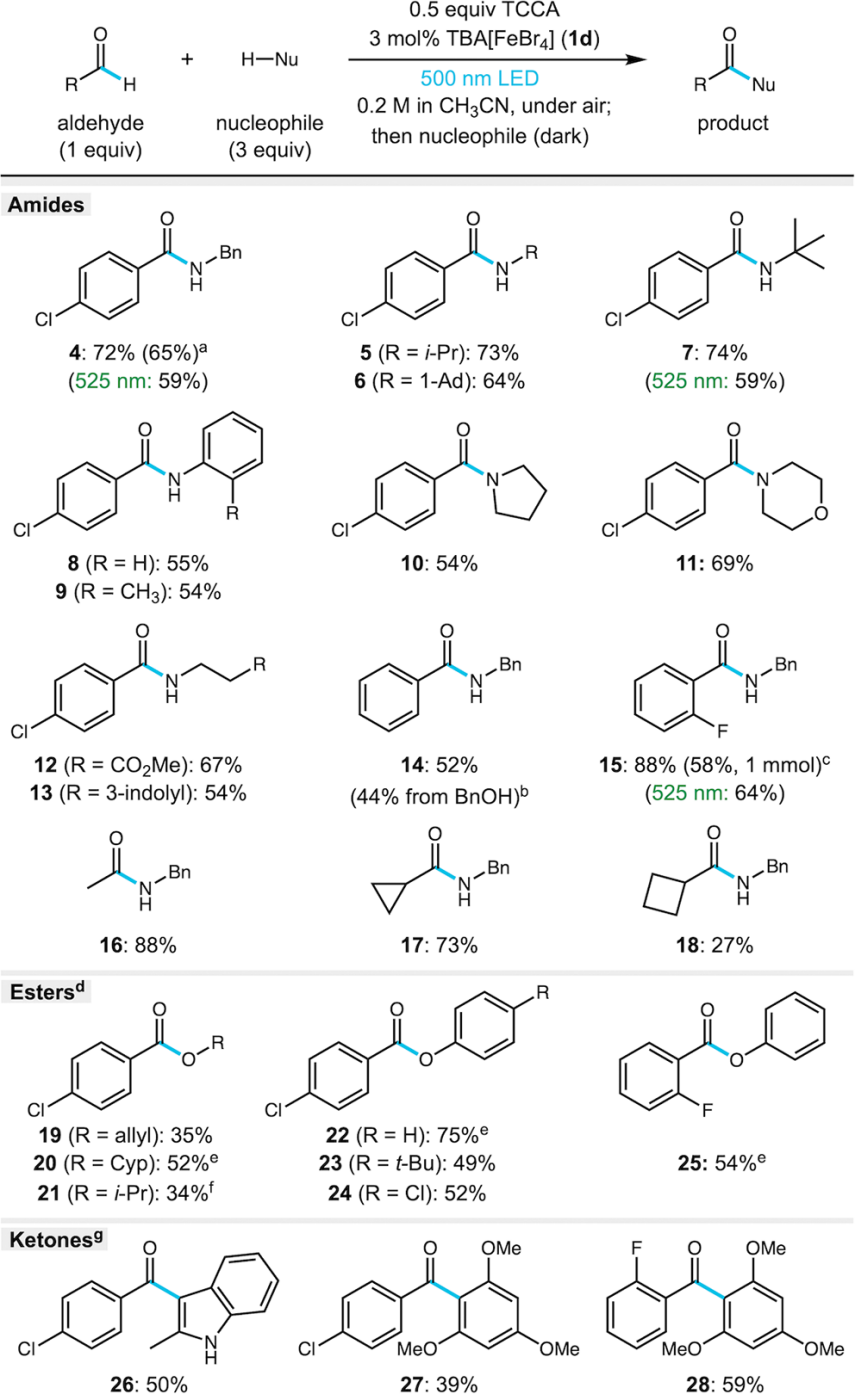
Substrate Scope of Iron-Catalyzed Aldehyde Functionalization^a^

a Using catalyst **1c** TBA­[FeClBr_3_].
b Using 1 equiv of TCCA, reaction time is 6 h for acid chloride formation.
c Reaction performed on a 1 mmol scale.
d Ester formation: 3 equiv of ROH, 3.75 equiv of NEt_2_ *i*-Pr, 0.5 equiv of DMAP, reaction time of 16 h.
e Esterification performed with 3.75 equiv of pyridine as the base.
f Esterification performed at 60 °C.
g Ketone formation: after the removal of CH_3_CN, the reaction is performed with 3 equiv of arene in HFIP with a reaction time of 16 h.
h Standard reaction time is 5 h for acid chloride formation, 7 h when using 525 nm light, followed by 2 h without irradiation for amide formation.

The significant improvement in reactivity of catalysts **1c** and **1d** can be understood by examination of the UV–vis absorption spectra (Figure 2). A significant absorption peak around 470 nm that extends to nearly 550 nm leads to much greater absorption compared to previous FeCl_3_-derived catalysts.^40^ We previously reported a DFT analysis of catalyst **1b** that assigned absorption above 400 nm to an LMCT state with electron transfer primarily from the Br ligand to the Fe center, leading to a bromine radical.^19^ The productive reactivity of catalysts **1c** and **1d** with cyan and green light can likely be attributed to lower-energy LMCT states that similarly lead to the bromine radical.

**Figure 2:**
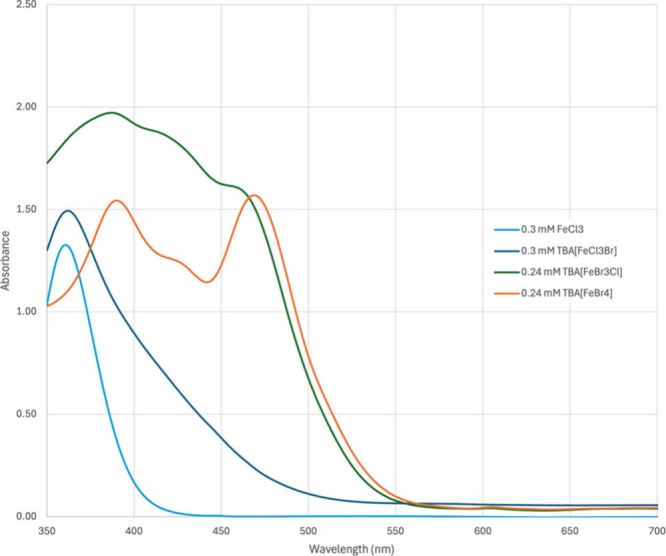
UV–vis absorption data for catalysts **1b**–**1d** and FeCl_3_, 0.24–0.30 mM in CH_3_CN, showing the significant redshift of more highly brominated ferrate species.

Having achieved excellent reactivity with low-energy visible light and low catalyst loading, we explored the scope of the one-pot aldehyde to amide conversion using TBA­[FeBr_4_] **1d** under visible light irradiation (≥500 nm, Scheme 1). Diverse amines are generally tolerated well in this reaction, including α-1°, α-2°, and α-3° amines (**4**–**7**, 64–74% yield). A reaction run with catalyst **1c** gave a comparable result (65% yield of **4**), while catalyst **1d** with green light led to slightly lower yields after a longer reaction time (59% yield of **4** and **7**). Aniline nucleophiles and secondary alkyl amines were also moderately efficient under cyan light irradiation (**8**–**11**, 54–69% yield). Not surprisingly, other functionalized primary amines such as β-alanine methyl ester and tryptamine were successful (**12** and **13**, 67% and 54% yield, respectively).

Other aldehydes were also explored for this reaction. Benzaldehyde was less efficient (**14**, 52% yield) but *o*-fluorobenzaldehyde was an excellent substrate, providing amide **15** in 88% yield. Electron-rich aldehydes were less successful, with *p*-OTIPS and *p*-OPiv substituted benzaldehydes providing poor results (<10%, see SI for more details). *p*-Anisaldehyde led to a mixture of products that included the functionalization of the OCH_3_ group. Aldehydes with strongly Lewis basic groups such as N-heterocycles were not very successful, likely due to coordination to iron that could disrupt the LMCT process. Selectivity for the aldehyde C–H bond was modest, as demonstrated by a mixture of products from tolualdehydes, *p*-anisaldehyde, and hexanal (see SI for more details). On the other hand, acetaldehyde showed excellent reactivity, providing the acetamide product **16** in 88% yield, indicating no competing side reactions for this substrate. Similarly, cyclopropylcarboxaldehyde was quite efficient (**17**, 73% yield) due to the inert nature of the ring C–H bonds, while cyclobutanecarboxaldehyde was less successful (**18**, 27% yield). Interestingly, benzyl alcohol could be oxidized up to benzoyl chloride under these conditions with cyan light and additional TCCA, providing product **14** in 44% yield in a one-pot process.^54,55^

To highlight the utility of the acyl chloride intermediate, we also explored the one-pot conversion to esters and ketones (Scheme 1, bottom). After completion of the photochemical process, addition of alcohol substrates and amine base gave moderate to good yields of esters (**19**–**25**, 34–75% yield), with pyridine providing the best results. The presence of iron salts and residual TCCA and byproducts likely has a detrimental effect on these reactions; however, 1° and 2° alcohols are successfully incorporated into the ester products. Phenol nucleophiles work in good yields (**22**–**25**, 49–75% yield). The direct conversion to ketones via *in situ* Friedel–Crafts acylation was explored, however only poor reactivity was observed in CH_3_CN solvent. After removal of CH_3_CN, however, HFIP (1,1,1,3,3,3-hexafluoroisopropanol) facilitated conversion to the corresponding diarylketones **26**–**28** (39–59% yield) without further purification of the intermediate or removal of iron salts.^56^

To gain some insight into the mechanism of this reaction, we sought to intercept the proposed acyl radical intermediate. In the presence of TEMPO under otherwise identical conditions, we observed a significant reduction in conversion to the corresponding acid chloride (**30**) and obtained the amide product **4** in only 31% yield by ^1^H NMR. We also observed the formation of TEMPO-trapped adduct **29** whose identity was supported by HRMS (Scheme 2, top). TEMPO-trapped adduct **29** was also observed in the absence of TCCA under similar conditions. These results are consistent with the proposed acyl radical and fast radical chlorination by TCCA. We also intercepted the acyl radical with alkene acceptors, which we reported previously under blue light irradiation with catalyst **1b**.^19^ As shown in Scheme 3A, the alkylation reaction was very sluggish in comparison to acyl chloride formation, providing only a 14% yield of product **33** after 36 h of irradiation at 500 nm. Performing the reaction with blue light led to minimal improvement, with all catalysts **1b**–**1d** giving comparable yields (16–20%), highlighting the low efficiency of aldehydes in this reaction. Performing the alkylation reaction with THF, a more reactive substrate, we observed lower yields with 500 nm light and a slight improvement with 456 nm light (up to 40% yield of **36** using **1d**, Scheme 3B). Catalyst **1b** gave the highest yield (52%), but overall LMCT with blue light to generate bromine radical is comparable for catalysts **1b**–**1d**. These results are consistent with an LMCT mechanism and demonstrate productive photocatalytic alkylation with an iron catalyst and cyan light, which has not previously been reported.

**Scheme 2:**
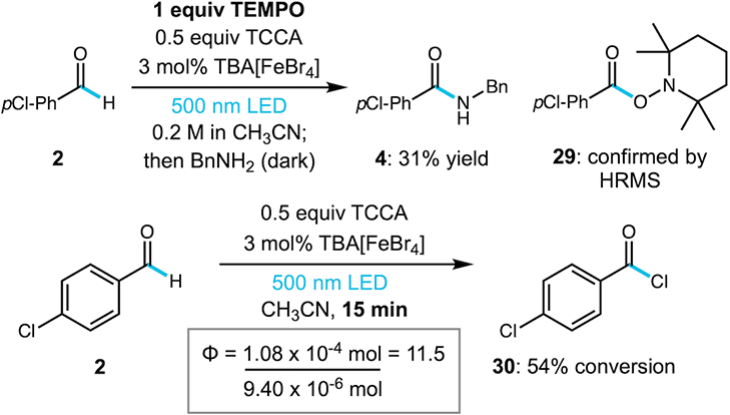
TEMPO Trapping and Quantum Yield Experiments under Cyan Light Irradiation

**Scheme 3:**
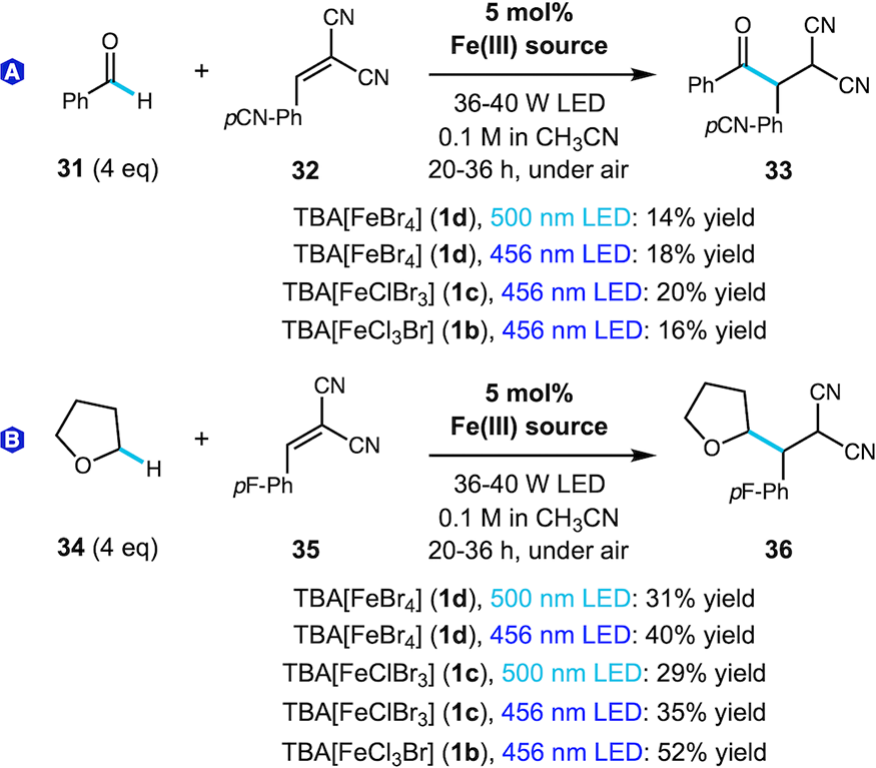
Catalyst and Light Source Studies for C–H Alkylation^a^

a Reactions performed on a 0.1 mmol scale using one LED lamp over 20–36 h with fan cooling using a Hepatochem PhotoRedOx box. Yields determined by ^1^H NMR using mesitylene as an internal standard.

The significant difference in rate and efficiency between the chlorination and alkylation reactions raises questions about the catalysis mechanism we initially envisioned based on our previous work. Furthermore, the chlorination reaction is notably faster under air than under inert atmosphere (only 39–56% product **4** under N_2_ after the same reaction time as Table 1; see Table S1), which was not observed for most alkylation reactions.^19^ This suggests a role for oxygen in accelerating the reaction, perhaps facilitating the turnover of Fe­(II) species. We considered the possibility of a chain process that is initiated by LMCT-promoted bromine radical generation, which was probed using quantum yield measurements.^57^ Actinometry experiments adapted from the work of Pitre and Scaiano with potassium ferrioxalate were conducted at 500 nm to determine the photon flux from the 500 nm LED lamp. With these results, quantum yield Φ was calculated to be 11.5 at an early time point (15 min) with 54% conversion (Scheme 2, bottom). In other words, 11.5 equiv of product are formed for every photon absorbed by the reaction mixture, a result that is most consistent with a chain propagation mechanism.

Based on these experiments and our previous work, the proposed mechanism for iron-promoted aldehyde halogenation is presented in Figure 3. Excitation of the tetrahedral Fe­(III) tetrahalide through LMCT produces bromine radical **I** and anionic Fe­(II) byproduct **II** (step a). The bromine radical undergoes HAT with the aldehyde substrate **31** (step b), generating the corresponding acyl radical **III** and HBr (BDE_H–Br_ = 88 kcal/mol).^58,59^ Radical **III** reacts with the chlorinating reagent TCCA (or NCS) to generate acid chloride **37** and imide radical **IV** (step c). Propagation of the chain process continues with HAT between N-centered radical **IV** and the aldehyde (step d). The Fe­(II) byproduct **II** can return to its active Fe­(III) state **1d** via SET with dissolved oxygen (step e) and coordination of bromide, consistent with the rate enhancement of the reaction under air. A closed catalytic cycle involving SET between Fe­(II) species **II** and imide radical **IV** is likely a minor pathway, if present. The role of ferrate salts **1c** and **1d** is to initiate the reaction using low energy cyan and green light, which do not efficiently promote the reaction directly (see Table 1). The possibility of radical ligand transfer of halogen from Fe–X species as described by West and co-workers is less consistent with our observations, but cannot be completely excluded.^35,60–62^

**Figure 3:**
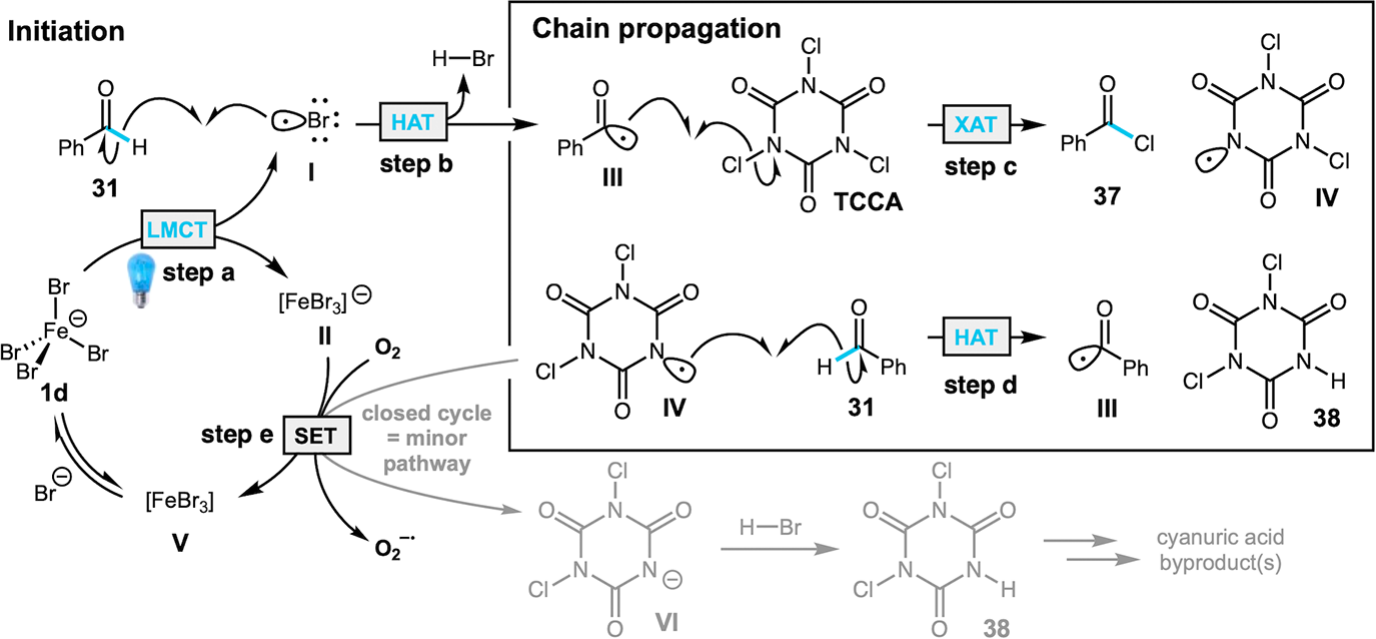
Proposed chain mechanism of photocatalytic halogenation.

We report the use of simple, bench-stable iron tetrahalide salts for the conversion of aldehydes to amides, esters, and ketones in a one-pot reaction promoted by low energy visible light. The newly reported mixed halide catalyst TBA­[FeClBr_3_] (**1c**) is highly effective up to 500 nm (cyan light), while tetrabromide catalyst **1d** pushes the productive reactivity out to 525 nm (green light) with only a marginal loss in reactivity. The scope of the reaction includes a variety of aldehydes, amines, alcohols, and electron-rich arenes. Mechanistic studies provide support for a chain process involving acyl radical intermediates that is initiated by LMCT excitation of the ferrate salt to produce a halogen radical. Under these conditions, TCCA or NCS serves as both the stoichiometric oxidant and HAT reagent in the chain process. The goal of extending the reactivity of Fe­(III) halide salts to longer wavelength visible light has been achieved; however, reaction efficiency is reduced compared to previous transformations with higher energy light (≤456 nm). Efforts to more clearly define the role of air in the reaction and to improve the efficiency for reactions beyond radical chlorination are ongoing.

## Supplementary Material



## Data Availability

The data underlying this study are available in the published article and its Supporting Information.

## Notes

TThe Supporting Information is available free of charge at https://pubs.acs.org/doi/10.1021/acs.orglett.5c04928.

Experimental procedures, characterization data, ^1^H and ^13^C NMR spectra of all new compounds (PDF)
