# Supplementary material for: Ligand-Enabled Photocatalytic Reactivity of Iron(III) Halide Salts with Cyan and Green Light
Source: Org Lett. 2026 Jan 14;28(4):1200–6. doi: 10.1021/acs.orglett.5c04928 (PMC12865799; doi:10.1021/acs.orglett.5c04928)

Supporting Information

Ligand-Enabled Photocatalytic Reactivity of Iron(III) Halide Salts with Cyan and Green Light

Mikayla M. Wymore and David B.C. Martin*

Corresponding Author: david-martin@uiowa.edu

Department of Chemistry, University of Iowa, Iowa City, Iowa 52242, United States.

Contents

A. General Methods……………………………………………………………...….S3
B. General Procedures…………………………………………………………….....S3
   I. Photochemical Reactions with TBA[$FeBr_4$]………………………………….......S3
   II. Synthesis of Ferrate Catalysts……………………………………………............S5
   III. Synthesis of Methyl 3-Aminopropanoate Hydrochloride……………………….......S6
   IV. UV-Vis Studies…………………………………………………………………......S6
C. Optimization Details………………………………………………………….........S8
   I. General Procedure for Optimization of Reactions with Ferrate Catalyst……......S9
   II. Optimization Results…………………………………………………………......S9
   III. Incompatible Substrates……………………………………………….……..S10
D. Mechanistic Studies…………………………………………………………..…...S10
   I. TEMPO Adduct……………………………………………………………..….…S10
   II. Actinometry Studies……………………………………………………….........S10
E. Characterization Data of Products……………………………………………….…...S11
F. References……………………………………………………………………………..S22
G. NMR Spectra…………………………………………………………………….......S25

General Methods:

All reactions were carried out in an 8 mL vial charged with a magnetic stir bar under air, unless otherwise noted. All solvents were dried by passage through columns of activated alumina or distilled and stored under nitrogen over freshly activated 4 Å sieves or otherwise freshly distilled. All starting materials were prepared according to known literature procedures or used as obtained from commercial sources, unless otherwise indicated. Reactions were monitored by thin-layer chromatography (TLC) and carried out on 0.25 mm coated commercial silica gel plates (Analtech TLC Uniplates, F254 precoated glass plates) using UV light as the visualizing agent. Unless otherwise indicated, silica gel chromatography was performed using flash chromatography on P60 silica.

^{1}H and ^{13}C NMR spectra were recorded on a Bruker DRX 400 MHz, a Bruker AVANCEIII 400 MHz, or AVANCE 500 MHz spectrometer and were internally referenced to residual protio solvent signal (note: $CDCl_3$ referenced at δ 7.26 ppm for 1H NMR and δ 77.16 ppm for ^{13}C NMR, respectively). Data for ^{1}H NMR are reported as follows: chemical shift (δ ppm), multiplicity (s = singlet, d = doublet, t = triplet, q = quartet, m = multiplet, app=apparent), coupling constant (Hz), and integration. Data for ^{13}C NMR are reported in terms of chemical shift; no special nomenclature is used for equivalent carbons. High-resolution mass spectrometry (HRMS) data were recorded on a Thermo Q-Exactive instrument (hybrid quadrupole Orbitrap) using direct injection of samples in dichloromethane diluted into acetonitrile/water (1:1) into the electrospray source (ESI) with positive ionization or negative ionization (as noted) or on an Agilent 7250 GCMS Q-TOF instrument with electron ionization (EI) using direct injection of samples in dichloromethane, listed as GC-HRMS. UV-Vis experiments were carried out with an Agilent Cary 5000, with a total wavelength range of 175-3300 nm, but collecting data from 200 nm to 800 nm.

A. General Procedures

I. Photochemical Reactions with TBA[$FeBr_4$]

General Procedure A: A typical procedure for photoreactions with a tetrabutylammonium iron catalyst can be conducted as follows: aldehyde (0.2 or 0.6 mmol, 1.0 equiv.), ferrate catalyst (3 mol%), trichloroisocyanuric acid (0.1 or 0.3 mmol, 0.5 equiv.), and dry acetonitrile (1.0 mL or 3.0 mL, 0.2M) were added to an 8-mL vial equipped with a stir bar. The vial was capped with the manufacturer provided cap and oxygen was not excluded. The vials were then stirred at room temperature for 4-5h under irradiation of a 500 nm LED and 7h under irradiation of a 525 nm Kessil LED. Reactions are approximately 6 cm from the Kessil LED lamps. After, nucleophilic amines (0.6 or 1.8 mmol, 3 equiv.) were added and then stirred at room temperature in the dark for an additional 2h.

General Procedure B: A typical procedure for photoreactions with the tetrabutylammonium iron catalyst can be conducted as follows: aldehyde (0.2 or 0.6 mmol, 1.0 equiv.), ferrate catalyst (3 mol%), *N*-chlorosuccinimide (0.26 mmol, 1.3 equiv.), and dry acetonitrile (1.0 mL or 3.0 mL,

0.2M) were added to an 8-mL vial equipped with a stir bar. The vial was capped with the manufacturer provided cap and oxygen was not excluded. The vials were then stirred at room temperature for 20h under irradiation of a 456 nm Kessil LED. After, nucleophilic amines (0.6 or 1.8 mmol, 3 equiv.) were added and then stirred at room temperature in the dark for an additional 2h.

General Procedure C: A typical procedure for photoreactions with the tetrabutylammonium iron catalyst can be conducted as follows: aldehyde (0.2 or 0.6 mmol, 1.0 equiv.), ferrate catalyst (3 mol%), trichloroisocyanuric acid (0.1 or 0.3 mmol, 0.5 equiv.), and dry acetonitrile (1.0 mL or 3.0 mL, 0.2M) were added to an 8-mL vial equipped with a stir bar. The vial was capped with the manufacturer provided cap and oxygen was not excluded. The vials were then stirred at room temperature for 4-5h under irradiation of a 500 nm LED or 7h under irradiation of a 525 nm Kessil LED. After, alcohols (0.6 mmol, 3 equiv.), DMAP (0.1 mmol, 0.5 equiv.), and DIPEA or pyridine (0.75 mmol, 3.75 equiv.) were added and then stirred at room temperature in the dark for an additional 16h. For products **22-24**, phenols (0.6 mmol, 3 equiv.) and triethylamine or pyridine (0.6 mmol, 3 equiv.) were added and stirred at room temperature in the dark for an additional 3h.

General Procedure D: A typical procedure for photoreactions with the tetrabutylammonium iron catalyst can be conducted as follows: aldehyde (0.2 or 0.6 mmol, 1.0 equiv.), ferrate catalyst (3 mol%), trichloroisocyanuric acid (0.1 or 0.3 mmol, 0.5 equiv.), and dry acetonitrile (1.0 mL or 3.0 mL, 0.2M) were added to an 8-mL vial equipped with a stir bar. The vial was capped with the manufacturer provided cap and oxygen was not excluded. The vials were then stirred at room temperature for 4-5h under irradiation of a 500 nm LED or 7h under irradiation of a 525 nm Kessil LED. After, acetonitrile was removed *in vacuo* and replaced with HFIP (0.2M). Nucleophilic arenes (0.6 mmol, 3 equiv.) were added and then stirred at room temperature in the dark for an additional 16h.

Aqueous workup for Select Products **9-11**, **13**, and **21**

Acetonitrile was removed in vacuo and replaced with either DCM or EtOAc. Organic layers were washed 3 times with saturated $NaHCO_3$ or NaOH and 1 time with distilled water. Combined organic layers were dried over Na_2SO_4, filtered, and concentrated in vacuo to provide crude material for column purification.

Aqueous workup for Selected Product **8**

Acetonitrile was removed in vacuo and replaced with either DCM or EtOAc. Organic layers were washed 4 times with 1M HCl and 1 time with distilled water. Combined organic layers were dried over Na_2SO_4, filtered, and concentrated in vacuo to provide crude material for column purification.

Purification for Procedures A-D
The reaction mixtures were then concentrated *in vacuo* and the resulting crude material was purified using column chromatography. All isolated products were further characterized by ^{1}H and ^{13}C nuclear magnetic resonance (NMR) spectroscopy. New compounds (**12** and **21**) were also characterized by infrared spectroscopy and mass spectrometry.

General Photochemical Methods

A typical setup for a photoreaction at 456 nm in the Martin Lab utilizes an EvoluChem PhotoRedOx Box from Hepatochem (part number HCK1006-01-016, https://hepatochem.com/photoreactors-leds-%20%20accessories/photoredox-box/?gad_source=1&gclid=Cj0KCQiAqL28BhCrARIsACYJvkeh6AQIqAcx_jsK5Zp6ga-%20%200vP38EMEX8GraU8zTFJsSkIXnEF7ZYD0aAn2DEALw_wcB%20%20)%20(pictured). The PhotoRedOx box is placed on a stir plate to provide stirring. 40W PR160L lamps (456, 467, and 525 nm) from Kessil (https://kessil.com/products/science_PR160L.php) are used in experiments. Emission spectra provided by Kessil are included below. A typical setup for a photoreaction at 500 nm in the Martin Lab utilizes a 36W cyan (~495-500 nm) LED.

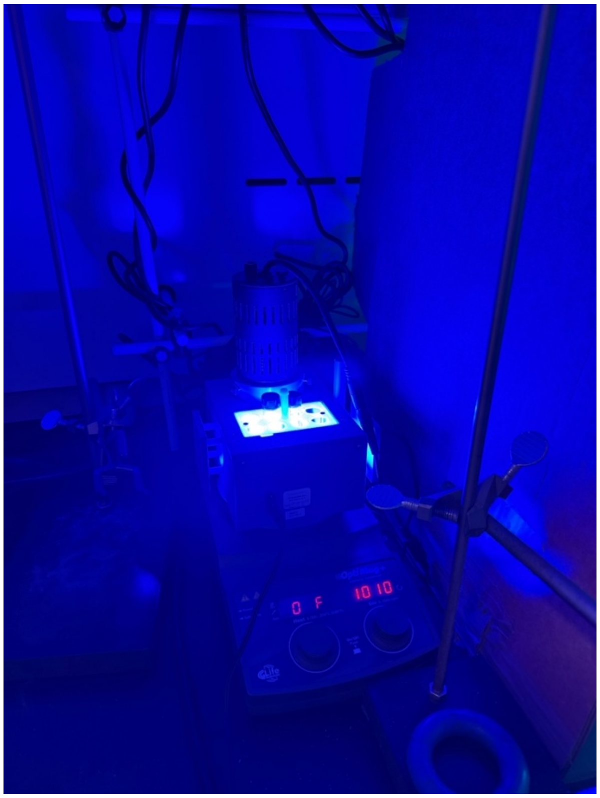

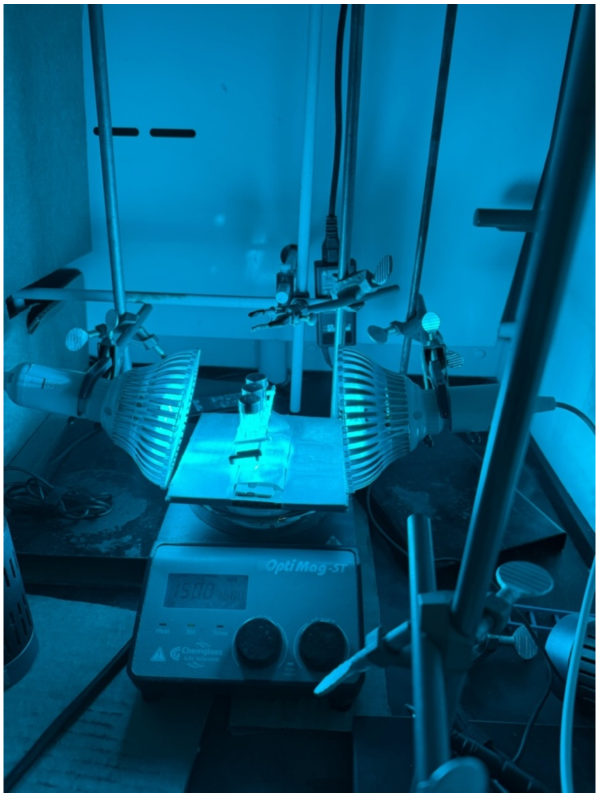

II. Synthesis of Ferrate Catalysts[1,2]

FeX_3 (1 eq) + TBABr (1 eq) → (MeCN (1M), rt, 1h) TBA[FeX_3Br]

Anhydrous iron trihalide (3.0 mmol, 1 eq) and equimolar tetrabutylammonium bromide were added to a round bottom flask charged with a magnetic stir bar. Distilled acetonitrile (3 mL,

1M) was added, and the reaction was stirred at room temperature for 1 hour. The reaction mixture was then concentrated in vacuo and used without further purification.

III. Synthesis of Methyl 3-Aminopropanoate Hydrochloride

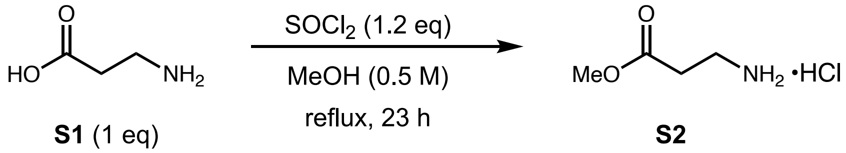

S1 (1 eq) **S2**

Methyl 3-aminopropanoate was prepared from β-alanine according to the report from Wille and colleagues.[3] Thionyl chloride (6.7 mmol, 1.2 equiv.) was added dropwise to a solution of β-alanine **S1** (5.6 mmol, 1 equiv.) in methanol (0.5M) that was cooled to 0 ℃ .. The reaction mixture was stirred at reflux for 23 h, then the solvent was removed *in vacuo*. The crude product, methyl 3-aminopropanoate hydrochloride, was obtained as a pale-yellow solid (727.5 mg, 93%) and used without additional purification.

IV. UV-Vis Studies

General Procedures:

Samples were prepared using volumetric flasks. 3 mL of the sample was transferred to a quartz cuvette for analysis.

Sample Preparation: (0.24 mM TBA[$FeBr_4$] in MeCN)

TBA[$FeBr_4$] (3.7 mg, 5.99 mmol) was dissolved in 25 mL MeCN to prepare a 0.24 mM solution.

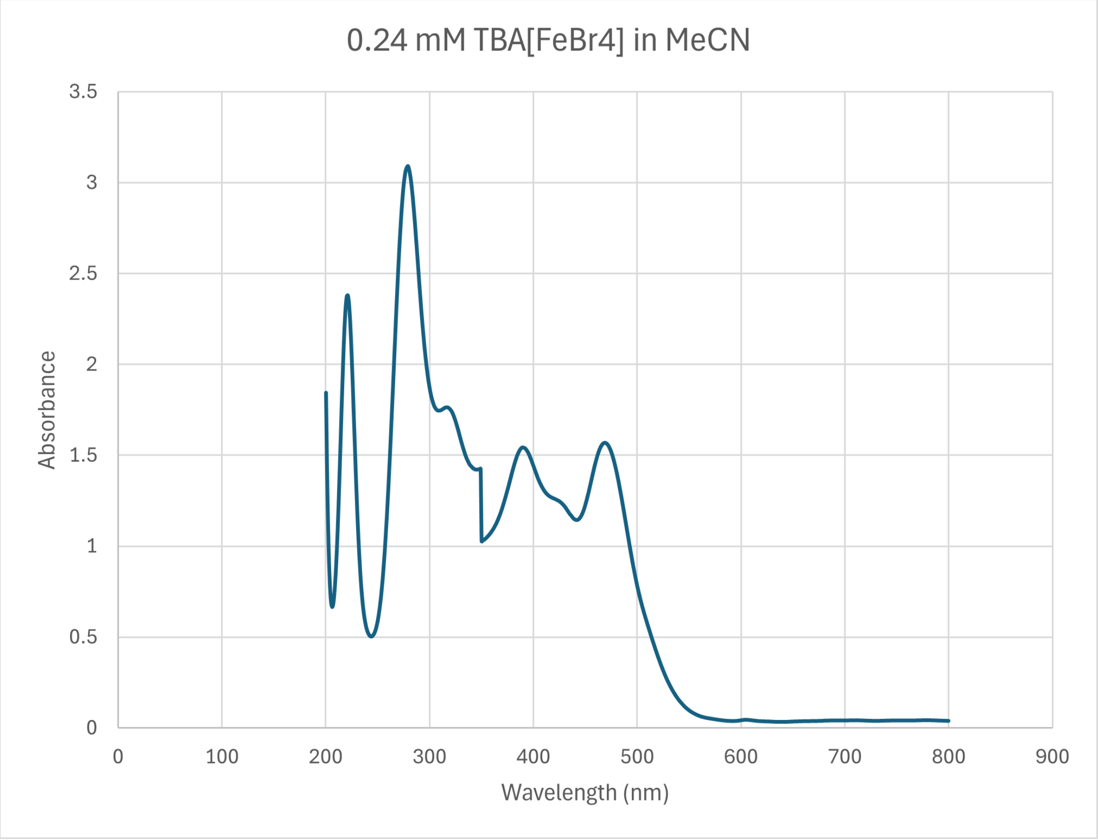

Sample Preparation: (0.24 mM TBA[$FeBr_3Cl$] in MeCN)

TBA[$FeBr_3Cl$] (3.4 mg, 5.99 mmol) was dissolved in 25 mL MeCN to prepare a 0.24 mM solution.

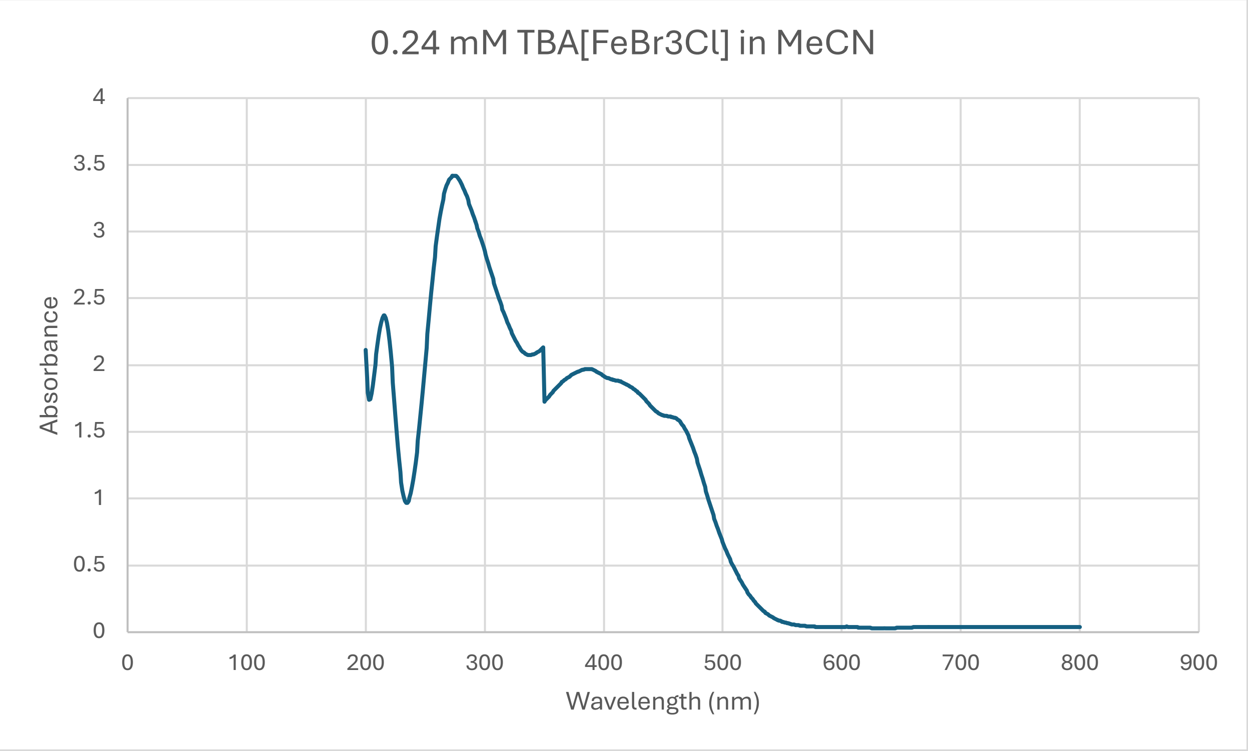

Sample Preparation: (0.1M TCCA in MeCN)

TCCA (0.069 g, 0.3 mmol) was dissolved in 3 mL MeCN to prepare a 0.1M solution. Inclusion of an aldehyde starting material did not show additional absorbance at either 500 or 525 nm.

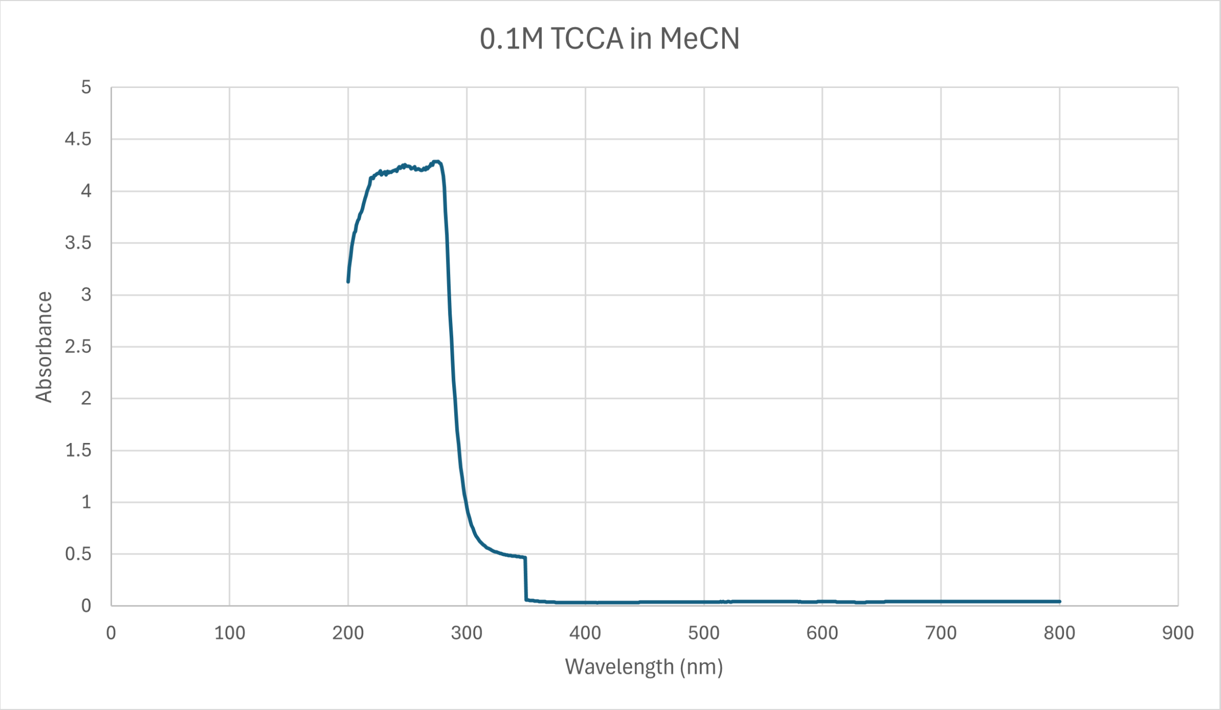

B. Optimization Details

I. General Procedure for Optimization of Reactions with Ferrate Catalyst

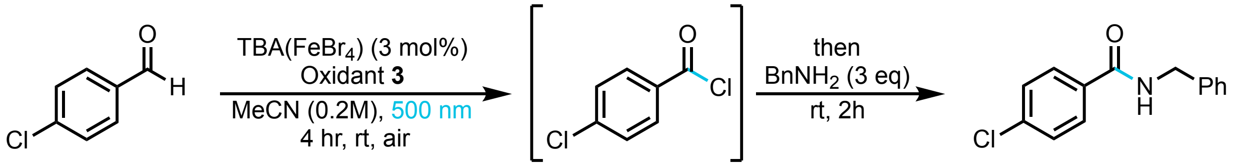

(0.2 mmol, 1 equiv.)

2

4

A typical procedure for photoreactions with the tetrabutylammonium iron tetrabromide catalyst can be conducted as follows: aldehyde (0.2 mmol, 1.0 equiv.), TBA[$FeBr_4$] (3 mol%), trichloroisocyanuric acid (0.1 mmol, 0.5 equiv.), and dry acetonitrile (1.0 mL, 0.2M) were added to an 8-mL vial equipped with a stir bar. The vial was capped with the manufacturer provided cap and oxygen was not excluded. The vials were then stirred at room temperature for 4h under irradiation of a 500 nm LED. After, nucleophilic amines (0.6 or 1.8 mmol, 3 equiv.) were added and then stirred at room temperature in the dark for an additional 2h. After, 0.2 mmol of dibromomethane or mesitylene were added to the reaction mixture and stirred. An aliquot was obtained and diluted with $CDCl_3$ to obtain a yield based on ^1H NMR spectroscopy.

II. Optimization Results

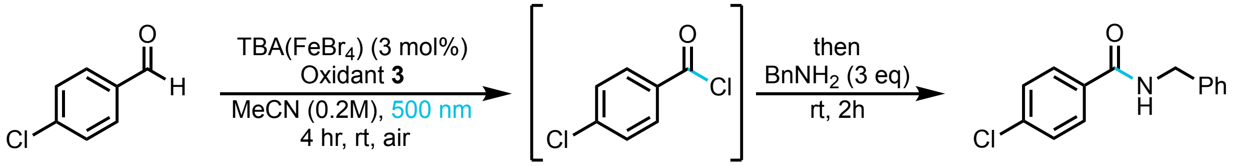

(0.2 mmol, 1 equiv.)

2

4

Table S1: Optimization of ferrate catalysts and oxidants.

Entry	Fe(III) Catalyst	LED (nm)	Oxidant (**3**)	Atmosphere	Yield (**4**, %)
1	TBA[$FeCl_3Br$]	390	NCS	Air	75
2	TBA[$FeCl_3Br$]	390	NBS	Air	27
3	TBA[$FeCl_3Br$]	456	NCS	Air	78
4	TBA[$FeCl_3Br$]	456	NBS	Air	16
5	TBA[$FeCl_3Br$] (3 mol%)	456	NCS	Air	74
6	TBA[$FeCl_3Br$] (5 mol%)	456	NCS	Air	76
7	TBA[$FeCl_3Br$]	456	NCS	Air	72
8	TBA[$FeCl_3Br$]	456	NCS	N_2	39
9	TBA[$FeBr_4$] (3 mol%)	500	TCCA	Air	100
10	TBA[$FeBr_4$] (5 mol%)	500	TCCA	Air	100
11	TBA[$FeBr_4$]	500	TCCA	Air	99.9
12	TBA[$FeBr_4$]	500	TCCA	N_2	56

13	TBA[FeBr4]	500	--	Air	0
14	TBA[FeBr4]	--	TCCA	Air	0

III. Incompatible Substrates

The following substrates led to low yields or mixtures of products.

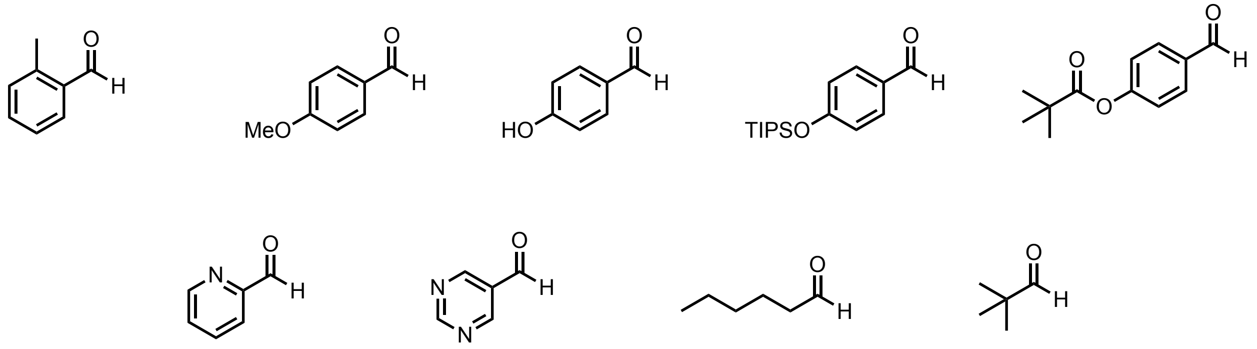

***Figure S1**: Incompatible substrates in this work.*

C. Mechanistic Studies

I. TEMPO Adduct

Made according to General Procedure A replacing TCCA with TEMPO (0.6 mmol, 1 equiv.). TEMPO adduct identified by HRMS.

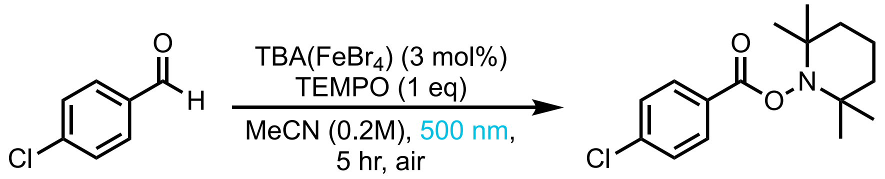

2
1 eq, 0.6 mmol

29
Calcd $[M+H]^+$ = 296.1412
Found = 296.1413

HRMS (p ESI + FTMS): Calcd for $C_{16}H_{22}ClNO_2$ $[M+H]^+$ = 296.1412, found 296.1413.

II. Actinometry Studies

In volumetric flasks, 0.15M potassium ferrioxalate and 0.1% buffered 1,10-phenanthroline standards were prepared. Considering the light sensitivity of this experiment, both standards are stored in amber bottles.

In a typical experiment, five samples containing 3 mL of 0.15M potassium ferrioxalate were made. Four of these samples were irradiated for 45s, 60s, 75s, and 120s. The fifth was left in the dark as a control. Following irradiation, 500 μL of 0.1% 1,10-phenanthroline were added to all five samples. These samples were allowed to develop in the dark for 30 min before acquiring absorption data.

$$\Delta A510 = A_{light} - A_{dark} = 2.28363 - 0.52702 = 1.75661$$

By UV-vis spectroscopy, the absorption of the five samples were obtained at 510 nm and the optical difference between irradiated and non-irradiated samples was determined. From this data, and with the known molar absorptivity (ε = 11,100 M^{-1} cm^{-1}), the moles of Fe^{2+} can be determined by the Beer-Lambert law.

$$c = \frac{A}{\varepsilon * l} = \frac{1.75661}{11100 * 1} = 1.5825 * 10^{-4} M\ Fe^{2+}$$

$$Moles\ of\ Fe^{2+} = M * V = (1.5825 * 10^{-4} M) * (3.5 * 10^{-3} L) = 5.5389 * 10^{-7}\ moles$$

Based on the moles of Fe^{2+} produced in this entry and the known quantum yield of ferrioxalate (0.90 at 502 nm), the photon flux can be determined. In this equation, Φ corresponds to the quantum yield of ferrioxalate at 502 nm, t corresponds to the irradiation time, and F corresponds to the fraction of light absorbed ($1\text{-}10^{-A}$ = 0.9825).

$$Photon\ flux = \frac{moles\ of\ Fe^{2+}}{\Phi * t * F} = \frac{5.5389 * 10^{-7}}{0.90 * 60s * 0.9825} = 10.4398 * 10^{-9} mol/s$$

With the photon flux for potassium ferrioxalate calculated, the quantum yield of the experiment reported here can be calculated.

$$\Phi = \frac{moles\ of\ acyl\ chloride\ produced\ in\ time\ (h)}{moles\ of\ photon\ absorbed\ per\ unit\ time}$$

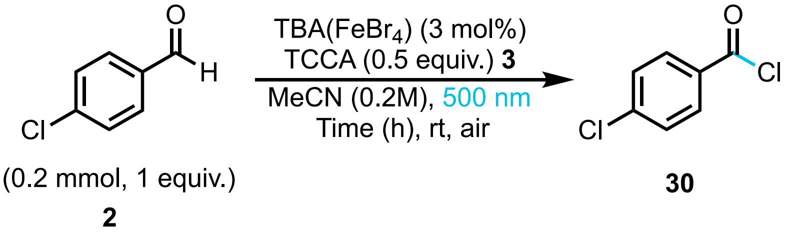

Table S2: Calculation of quantum yield at two time points.

Entry	Time (h)	Amount of Acyl Chloride Produced	Quantum Yield, Φ
1	15 min	$1.08 * 10^{-4}\ moles$	11.50
2	4 h	$1.64 * 10^{-4}\ moles$	1.09

D. Characterization Data of Products

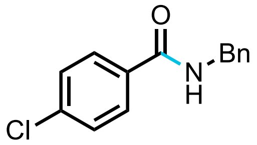

4

Made according to General Procedure A with TBA[$FeBr_4$] at 500 and 525 nm. Purified by column chromatography with 10:1 to 4:1 hexanes/EtOAc. Isolated as a white solid (106.3 mg, 72%) with TBA[$FeBr_4$] at 500 nm and (28.8 mg, 59%) at 525 nm.

Made according to General Procedure A with TBA[$FeBr_3Cl$] at 500 nm. Purified by column chromatography with 10:1 to 4:1 hexanes/EtOAc. Isolated as an off-white solid (31.6 mg, 65%).

^{1}H NMR (400 MHz, Chloroform-*d*) δ 7.76 – 7.70 (m, 2H), 7.44 – 7.28 (m, 7H), 6.32 (s, 1H), 4.64 (d, *J* = 5.63 Hz, 2H).

^{13}C NMR (101 MHz, Chloroform-*d*) δ 166.4, 138.1, 137.9, 132.9, 129.0, 128.6, 128.1, 127.9, 44.3.
NMR data was consistent with literature values.[5]

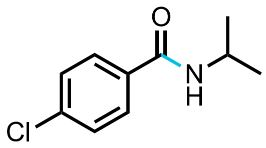

5

Made according to General Procedure A with TBA[$FeBr_4$] at 500 nm. Purified by column chromatography with 10:1 to 4:1 hexanes/EtOAc. Isolated as an white solid (87.7 mg, 73%).

^{1}H NMR (400 MHz, Chloroform-*d*) δ 7.70 – 7.68 (m, 2H), 7.41 – 7.39 (m, 2H), 5.85 (s, 1H), 4.32 – 4.23 (m, 1H), 1.27 – 1.26 (d, 6H).

^{13}C NMR (101 MHz, Chloroform-*d*) δ 165.7, 137.6, 133.5, 128.9, 128.4, 42.2, 23.0.
NMR data was consistent with literature values.[6]

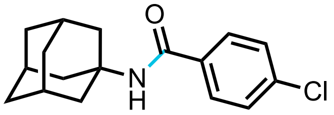

6

Made according to General Procedure A with TBA[$FeBr_4$] at 500 nm. Purified by column chromatography with 10:1 hexanes/EtOAc. Isolated as a white solid (111.8 mg, 64%).

^{1}H NMR (400 MHz, Chloroform-*d*) δ 7.65 (d, *J* = 8.50 Hz, 2H), 7.41 – 7.35 (m, 2H), 5.72 (s, 1H), 2.12 (s, 10H), 1.73 (s, 6H).

^{13}C NMR (101 MHz, Chloroform-*d*) δ 165.6, 137.2, 134.5, 128.7, 128.3, 52.6, 41.7, 36.4, 29.6.
NMR data was consistent with literature values.[7]

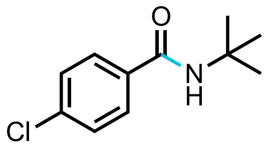

7

Made according to General Procedure A with $TBA[FeBr_4]$ at 500 and 525 nm. Purified by column chromatography with 10:1 to 4:1 hexanes/EtOAc. Isolated as a white solid (94.2 mg, 74%) with $TBA[FeBr_4]$ at 500 nm and (24.8 mg, 59%) at 525 nm.

^{1}H NMR (400 MHz, Chloroform-*d*) δ 7.66 (d, *J* = 8.57 Hz, 2H), 7.38 (d, *J* = 8.53 Hz, 2H), 5.87 (s, 1H), 1.47 (s, 9H).

^{13}C NMR (101 MHz, Chloroform-*d*) δ 165.9, 137.4, 134.4, 128.8, 128.3, 51.9, 29.0.
NMR data was consistent with literature values.[8]

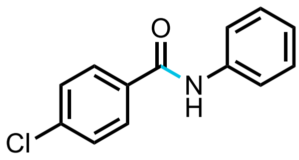

8

Made according to General Procedure A with $TBA[FeBr_4]$ at 500. Purified by column chromatography with 4:1 hexanes/EtOAc. Isolated as a beige solid (76.3 mg, 55%).

^{1}H NMR (400 MHz, Chloroform-*d*) δ 7.85 – 7.79 (m, 2H), 7.77 (s, 1H), 7.66 – 7.58 (m, 2H), 7.50 – 7.43 (m, 2H), 7.43 – 7.34 (m, 2H), 7.21 – 7.13 (m, 1H).

^{13}C NMR (101 MHz, Chloroform-*d*) δ 164.8, 138.3, 137.8, 133.5, 129.3, 129.2, 128.6, 125.0, 120.4.
NMR data was consistent with literature values.[9]

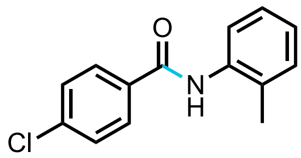

9

Made according to General Procedure A with $TBA[FeBr_4]$ at 500 nm. Purified by column chromatography with 4:1 hexanes/EtOAc. Isolated as a beige solid (79.6 mg, 54%).

^{1}H NMR (400 MHz, Chloroform-*d*) δ 7.91 (d, *J* = 8.23 Hz, 1H), 7.86 – 7.79 (m, 2H), 7.59 (s, 1H), 7.51 – 7.45 (m, 2H), 7.30 – 7.21 (m, 4H), 7.14 (td, *J* = 7.47, 1.27 Hz, 1H), 2.34 (s, 3H).

^{13}C NMR (101 MHz, Chloroform-*d*) δ 164.8, 138.2, 135.6, 133.4, 130.8, 129.9, 129.2, 128.7, 127.0, 125.8, 123.6, 18.0.
NMR data was consistent with literature values.[10]

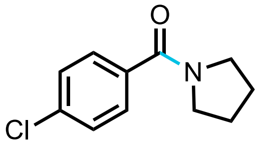

10

Made according to General Procedure A with TBA[$FeBr_4$] at 500 nm. Purified by column chromatography with 1:4 hexanes/EtOAc. Isolated as a clear to pale yellow liquid (68.5 mg, 54%).

^{1}H NMR (400 MHz, Chloroform-*d*) δ 7.45 (d, *J* = 8.48 Hz, 2H), 7.34 (d, *J* = 8.50 Hz, 2H), 3.61 (t, *J* = 6.94 Hz, 2H), 3.39 (t, *J* = 6.55 Hz, 2H), 1.98 – 1.80 (m, 4H).

^{13}C NMR (101 MHz, Chloroform-*d*) δ 168.6, 135.9, 135.6, 128.8, 128.6, 49.7, 46.4, 26.5, 24.5.
NMR data was consistent with literature values.[11]

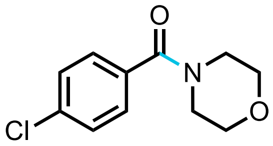

11

Made according to General Procedure A with TBA[$FeBr_4$] at 500 nm. Purified by column chromatography with 1:4 hexanes/EtOAc. Isolated as a clear to pale yellow liquid (93.7 mg, 69.0%).

^{1}H NMR (400 MHz, Chloroform-*d*) δ 7.38 (d, *J* = 8.57 Hz, 2H), 7.34 (d, *J* = 8.57 Hz, 2H), 3.56 (d, *J* = 93.07 Hz, 8H).

^{13}C NMR (101 MHz, Chloroform-*d*) δ 169.4, 136.1, 133.7, 128.9, 128.7, 66.9.
NMR data was consistent with literature values.[12]

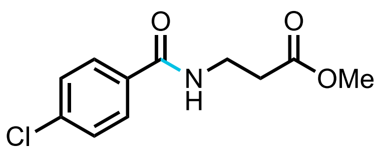

12

Made according to General Procedure A with TBA[$FeBr_4$] at 500 nm. Purified by column chromatography with 1:1 hexanes/EtOAc. Isolated as a beige solid (32.2 mg, 67.0%).

^{1}H NMR (400 MHz, Chloroform-*d*) δ 7.69 (d, *J* = 8.60 Hz, 2H), 7.38 (d, *J* = 8.55 Hz, 2H), 6.90 (s, 1H), 3.70 (d, *J* = 4.28 Hz, 5H), 2.64 (t, *J* = 5.88 Hz, 2H).

^{13}C NMR (101 MHz, Chloroform-*d*) δ 173.5, 166.4, 137.9, 132.8, 128.5, 35.5, 33.7.

IR (ATR, neat, cm^{-1}): 3290, 2951, 1731, 1635, 1594, 1544, 1486, 1437, 1252, 1218, 845.

HRMS (p ESI + FTMS): Calcd for $C_{11}H_{13}ClNO_3$ $[M+H]^+$ = 242.0578, found 242.0580.

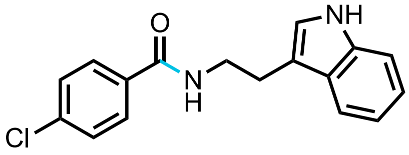

13

Made according to General Procedure A with TBA[$FeBr_4$] at 500 nm. Purified by column chromatography with 2:1 hexanes/EtOAc. Isolated as a beige solid (97.1 mg, 54%).

^{1}H NMR (400 MHz, Chloroform-*d*) δ 8.06 (s, 1H), 7.64 (d, *J* = 7.92 Hz, 1H), 7.62 – 7.56 (m, 2H), 7.40 (d, *J* = 8.14 Hz, 1H), 7.38 – 7.32 (m, 2H), 7.26 – 7.19 (m, 1H), 7.17 – 7.10 (m, 1H), 7.08 (d, *J* = 2.00 Hz, 1H), 6.14 (s, 1H), 3.80 (q, *J* = 6.33 Hz, 2H), 3.10 (t, *J* = 6.60 Hz, 2H).

^{13}C NMR (101 MHz, Chloroform-*d*) δ 166.5, 137.7, 136.6, 133.2, 128.9, 128.4, 127.4, 122.5, 122.2, 119.8, 118.9, 113.1, 111.5, 40.5, 25.4.
NMR data was consistent with literature values.[13]

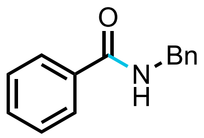

14

Made according to General Procedure A with TBA[$FeBr_4$] at 500 nm. Purified by column chromatography with 10:1 hexanes/EtOAc. Isolated as a white solid (21.8 mg, 52%).

Made from benzyl alcohol according to General Procedure A with TCCA (0.2 mmol, 1 equiv.) and isolated (23.0 mg, 44% yield).

^{1}H NMR (400 MHz, Chloroform-*d*) δ 7.82 – 7.76 (m, 2H), 7.53 – 7.46 (m, 1H), 7.46 – 7.39 (m, 2H), 7.36 (d, *J* = 4.39 Hz, 4H), 7.33 – 7.27 (m, 1H), 6.47 (s, 1H), 4.65 (d, *J* = 5.66 Hz, 2H).

^{13}C NMR (101 MHz, Chloroform-*d*) δ 167.5, 138.3, 134.5, 131.7, 128.9, 128.7, 128.1, 127.8, 127.1, 44.3.
NMR data was consistent with literature values.[6]

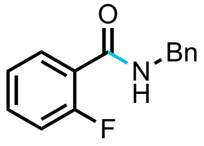

15

Made according to General Procedure A with TBA[$FeBr_4$] at 500 and 525 nm. Purified by column chromatography with 4:1 hexanes/EtOAc. Isolated as a beige solid (40.1 mg, 88%) at 500 nm and (29.2 mg, 64%) at 525 nm. On a 1 mmol scale, isolated as a beige solid (131.8 mg, 58% yield) at 500 nm.

^{1}H NMR (400 MHz, Chloroform-*d*) δ 8.14 (td, *J* = 7.92, 1.93 Hz, 1H), 7.47 (tdd, *J* = 7.50, 5.21, 1.92 Hz, 1H), 7.39 – 7.32 (m, 4H), 7.32 – 7.23 (m, 2H), 7.15 – 6.99 (m, 2H), 4.69 (dd, *J* = 5.68, 1.52 Hz, 2H).

^{13}C NMR (101 MHz, Chloroform-*d*) δ 163.4 (d, *J* = 3.12 Hz), 160.8 (d, *J* = 247.16 Hz), 138.1, 133.5 (d, *J* = 9.36 Hz), 132.3 (d, *J* = 2.26 Hz), 128.9, 127.9, 127.7, 125.0 (d, *J* = 3.37 Hz), 121.1 (d, *J* = 11.52 Hz), 116.1 (d, *J* = 24.76 Hz), 44.2.
NMR data was consistent with literature values.[14]

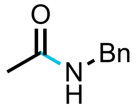

16

Made according to General Procedure A with TBA[$FeBr_4$] at 500 nm. Purified by column chromatography with 4:1 hexanes/EtOAc. Isolated as an off-white solid (78.9 mg, 88% yield).

^{1}H NMR (500 MHz, Chloroform-*d*) δ 7.36 – 7.26 (m, 5H), 5.70 (s, 1H), 4.44 (d, *J* = 5.65 Hz, 2H), 2.03 (s, 3H).

^{13}C NMR (126 MHz, Chloroform-*d*) δ 169.9, 138.4, 128.9, 128.0, 127.7, 44.0, 23.5.
NMR data was consistent with literature values.[15]

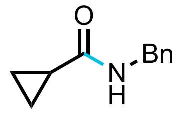

17

Made according to General Procedure A with TBA[$FeBr_4$] at 500 nm. Purified by column chromatography with 2:1 hexanes/EtOAc. Isolated as a white solid (25.5 mg, 73%).

^{1}H NMR (400 MHz, Chloroform-*d*) δ 7.38 – 7.31 (m, 2H), 7.31 – 7.24 (m, 3H), 6.00 (s, 1H), 4.45 (d, *J* = 5.65 Hz, 2H), 1.36 (tt, *J* = 8.09, 4.54 Hz, 1H), 1.00 (dd, *J* = 4.47, 2.80 Hz, 2H), 0.74 (dd, *J* = 7.69, 3.19 Hz, 2H).

^{13}C NMR (101 MHz, Chloroform-*d*) δ 173.6, 138.6, 128.8, 128.0, 127.6, 44.0, 14.9, 7.3.
NMR data was consistent with literature values.[16]

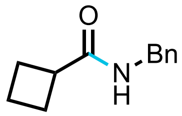

18

Made according to General Procedure A with TBA[$FeBr_4$] at 500 nm. Purified by column chromatography with 4:1 hexanes/EtOAc. Isolated as a white solid (10.3 mg, 27%).

^{1}H NMR (400 MHz, Chloroform-*d*) δ 7.36 – 7.30 (m, 2H), 7.28 (dd, *J* = 7.19, 4.58 Hz, 3H), 5.62 (s, 1H), 4.44 (d, *J* = 5.70 Hz, 2H), 3.02 (pd, *J* = 8.55, 0.91 Hz, 1H), 2.31 (pd, *J* = 9.09, 2.39 Hz, 2H), 2.21 – 2.10 (m, 2H), 2.05 – 1.82 (m, 2H).

^{13}C NMR (101 MHz, Chloroform-*d*) δ 174.9, 138.6, 128.9, 128.0, 127.6, 43.7, 40.1, 25.5, 18.3.
NMR data was consistent with literature values.[16]

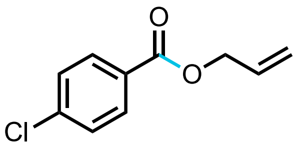

19

Made according to General Procedure C with TBA[$FeBr_4$] at 500 nm. Purified by column chromatography with 4:1 hexanes/EtOAc. Isolated as a clear to pale yellow liquid (13.9 mg, 35%).

^{1}H NMR (400 MHz, Chloroform-*d*) δ 8.00 (d, *J* = 8.57 Hz, 2H), 7.42 (d, *J* = 8.60 Hz, 2H), 6.09 – 5.97 (m, 1H), 5.41 (dq, *J* = 17.13, 1.54 Hz, 1H), 5.30 (dq, *J* = 10.34, 1.28 Hz, 1H), 4.82 (dt, *J* = 5.69, 1.43 Hz, 2H).

^{13}C NMR (101 MHz, Chloroform-*d*) δ 165.6, 139.6, 132.2, 131.2, 128.9, 128.8, 118.7, 65.9.
NMR data was consistent with literature values.[17]

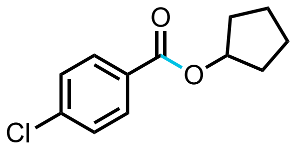

20

Made according to General Procedure C with TBA[$FeBr_4$] at 500 nm. Purified by column chromatography with 2:1 hexanes/EtOAc. Isolated as a pale yellow oil (59.9 mg, 45%) with DMAP and DIPEA and (23.2 mg, 52%) with DMAP and pyridine.

^{1}H NMR (400 MHz, Chloroform-*d*) δ 7.94 (d, *J* = 8.56 Hz, 2H), 7.39 (d, *J* = 8.59 Hz, 2H), 5.39 (tt, *J* = 6.07, 2.68 Hz, 1H), 2.03 – 1.89 (m, 2H), 1.89 – 1.72 (m, 4H), 1.66 (dhept, *J* = 7.28, 4.55, 3.18 Hz, 2H).

^{13}C NMR (101 MHz, Chloroform-*d*) δ 165.6, 139.2, 131.0, 129.5, 128.7, 78.2, 32.9, 23.9.

IR (ATR, neat, cm^{-1}): 2963, 2872, 1711, 1593, 1487, 1320, 1275, 1120, 850

GCMS (EI, QTOF): Calcd for $C_{12}H_{13}ClO_2$ (M^+) = 224.0599, found 224.0596.

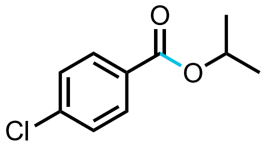

21

Made according to General Procedure C with TBA[$FeBr_4$] at 500 nm. Purified by column chromatography with 4:1 hexanes/EtOAc. Isolated as a clear to pale yellow liquid (27.3 mg, 34%).

^{1}H NMR (400 MHz, Chloroform-*d*) δ 7.97 (d, *J* = 8.55 Hz, 2H), 7.40 (d, *J* = 8.58 Hz, 2H), 5.24 (p, *J* = 6.25 Hz, 1H), 1.36 (d, *J* = 6.25 Hz, 6H).

^{13}C NMR (101 MHz, Chloroform-*d*) δ 165.4, 139.2, 131.1, 129.5, 128.7, 68.9, 22.1.
NMR data was consistent with literature values.[18]

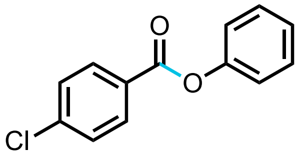

22

Made according to General Procedure C with TBA[$FeBr_4$] at 500 nm. Purified by column chromatography with 4:1 hexanes/EtOAc. Isolated as a white solid (20.8 mg, 45%) with triethylamine and as an off-white solid (34.6 mg, 75%) with pyridine.

^{1}H NMR (400 MHz, Chloroform-*d*) δ 8.18 – 8.12 (m, 2H), 7.52 – 7.47 (m, 2H), 7.47 – 7.40 (m, 2H), 7.29 (d, *J* = 7.42 Hz, 1H), 7.21 (dd, *J* = 8.64, 1.14 Hz, 2H).

^{13}C NMR (101 MHz, Chloroform-*d*) δ 164.5, 150.9, 140.3, 131.7, 129.7, 129.1, 128.2, 126.2, 121.8.
NMR data was consistent with literature values.[19]

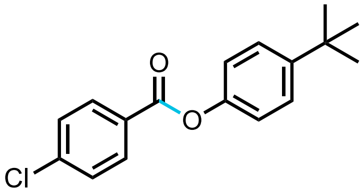

23

Made according to General Procedure C with TBA[$FeBr_4$] at 500 nm. Purified by column chromatography with 4:1 hexanes/EtOAc. Isolated as a white solid (28.0 mg, 49%).

^{1}H NMR (400 MHz, Chloroform-*d*) δ 8.14 (d, *J* = 8.56 Hz, 2H), 7.49 (d, *J* = 8.57 Hz, 2H), 7.44 (d, *J* = 8.72 Hz, 2H), 7.13 (d, *J* = 8.71 Hz, 2H), 1.35 (s, 9H).

^{13}C NMR (101 MHz, Chloroform-*d*) δ 164.6, 149.0, 148.5, 140.2, 131.7, 129.1, 128.3, 126.6, 121.0, 34.7, 31.6.
NMR data was consistent with literature values.[20]

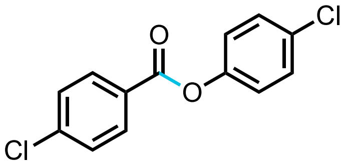

24

Made according to General Procedure C with TBA[$FeBr_4$] at 500 nm. Purified by column chromatography with 4:1 hexanes/EtOAc. Isolated as a white solid (27.5 mg, 52%).

^{1}H NMR (400 MHz, Chloroform-*d*) δ 8.12 (d, *J* = 8.60 Hz, 2H), 7.50 (d, *J* = 8.59 Hz, 2H), 7.40 (d, *J* = 8.83 Hz, 2H), 7.16 (d, *J* = 8.83 Hz, 2H).

^{13}C NMR (101 MHz, Chloroform-*d*) δ 164.3, 149.4, 140.5, 131.7, 131.6, 129.8, 129.2, 127.8, 123.2.
NMR data was consistent with literature values.[20]

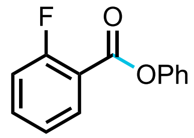

25

Made according to General Procedure C with TBA[$FeBr_4$] at 500 nm. Purified by column chromatography with 10:1 hexanes/EtOAc. Isolated as a yellow solid (23.5 mg, 54%).

^{1}H NMR (500 MHz, Chloroform-*d*) δ 8.11 (td, *J* = 7.54, 1.86 Hz, 1H), 7.64 – 7.57 (m, 1H), 7.47 – 7.41 (m, 2H), 7.31 – 7.18 (m, 5H).

^{13}C NMR (126 MHz, Chloroform-*d*) δ 162.9 (d, *J* = 4.13 Hz), 162.5 (d, *J* = 261.35 Hz), 150.8, 135.3 (d, *J* = 9.10 Hz), 132.7, 129.6, 126.2, 124.3 (d, *J* = 3.91 Hz), 121.8, 118.2 (d, *J* = 9.28 Hz), 117.4 (d, *J* = 22.18 Hz).
NMR data was consistent with literature values.[21,22]

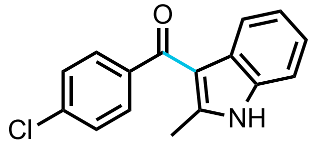

26

Made according to General Procedure D with TBA[$FeBr_4$] at 500 nm. Purified by column chromatography with 4:1 hexanes/EtOAc. Isolated as a brown solid (26.9 mg, 50%).

^{1}H NMR (400 MHz, Chloroform-*d*) δ 8.65 (s, 1H), 7.75 – 7.70 (m, 2H), 7.46 – 7.41 (m, 2H), 7.37 – 7.30 (m, 2H), 7.18 (ddd, *J* = 8.20, 7.13, 1.22 Hz, 1H), 7.09 (ddd, *J* = 8.21, 7.15, 1.16 Hz, 1H), 2.57 (s, 3H).

^{13}C NMR (101 MHz, Chloroform-*d*) δ 191.8, 143.9, 139.6, 137.9, 134.8, 130.6, 128.7, 127.5, 122.7, 121.8, 121.0, 113.9, 110.9, 14.7.
NMR data was consistent with literature values.[23,24]

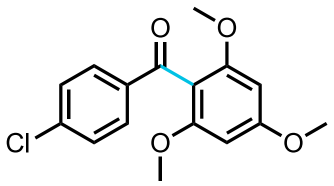

27

Made according to General Procedure D with TBA[$FeBr_4$] at 500 nm. Purified by column chromatography with 4:1 hexanes/EtOAc. Isolated as a white solid (23.7 mg, 39%).

^{1}H NMR (400 MHz, Chloroform-*d*) δ 7.77 (d, *J* = 8.30 Hz, 2H), 7.37 (d, *J* = 8.32 Hz, 2H), 6.16 (s, 2H), 3.86 (s, 3H), 3.69 (s, 6H).

^{13}C NMR (101 MHz, Chloroform-*d*) δ 193.8, 162.8, 158.9, 139.4, 136.8, 131.0, 128.7, 110.5, 90.8, 55.9.
NMR data was consistent with literature values.[25]

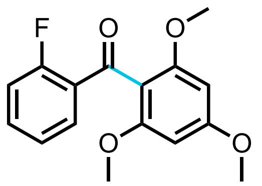

28

Made according to General Procedure D with TBA[$FeBr_4$] at 500 nm. Purified by column chromatography with 4:1 hexanes/EtOAc. Isolated as a white solid (32.3 mg, 59% yield).

^{1}H NMR (500 MHz, Chloroform-*d*) δ 7.77 (td, *J* = 7.68, 1.85 Hz, 1H), 7.49 – 7.42 (m, 1H), 7.17 (td, *J* = 7.58, 1.10 Hz, 1H), 7.03 (ddd, *J* = 11.08, 8.29, 1.15 Hz, 1H), 6.14 (s, 2H), 3.85 (s, 3H), 3.69 (s, 6H).

^{13}C NMR (126 MHz, Chloroform-*d*) δ 190.9, 162.9, 161.8 (d, *J* = 257.89 Hz), 159.2, 134.0 (d, *J* = 8.85 Hz), 131.6 (d, *J* = 1.85 Hz), 128.1 (d, *J* = 9.53 Hz), 124.0 (d, *J* = 3.83 Hz), 116.5 (d, *J* = 22.56 Hz), 113.2, 90.9, 56.0, 55.6.
NMR data was consistent with literature values.[26]

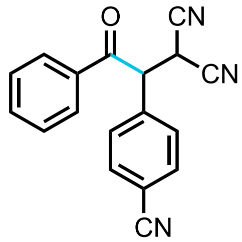

33

Made following standard procedure published by Martin and colleagues using catalyst **1d**.[2] Isolated as a beige solid (5.7 mg, 14%).

^{1}H NMR (400 MHz, Chloroform-*d*) δ 7.88 – 7.83 (m, 2H), 7.77 – 7.71 (m, 2H), 7.63 – 7.57 (m, 1H), 7.53 – 7.48 (m, 2H), 7.48 – 7.41 (m, 2H), 5.17 (d, *J* = 8.07 Hz, 1H), 4.57 (d, *J* = 8.06 Hz, 1H).

^{13}C NMR (101 MHz, Chloroform-*d*) δ 192.2, 137.0, 135.2, 133.9, 133.5, 129.7, 129.4, 129.4, 117.7, 114.4, 111.6, 111.1, 54.4, 26.6.
NMR data was consistent with literature values.[27]

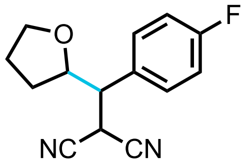

36

Made following standard procedure published by Martin and colleagues using catalyst **1d**.[2] NMR data was consistent with literature values.[2]

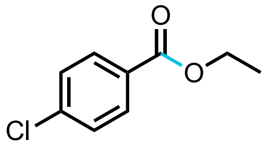

S1

Made according to General Procedure C with TBA[$FeBr_4$] at 500 nm. Purified by column chromatography with 4:1 hexanes/EtOAc. Isolated as a clear to pale yellow liquid (9.7 mg, 26%).

^{1}H NMR (400 MHz, Chloroform-*d*) δ 7.98 (d, *J* = 8.60 Hz, 2H), 7.40 (d, *J* = 8.58 Hz, 2H), 4.37 (d, *J* = 7.13 Hz, 2H), 1.39 (t, *J* = 7.13 Hz, 3H).

^{13}C NMR (101 MHz, Chloroform-*d*) δ 165.9, 139.4, 131.1, 129.1, 128.8, 61.4, 14.4.
NMR data was consistent with literature values.[28]

E. References

1. Li, S.; Zhu, B.; Lee, R.; Qiao, B.; Jiang, Z. Visible light-induced selective aerobic oxidative transposition of vinyl halides using a tetrahalogenoferrate(iii) complex catalyst. *Org. Chem. Front.* **2018**, *5*, 380-385.
2. Ludwig, C.T.; Owolabi, I.A.; Evans, L.W.; Smith, G.J.; Ramos, A.; Shepherd, J.J.; Martin, D.B.C. 25-01-27, Wavelength-Selective Reactivity of Iron(III) Halide Salts in Photocatalytic C–H Functionalization. doi:10.26434/chemrxiv-2024-nbcsr-v2.
3. Nathanael, J.G.; White, J.M.; Richter, A.; Nuske, M.R.; Wille, U. Oxidative damage of proline residues by nitrate radicals ($NO_3^•$): a kinetic and product study. *Org. Biomol. Chem.* **2020**, *18*, 6949-6957.
4. Pitre, S.P.; McTiernan, S.D.; Vine, W.; DiPucchio, R.; Grenier, M.; Scaiano, J.C. Visible-Light Actinometry and Intermittent Illumination as Convenient Tools to Study $Ru(bpy)_3Cl_2$ Mediated Photoredox Transformations. *Sci. Rep.* **2015**, *5*, 16397. DOI: https://doi.org/10.1038/srep16397.
5. Lagueux-Tremblay, P.-L.; Tam, K.M.; Jiang, M.; Arndtsen, B.A. Electrifying Redox-Neutral Palladium-Catalyzed Carbonylations: Multielectron Transfer as a Catalyst Driving Force. *J. Am. Chem. Soc.* **2025**, *147*, 17239-17250.
6. Cao, Z.; Sheng, D.; Zhang, Z.; Ren, H.; Liu, Y.; Wu, S.; Zhang, J.; Han, X. Copper-Promoted Oxidative Amidation of Imines: A Facile Route to Amides. *Eur. J. Org. Chem.* **2024**, *27*, e202400315.
7. Philo, J.E.; Caudle, J.D.; Moussa, R.N.; Kampmeyer, P.M.; Hasin, T.R.; Seo, D.K.; Sheaff, R.J.; Lamar, A.A. Synthesis and Biological Evaluation of a Library of Sulfonamide Analogs of Memantine to Target Glioblastoma. *ChemMedChem*. **2023**, *18*, e202300134.
8. Soliman, E.; Baek, H.; Mase, N.; Yamada, Y.M.A. Continuous-Flow Ritter Reaction for Sustainable Amide Synthesis Using a Recyclable m-Phenolsulfonic Acid-Formaldehyde Resin Catalyst. *J. Org. Chem.* **2025**, *90*, 1447−1454.
9. Swetha, S.; Senadi, G.C. An Umpolung Route to Amides from α-Aminonitriles under Metal-Free Conditions. *Adv. Synth. Catal.* **2022**, *364*, 2872-2882.
10. Roy, K.; Saha, A.; Saha, B.; Banerjee, S.; Mukhopadhyay, C.D.; Sahu, S.K.; Adak, L. Reusable Iron-Copper Catalyzed Cross-Coupling of Primary Amides with Aryl and Alkyl Halides: Access to N-Arylamides as Potential Antibacterial and Anticancer Agents. *Chem. Eur. J.* **2025**, *31*, e202403649.
11. Tolba, A.H.; Krupička, M.; Chudoba, J.; Cibulka, R. Amide Bond Formation via Aerobic Photooxidative Coupling of Aldehydes with Amines Catalyzed by a Riboflavin Derivative. *Org. Lett.* **2021**, *23*, 17, 6825-6830.
12. Cao, H.; Shi, Y.; Yan, P.; Bie, F.; Ma, J. Microwave-assisted direct amidation of thioesters: a green approach. *Org. Biomol. Chem.* **2025**, *23*, 7793-7800.

13. Spieß, P.; Brześkiewicz, J.; Meyrelles, R.; Just, D.; Maulide, N. Deprotective Functionalization: A Direct Conversion of Nms-Amides to Carboxamides Using Carboxylic Acids. *Angew. Chem. Int. Ed.* **2024**, *63*, e202318304.
14. Kuriakose, S.; Ravindra, S.; Kandasamy, J. A Mild and Efficient Synthesis of Functionalized 2-Fluorobenzamides from 1,2,3-Benzotriazinones *via* Denitrogenative Fluorination Reactions under Thermal/Visible Light Conditions. *Eur. J. Org. Chem.* **2025**, *28*, e202500375.
15. Hamann, H.J.; Snyder, M.J.; Singh, A.G.; Alawaed, A.A.; Ramachandran, P.V. Rapid, Room-Temperature Amidation via Tandem Titanium Amido Complex and Titanium Carboxylate Intermediates. *Org. Lett.* **2025**, *27*, 9831-9836.
16. Hilvano, E.G.V.; Liang, M.-C.; Piane, J.J.; Nacsa, E.D. Direct electrochemical synthesis of pentafluorophenyl esters *via* oxyl-radical-promoted nucleophilic aromatic substitution. *Org. Biomol. Chem.* **2025**, *23*, 6373-6385.
17. Prasad, R.; Singh, S.K.; Maity, R.; Ghosh, P. Conversion of aromatic methyl ketones to esters and carboxylic acids using o-phthalaldehyde as an oxidant. *Org. Biomol. Chem.* **2025**, *23*, 1120-1128.
18. Koziakov, D.; von Wangelin, A.J. Metal-free radical aromatic carbonylations mediated by weak bases. *Org. Biomol. Chem.* **2017**, *15*, 6715-6719.
19. Liu, F.; Sohail, A.; Ablajan, K. Metal-Free Oxidative Formation of Aryl Esters by Catalytic Coupling of Acyl and Sulfonyl Chlorides with Arylboronic Acids. *J. Org. Chem.* **2024**, *89*, 27-33.
20. Arde, P.; Ramanjaneyulu, B.T.; Reddy, V.; Saxena, A.; Anand, R.V. N-Heterocyclic carbene catalysed aerobic oxidation of aromatic aldehydes to aryl esters using boronic acids. *Org. Biomol. Chem.* **2012**, *10*, 848-851.
21. Man, Y.; Xu, B. Generation and Radical-Radical Cross-Coupling of Alkenyloxy Radical. *Org. Lett.* **2024**, *26*, 2456-2461.
22. Shi, Y.; Liu, X.; Cao, H.; Bie, F.; Han, Y.; Yan, P.; Szostak, R.; Szostak, M.; Liu, C. Conversion of esters to thioesters under mild conditions. *Org. Biomol. Chem.* **2021**, *19*, 2991-2996.
23. Suzdalev, K.F.; Vyalyh, J.V.; Tkachev, V.V.; Lysenko, E.A.; Burov, O.N.; Lisovin, A.V. Kletskii, M.E.; Kurbatov, S.V. Lithium-Promoted Cycloaddition of Indole-2,3-dienolates and Carbon Disulfide as a One-Pot Route to Thiopyrano[4,3-b]indole-3(5H)-thiones. *J. Org. Chem.* **2021**, *86*, 11698-11707.
24. Jaiswal, A.; Sharma, A.K.; Singh, K.N. Synthesis of 3-acylindoles via copper-mediated oxidative decarbethoxylation of ethyl arylacetates. *Org. Biomol. Chem.* **2020**, *18*, 1623-1628.
25. Gavit, B.R.; Hanania, N.; Eghbarieh, N.; Shioukhi, I.; Masarwa, A. Programmable Strategies for the Conversion of Aldehydes to Unsymmetrical (Deuterated) Diarylmethanes and Diarylketones. *Org. Lett.* **2025**, *27*, 3637-3642.

26. Mzozoyana, V.; van Heerden, F.R. Synthesis of fluorine-containing prenylated benzophenones. Synth. Commun. 2020, 50, 2226-2235.
27. Yang, M.-L.; Dong, C.-L.; Guan, Z.; He, Y.-H. Visible Light-Induced Hydroacylation of Benzylidenemalononitriles with Aroyl Chlorides Using Silane as a Hydrogen Donor. *J. Org. Chem.* **2024**, *89*, 1285-1295.
28. Kong, X.; Chen, Y.; Chen, X.; Lu, Z.-X.; Wang, W.; Ni, S.-F.; Cao, Z.-Y. A Practically Unified Electrochemical Strategy for Ni-Catalyzed Decarboxylative Cross-Coupling of Aryl Trimethylammonium Salts. *Org. Lett.* **2022**, *24*, 2137-2142.

F. NMR Spectra

^{1}H NMR (400.15 MHz) and ^{13}C NMR for Compound **4** in $CDCl_3$:

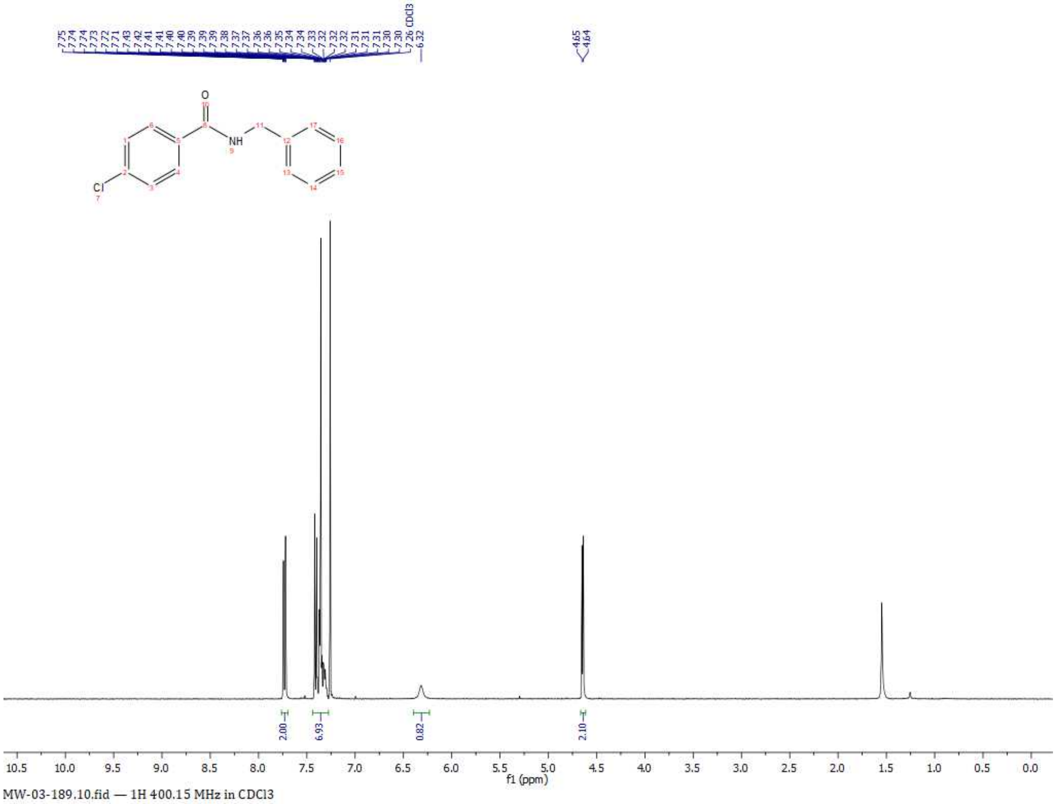

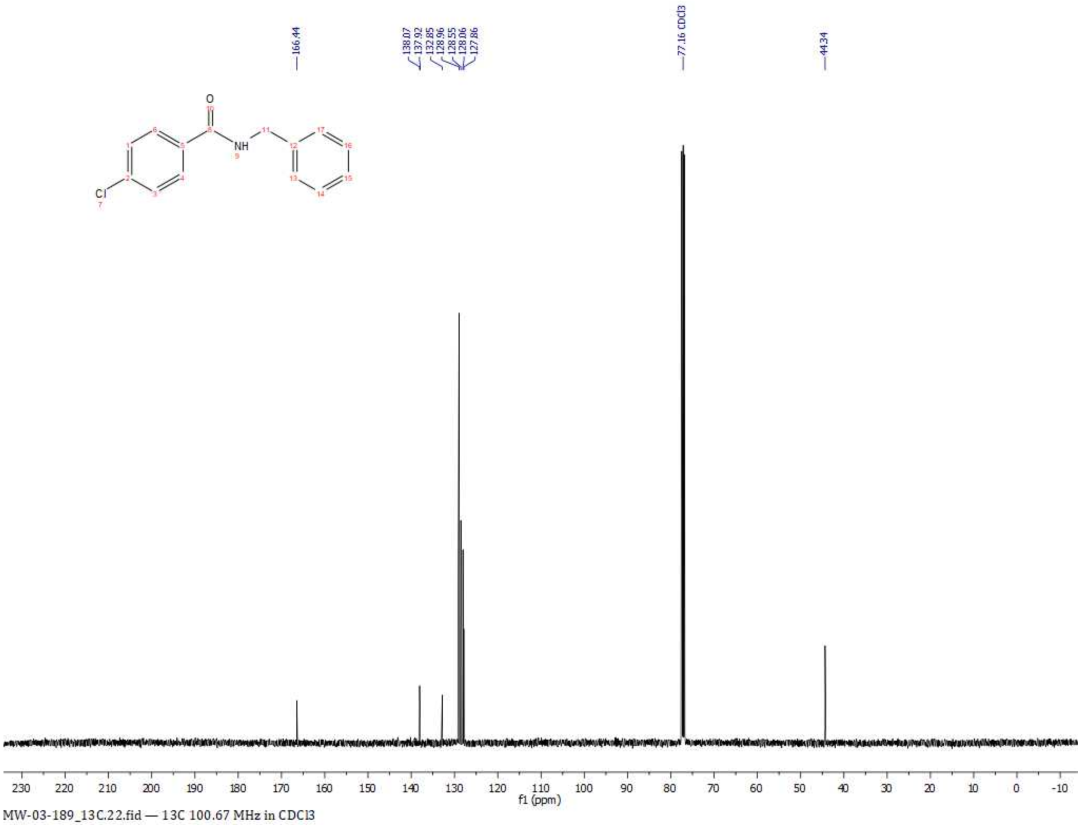

^{1}H NMR (400.15 MHz) and ^{13}C NMR for Compound **5** in $CDCl_3$:

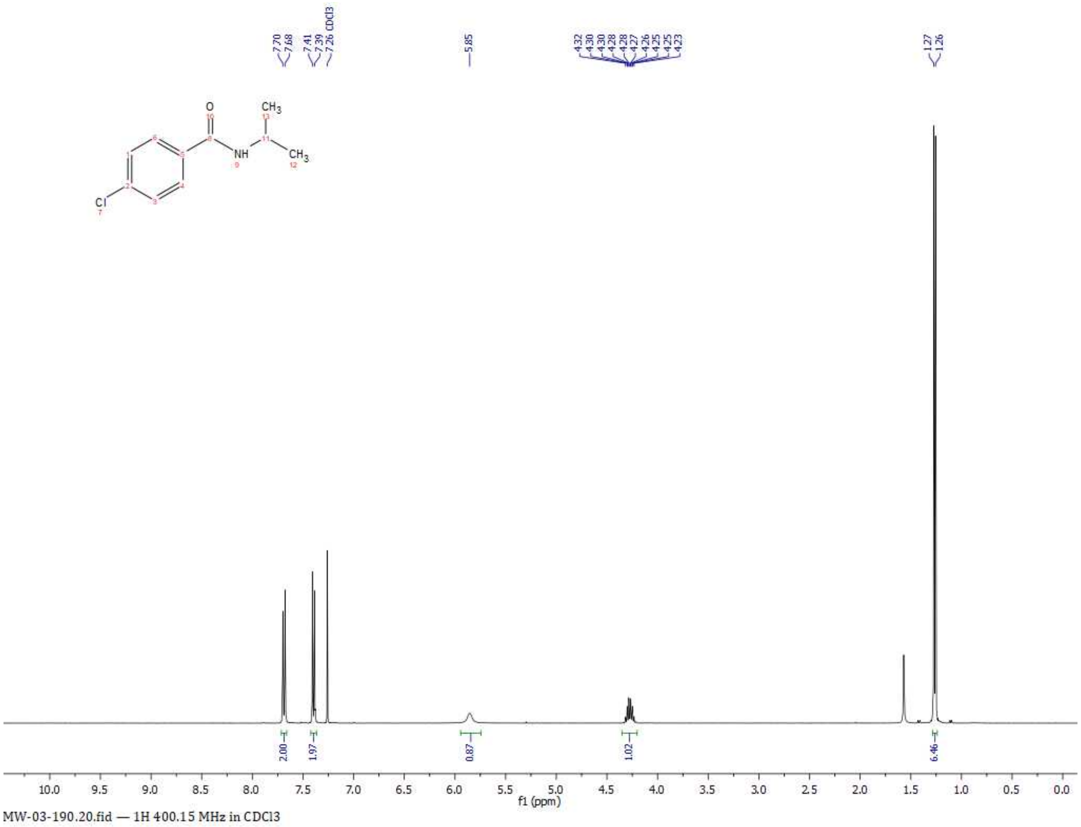

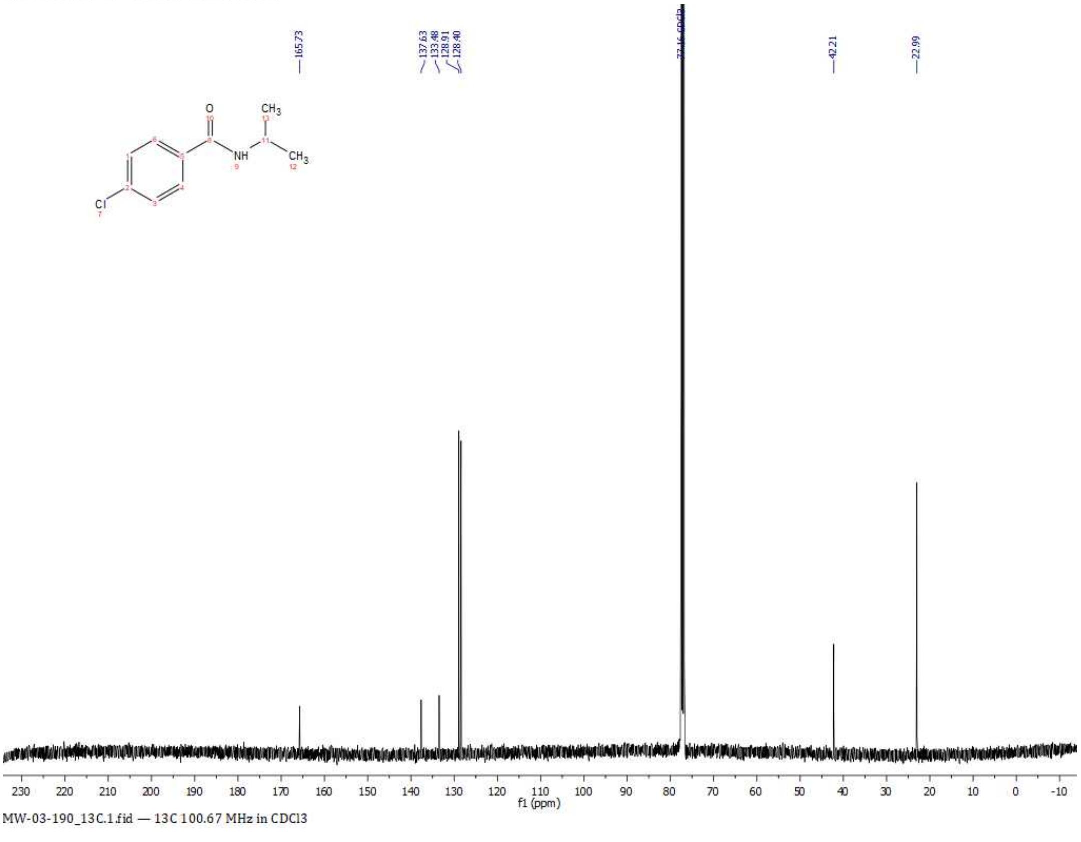

^1H NMR (400.15 MHz) and ^{13}C NMR for Compound **6** in $CDCl_3$:

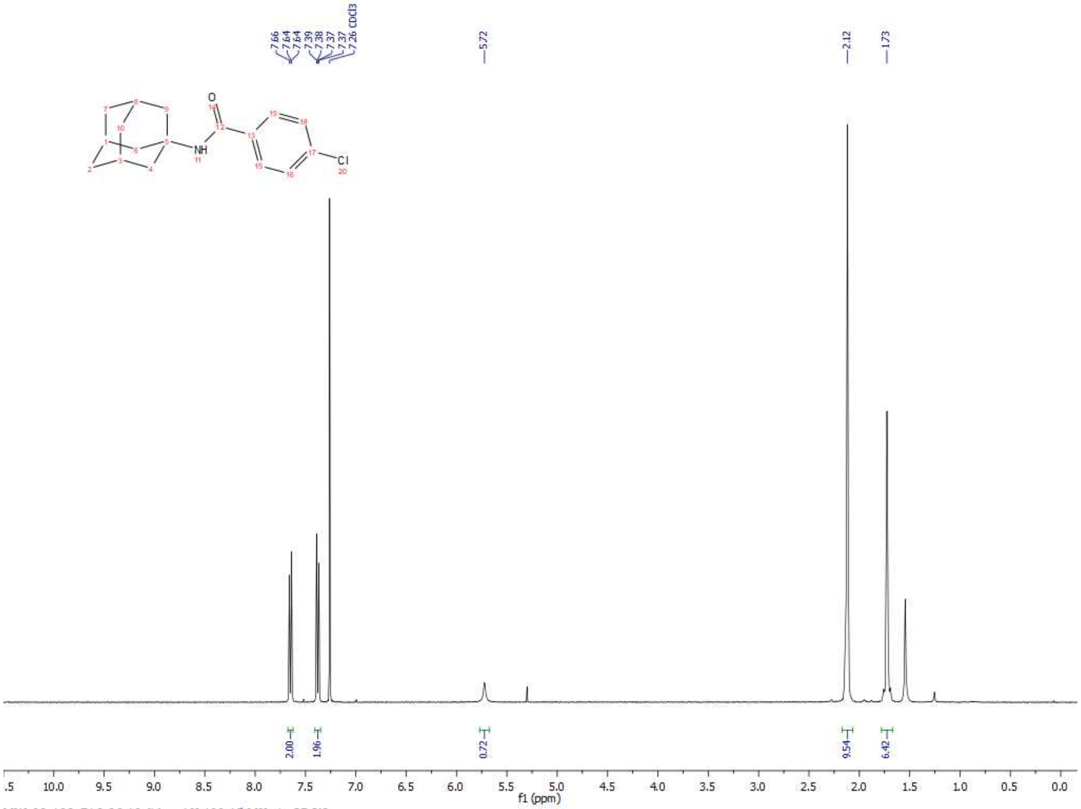

MW-03-193_F13-20.10.fid — 1H 400.15 MHz in CDCl3

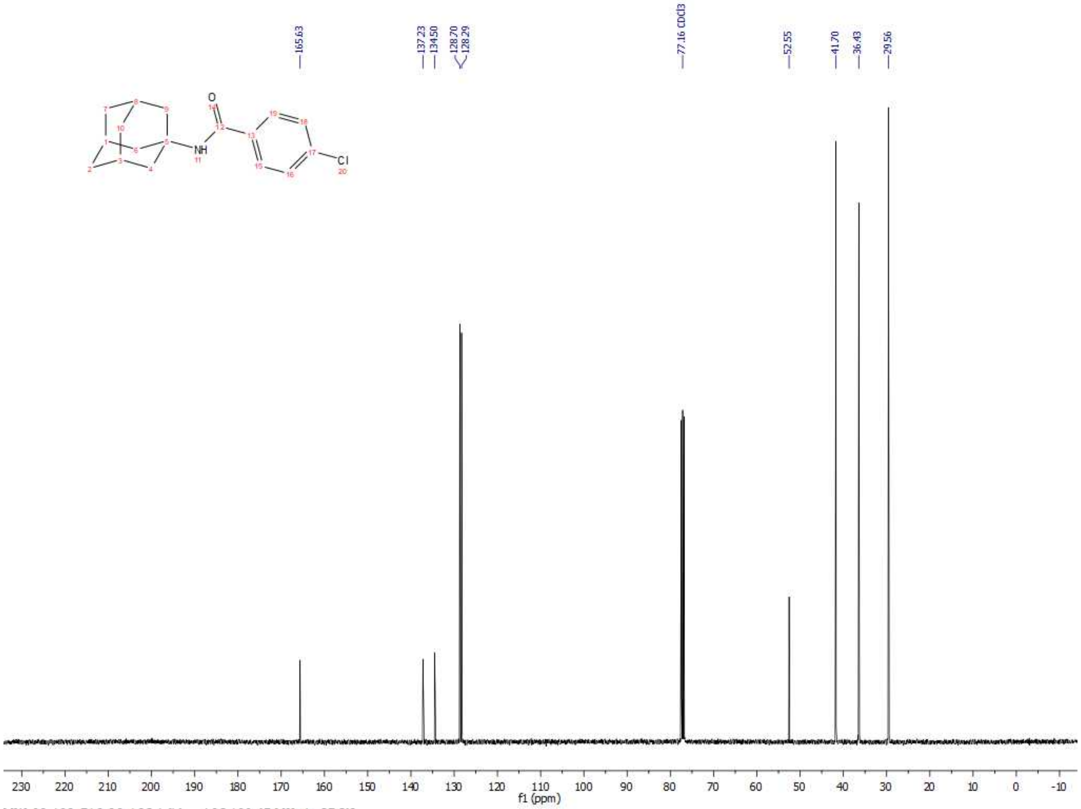

MW-03-193_F13-20_13C.1.fid — 13C 100.67 MHz in CDCl3

^{1}H NMR (400.15 MHz) and ^{13}C NMR for Compound **7** in $CDCl_3$:

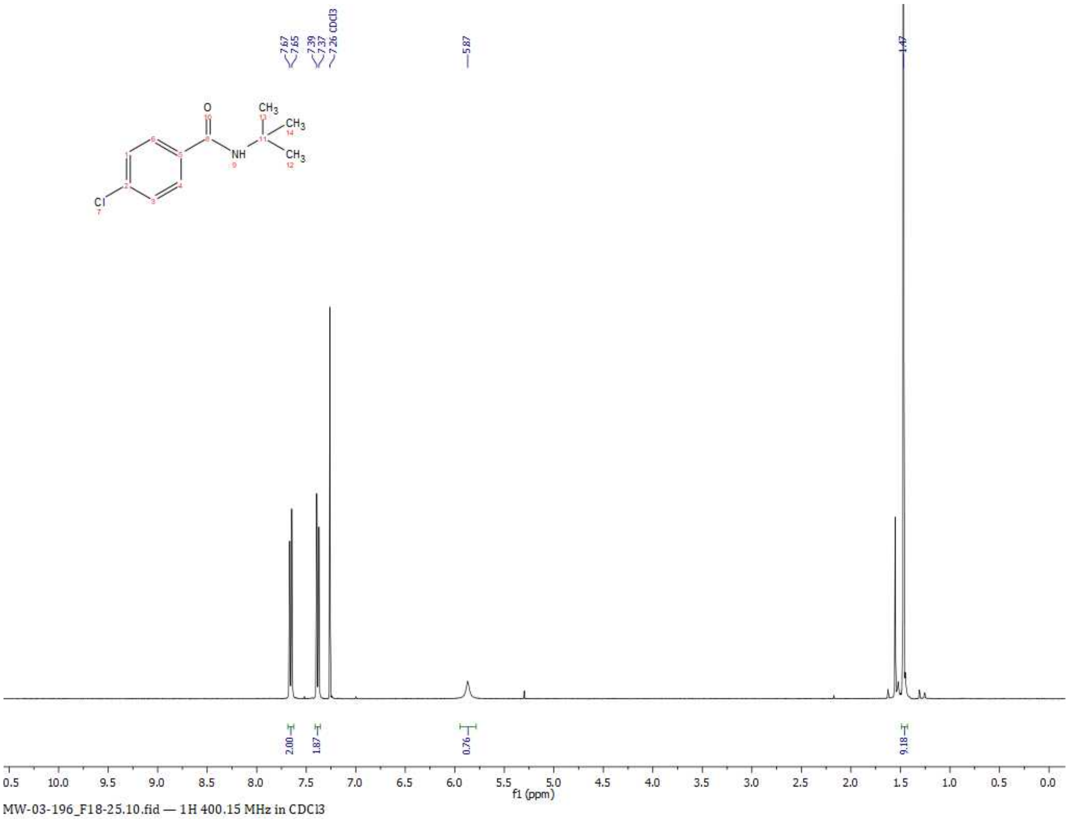

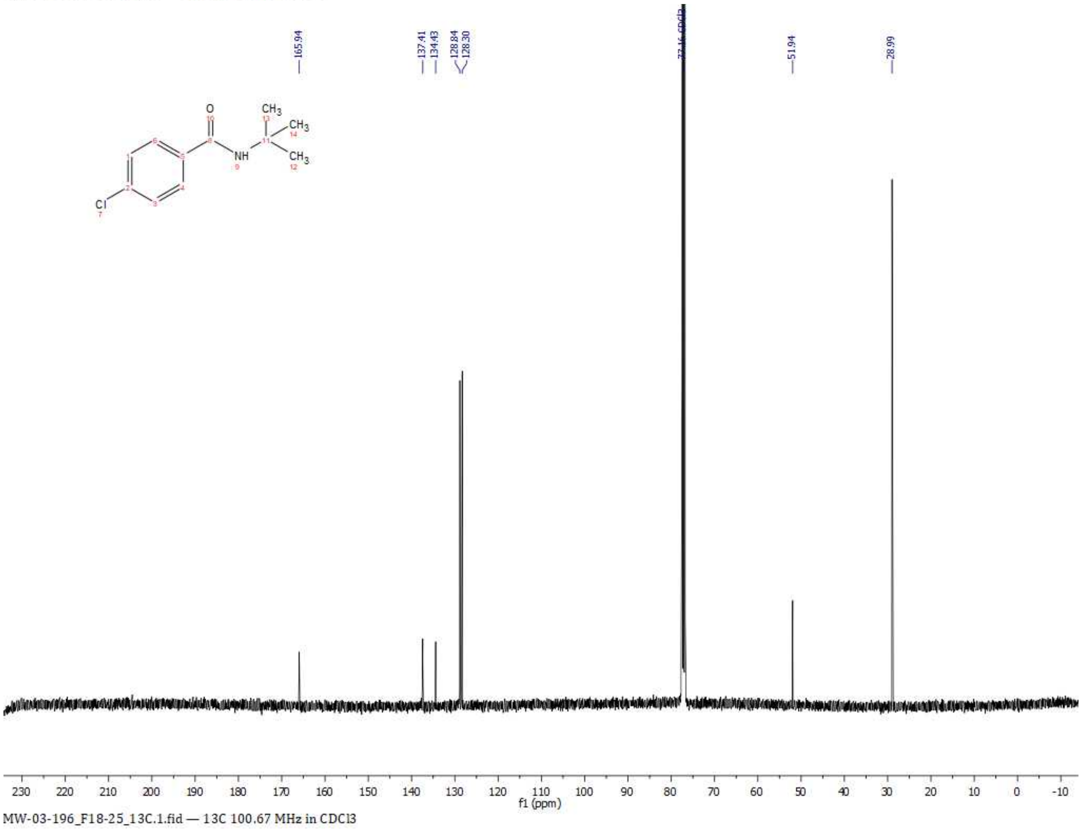

^{1}H NMR (400.15 MHz) and ^{13}C NMR for Compound **8** in $CDCl_3$:

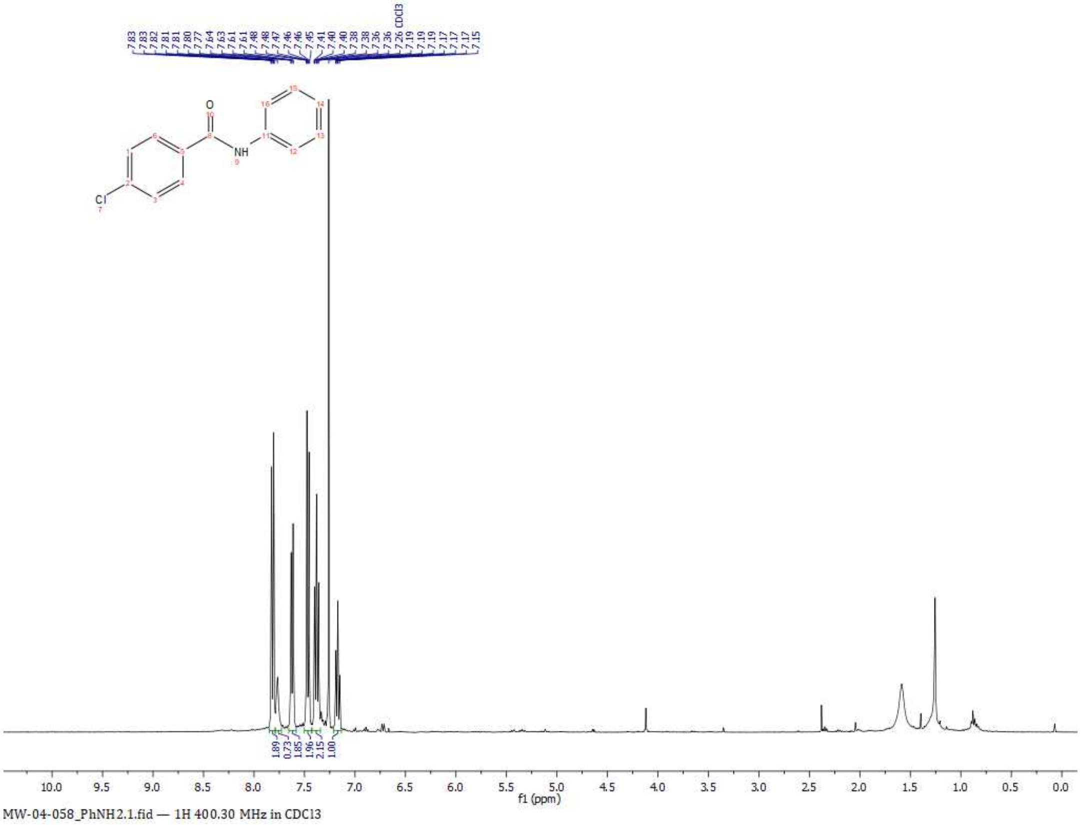

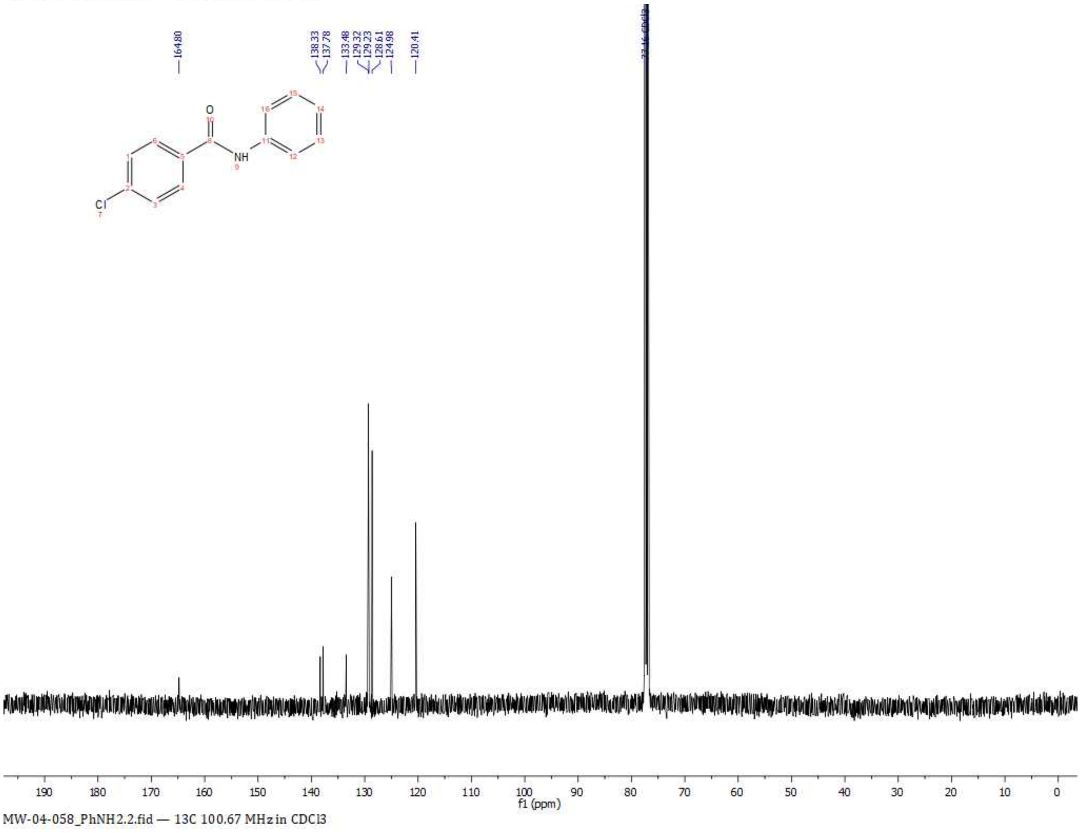

^1H NMR (400.15 MHz) and ^{13}C NMR for Compound **9** in $CDCl_3$:

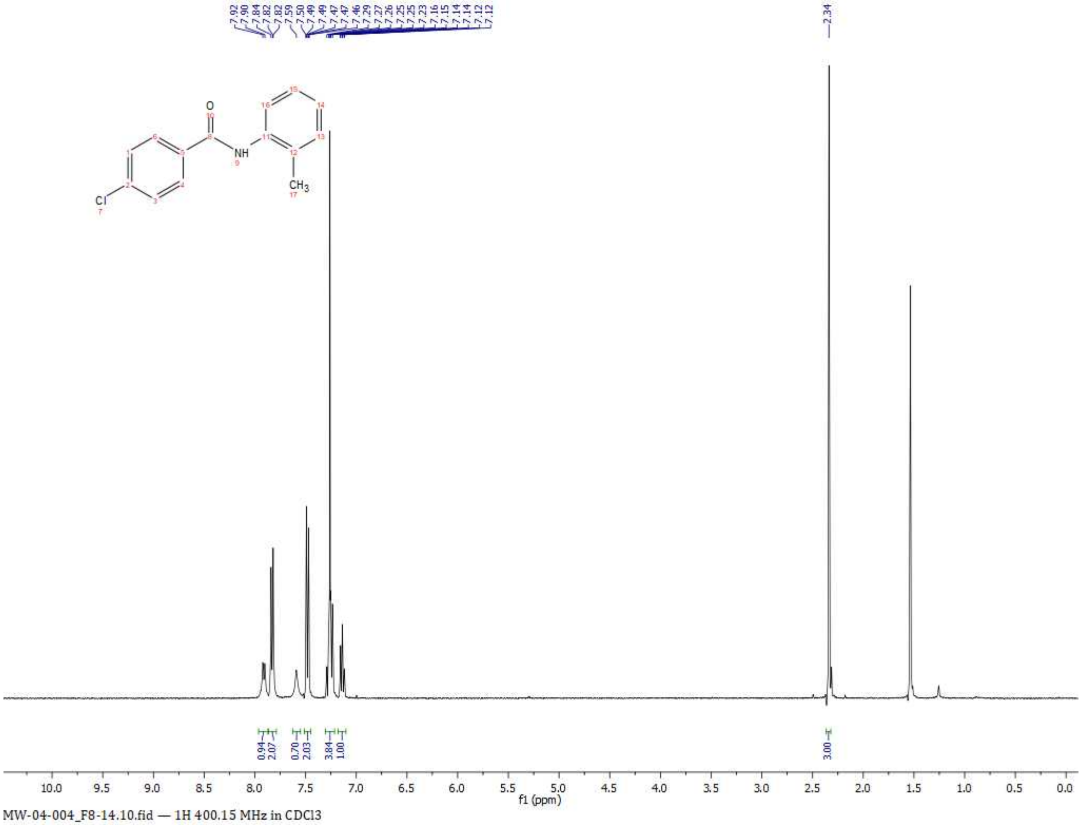

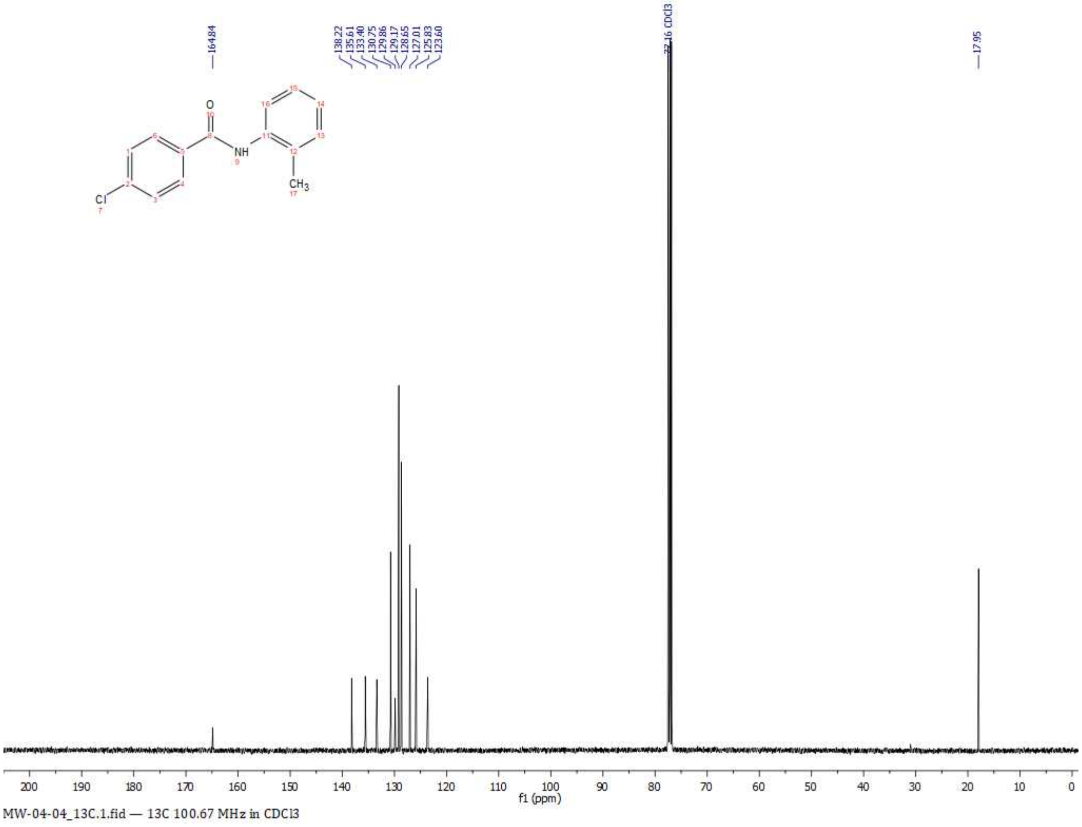

^{1}H NMR (400.15 MHz) and ^{13}C NMR for Compound **10** in $CDCl_3$:

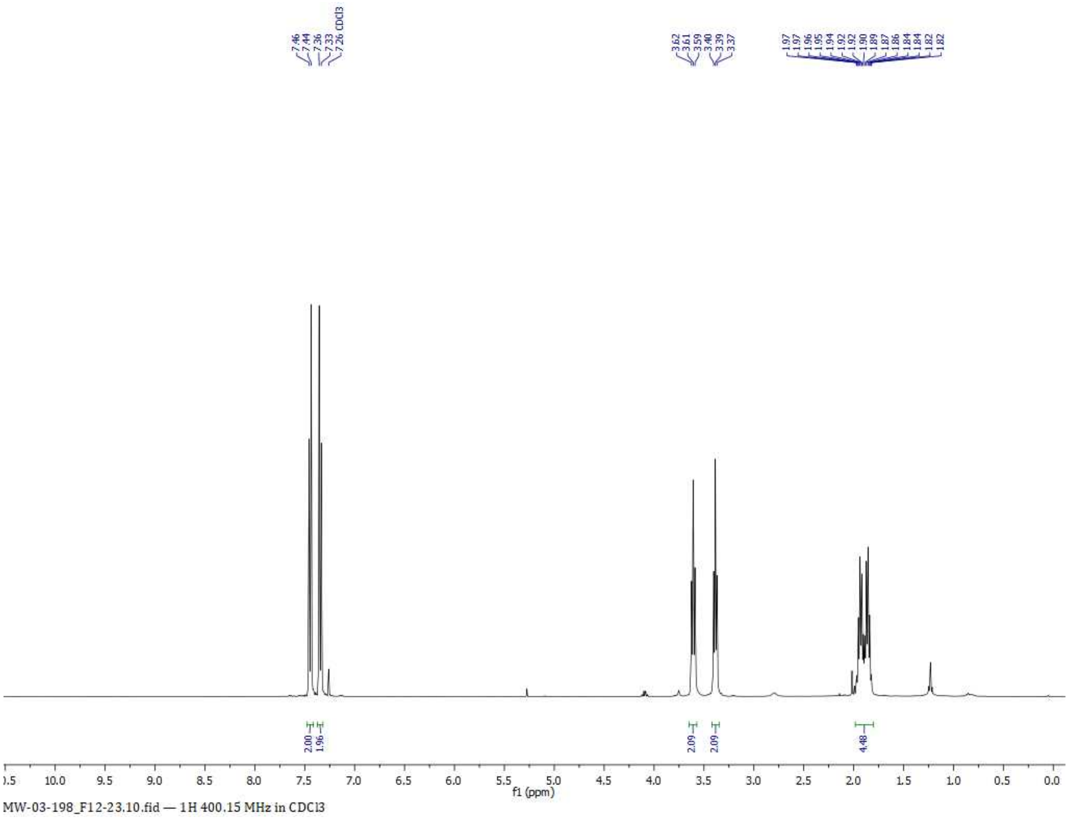

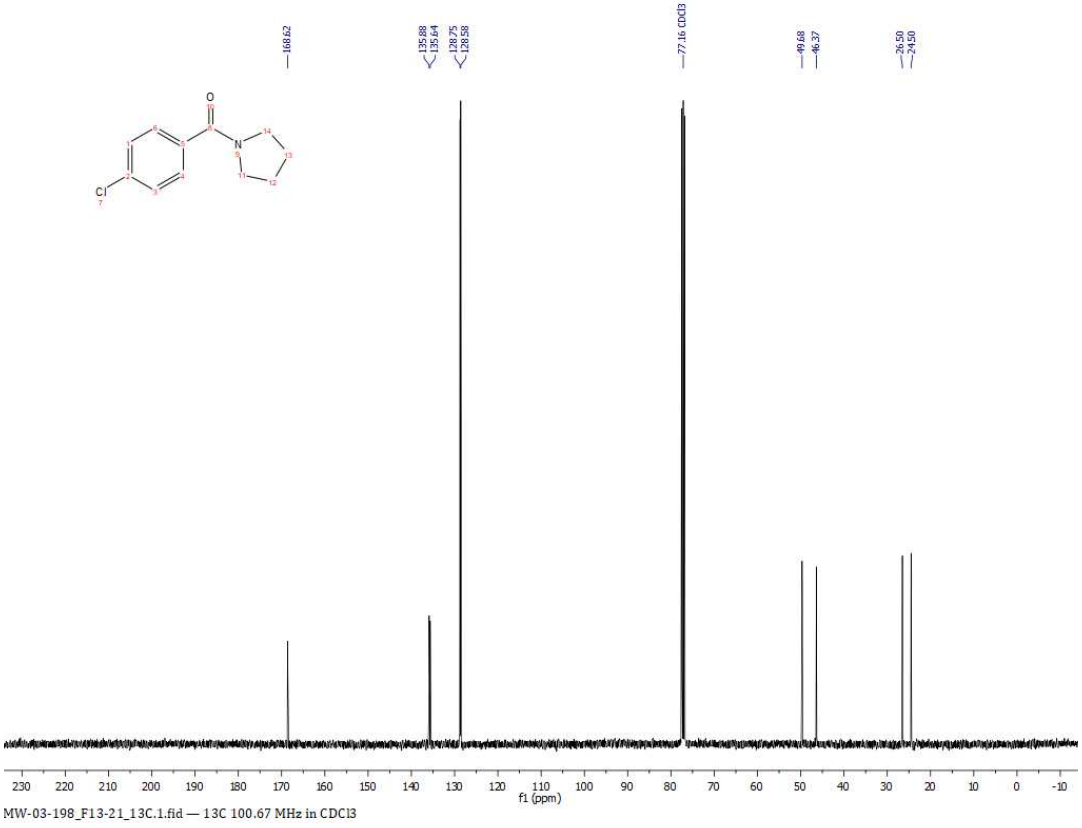

^{1}H NMR (400.15 MHz) and ^{13}C NMR (100.67) for Compound **11** in $CDCl_3$:

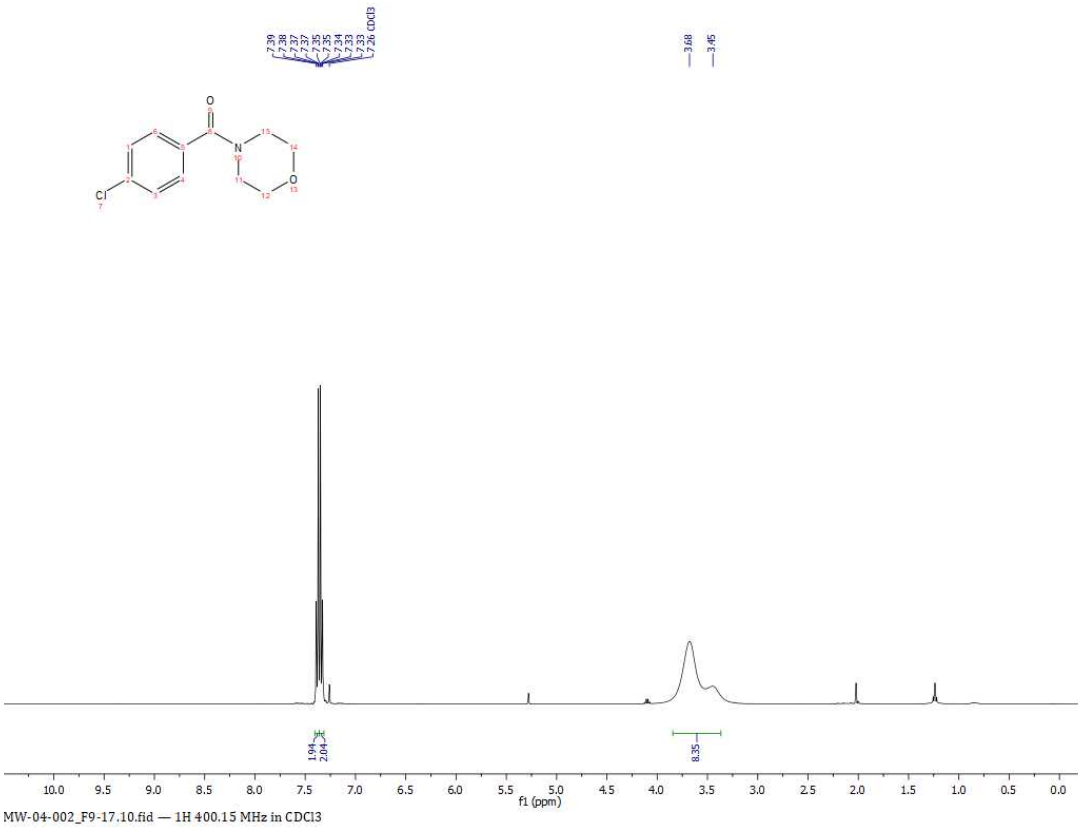

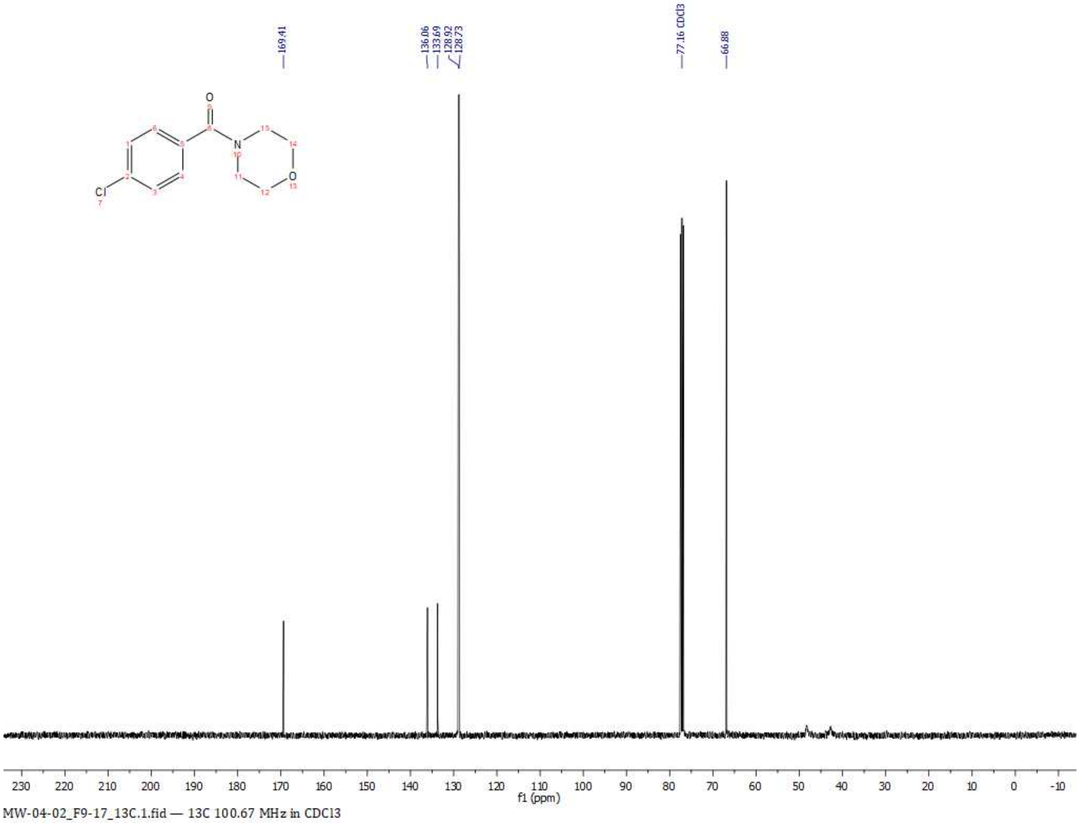

^{1}H NMR (400.15 MHz) and ^{13}C NMR (100.67) for Compound **12** in $CDCl_3$:

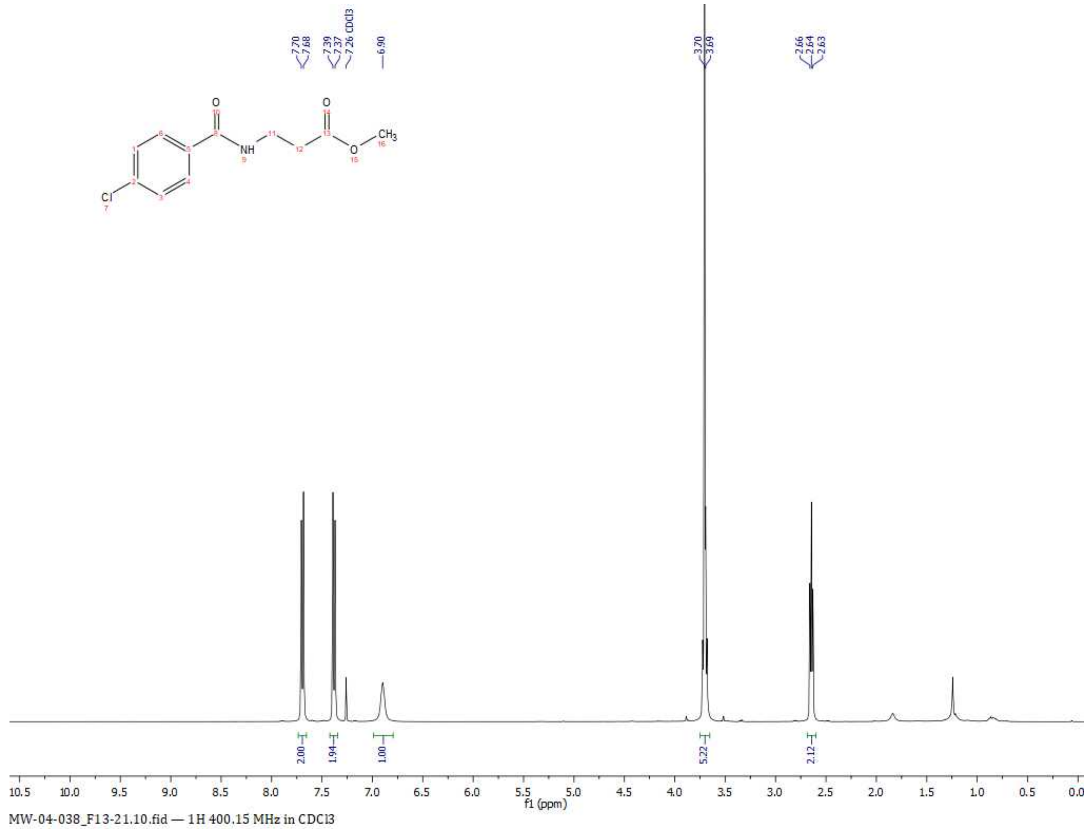

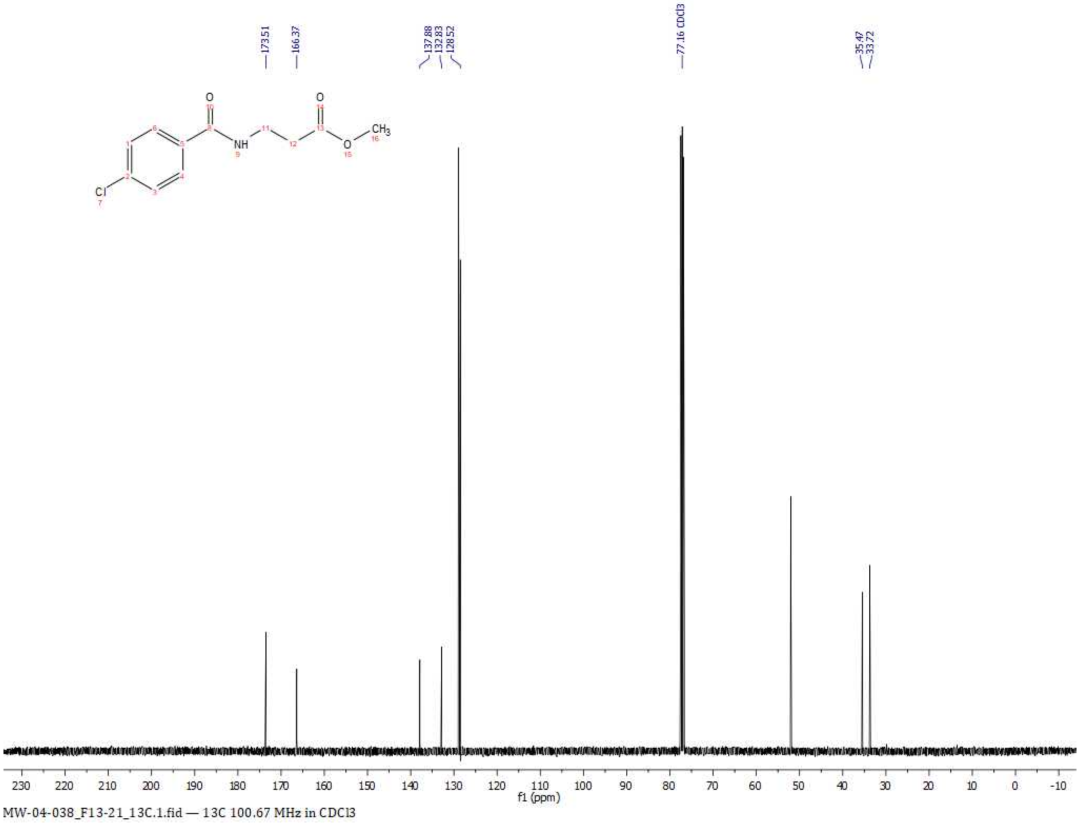

^{1}H NMR (400.15 MHz) and ^{13}C NMR (100.67) for Compound **13** in $CDCl_3$:

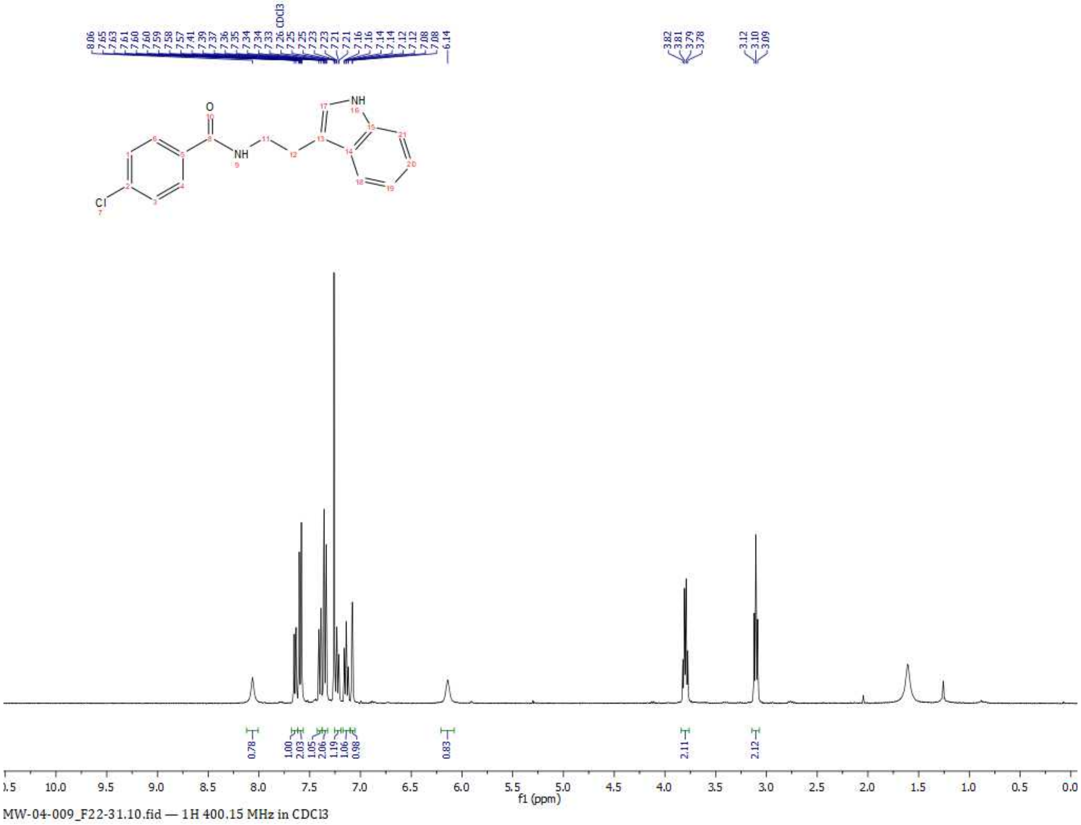

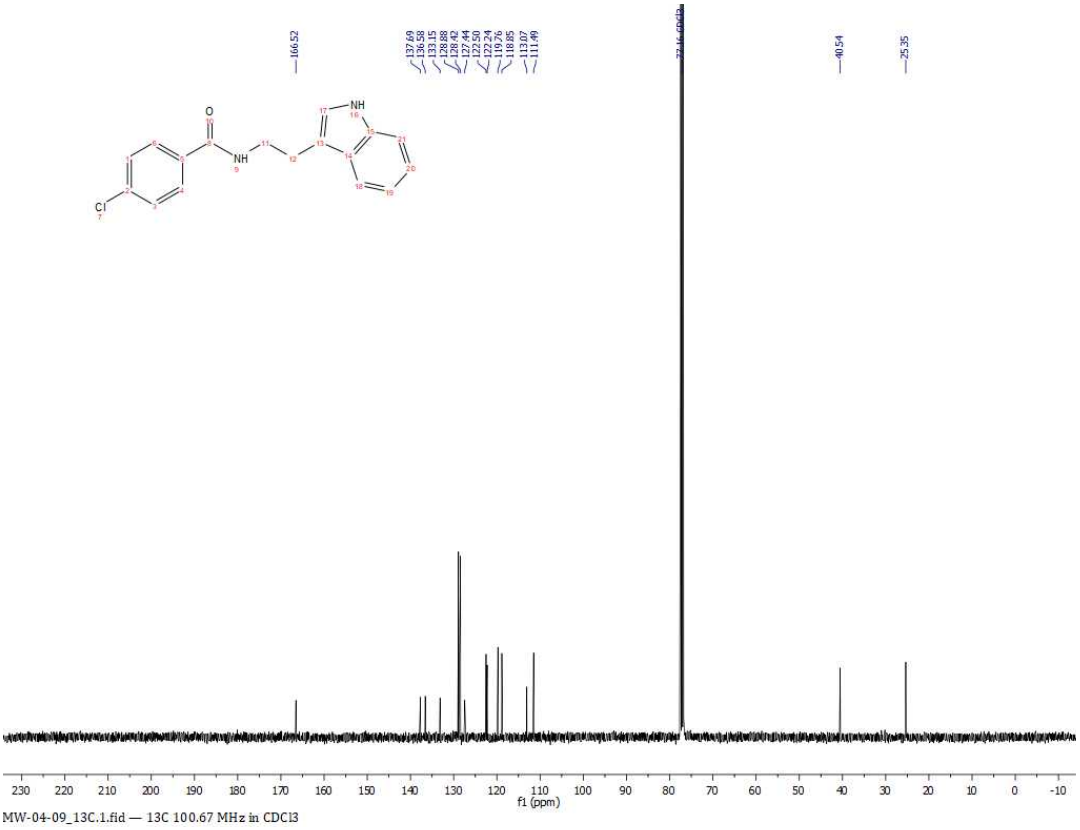

^{1}H NMR (400.15 MHz) and ^{13}C NMR (100.67) for Compound **14** in $CDCl_3$:

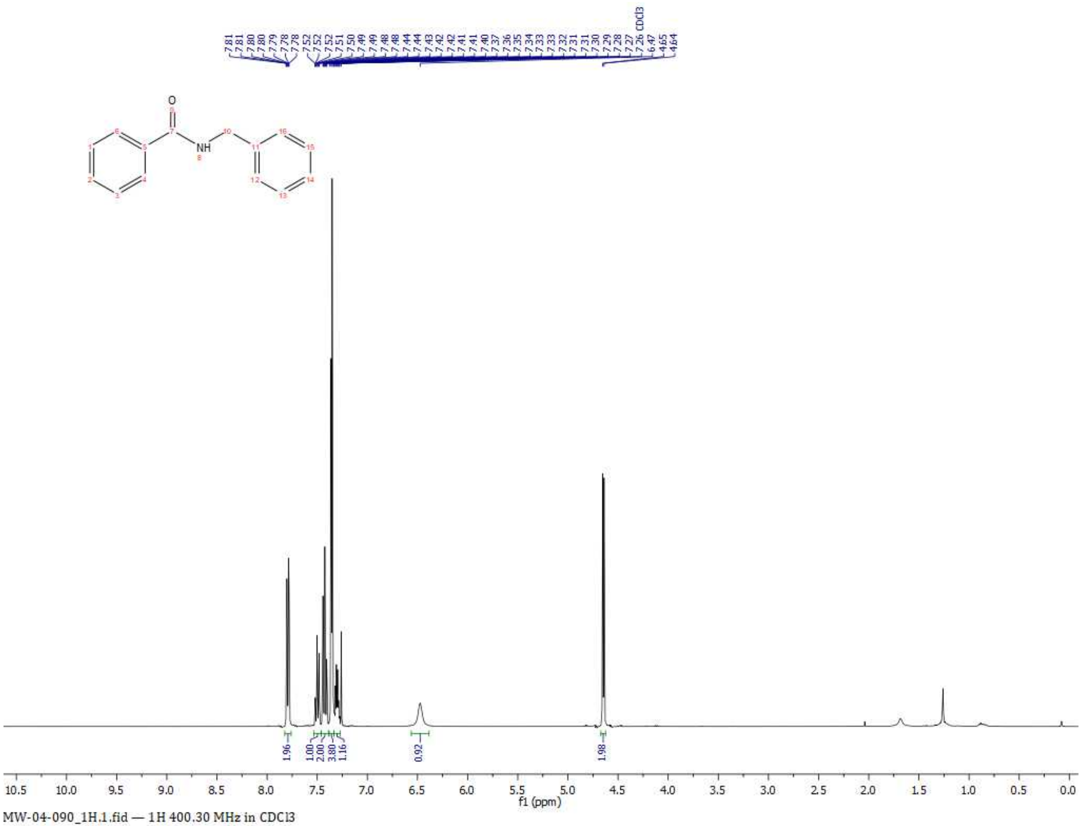

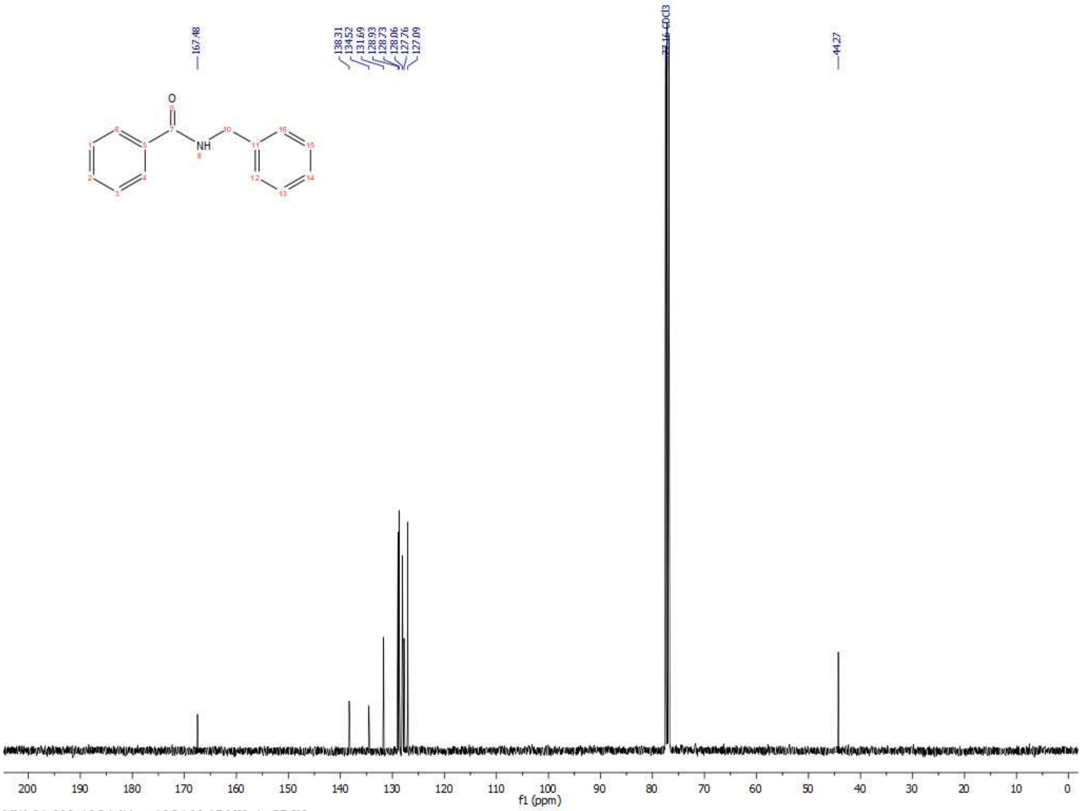

^{1}H NMR (400.15 MHz) and ^{13}C NMR (100.67) for Compound **15** in $CDCl_3$:

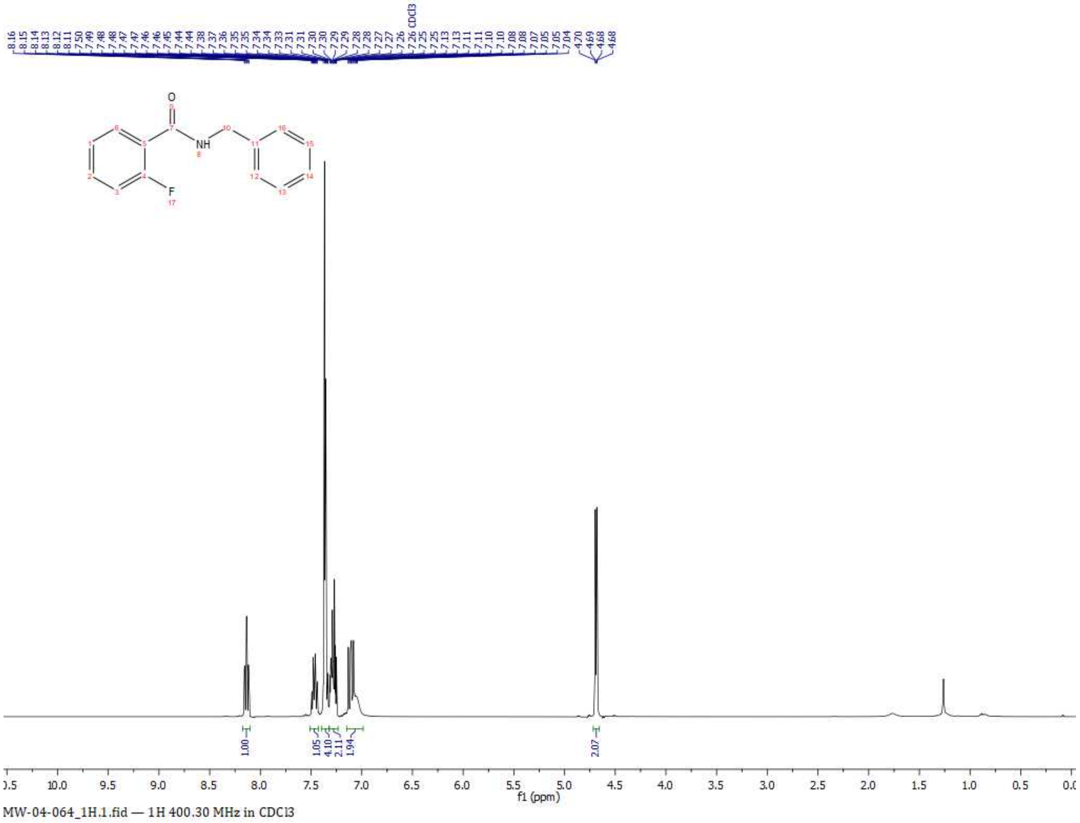

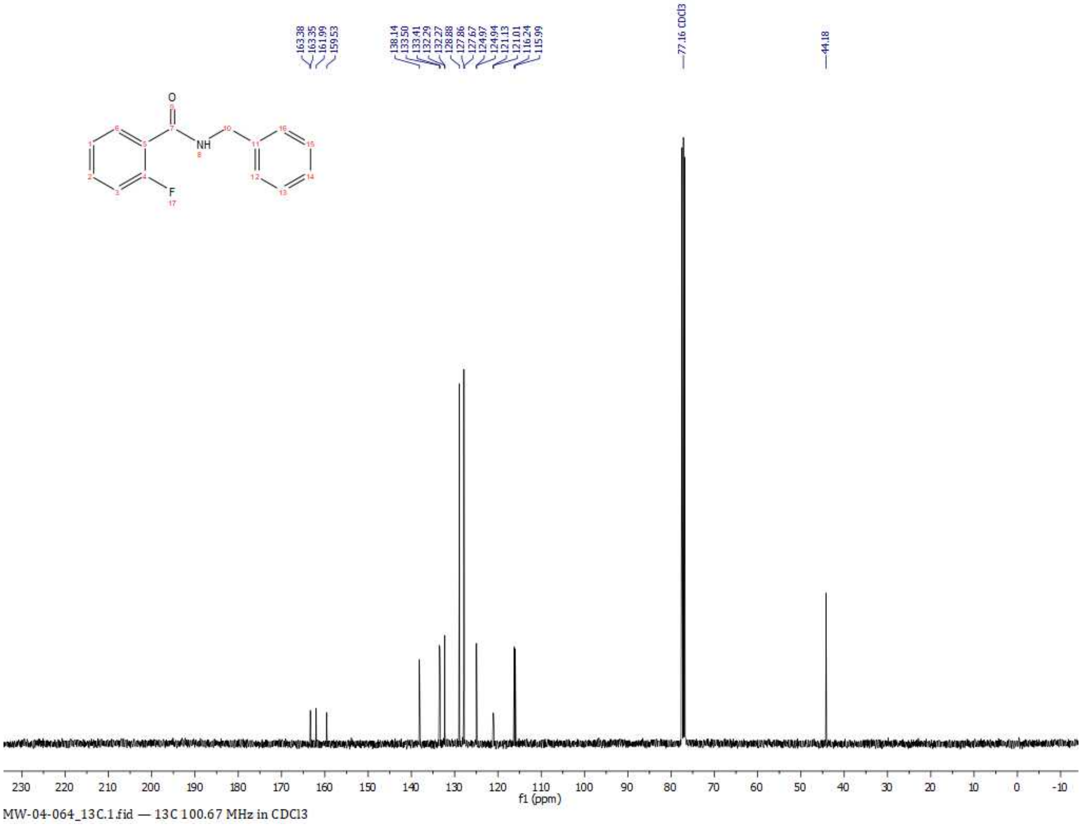

^{1}H NMR (500.3 MHz) and ^{13}C NMR (100.67) for Compound **16** in $CDCl_3$:

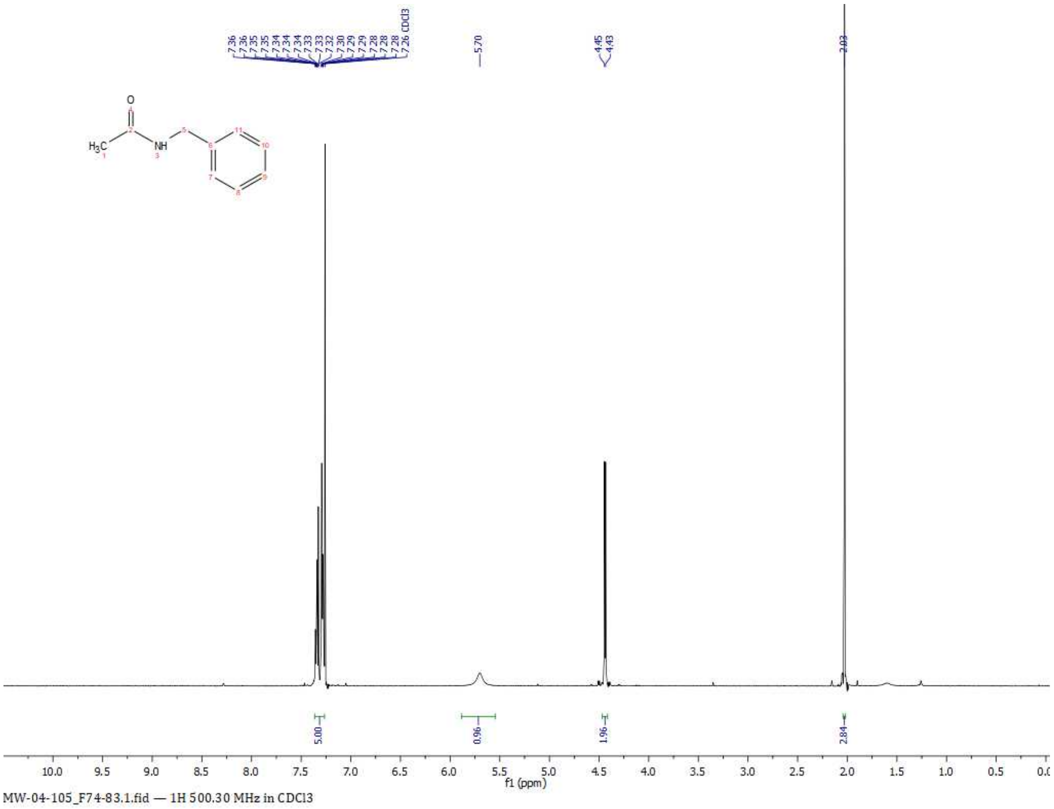

MW-04-105_F74-83.1.fid — 1H 500.30 MHz in CDCl3

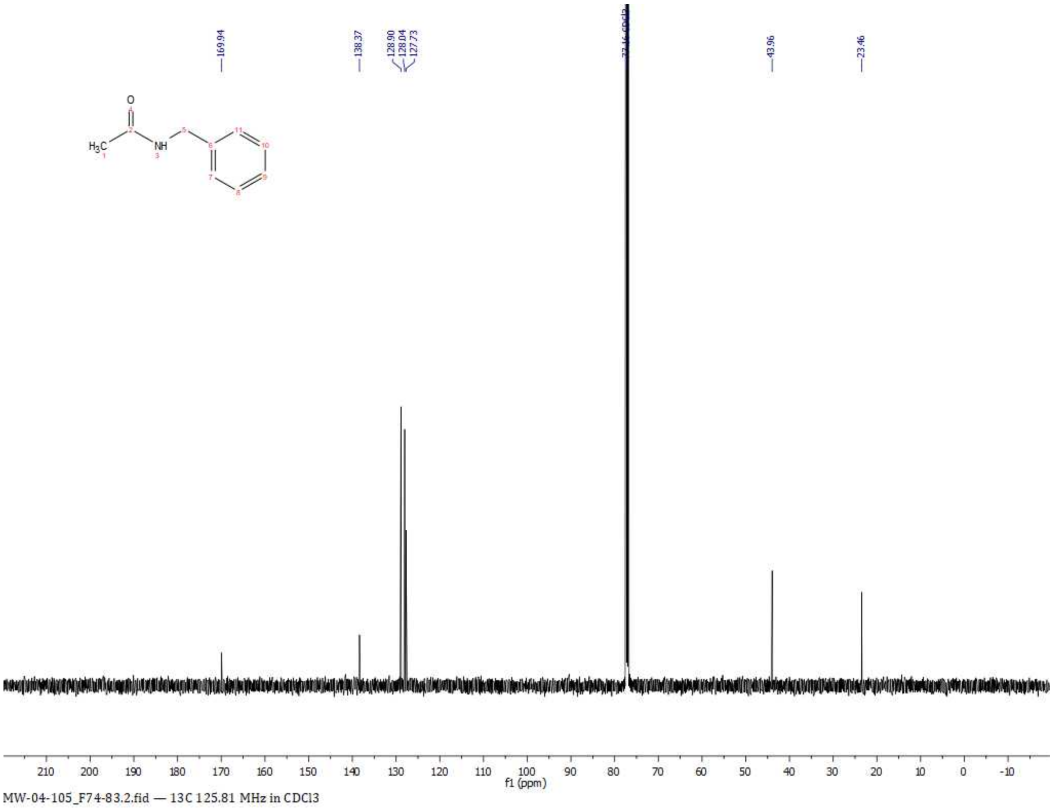

MW-04-105_F74-83.2.fid — 13C 125.81 MHz in CDCl3

^{1}H NMR (400.15 MHz) and ^{13}C NMR (100.67) for Compound **17** in $CDCl_3$:

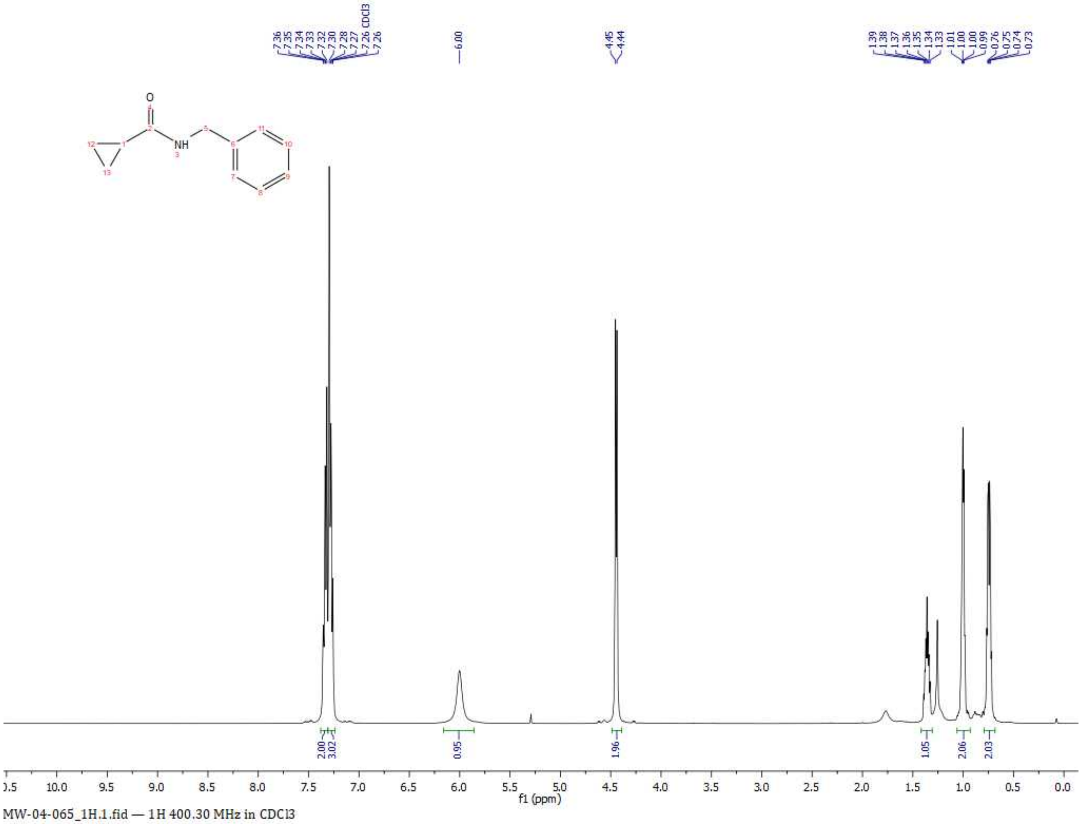

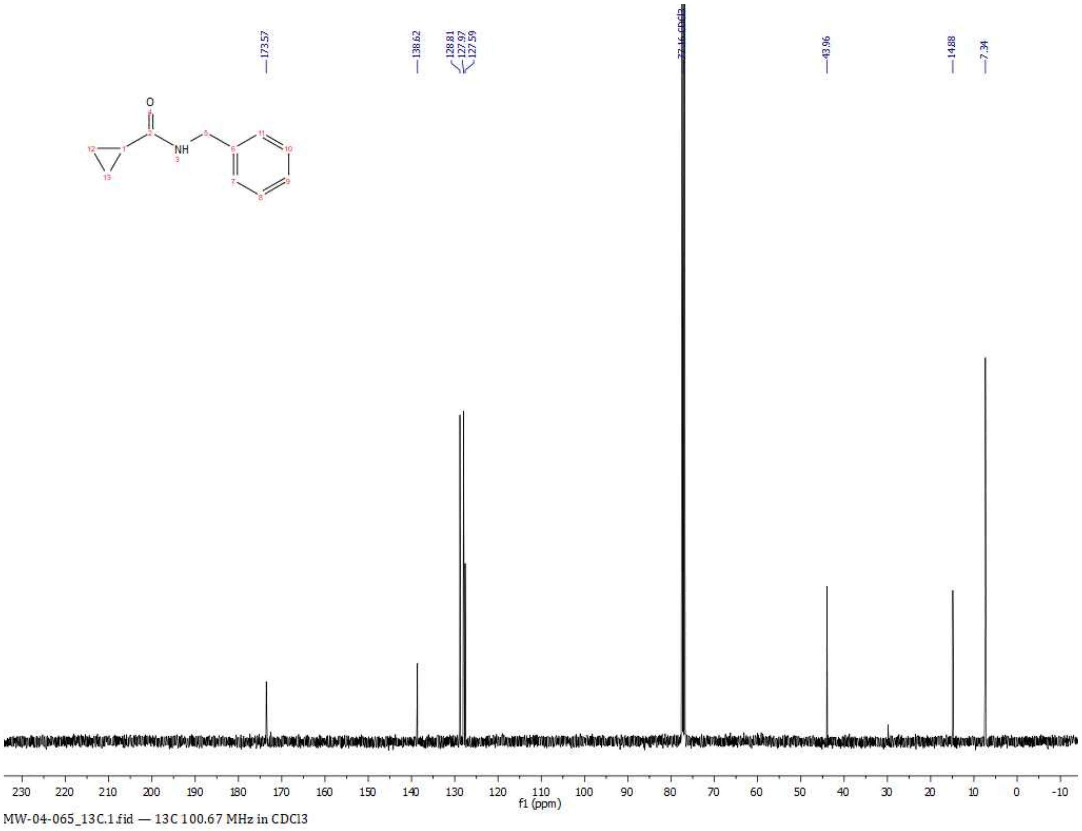

^{1}H NMR (400.15 MHz) and ^{13}C NMR (100.67) for Compound **18** in $CDCl_3$:

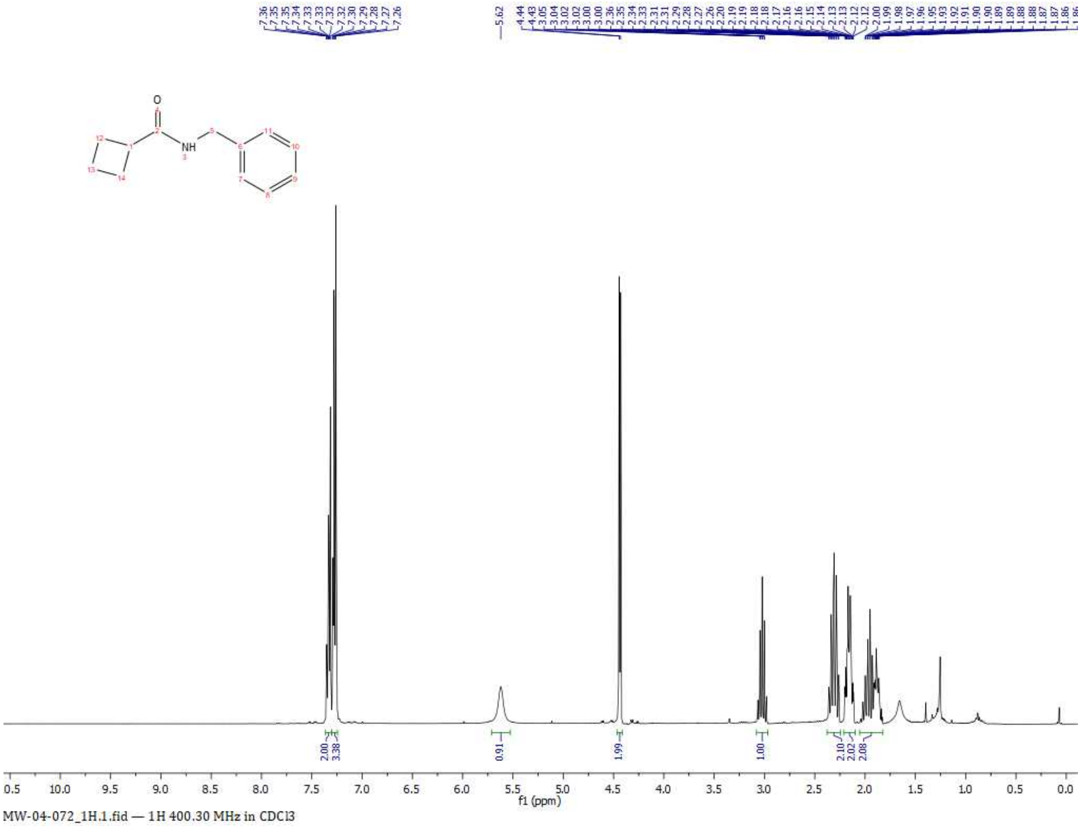

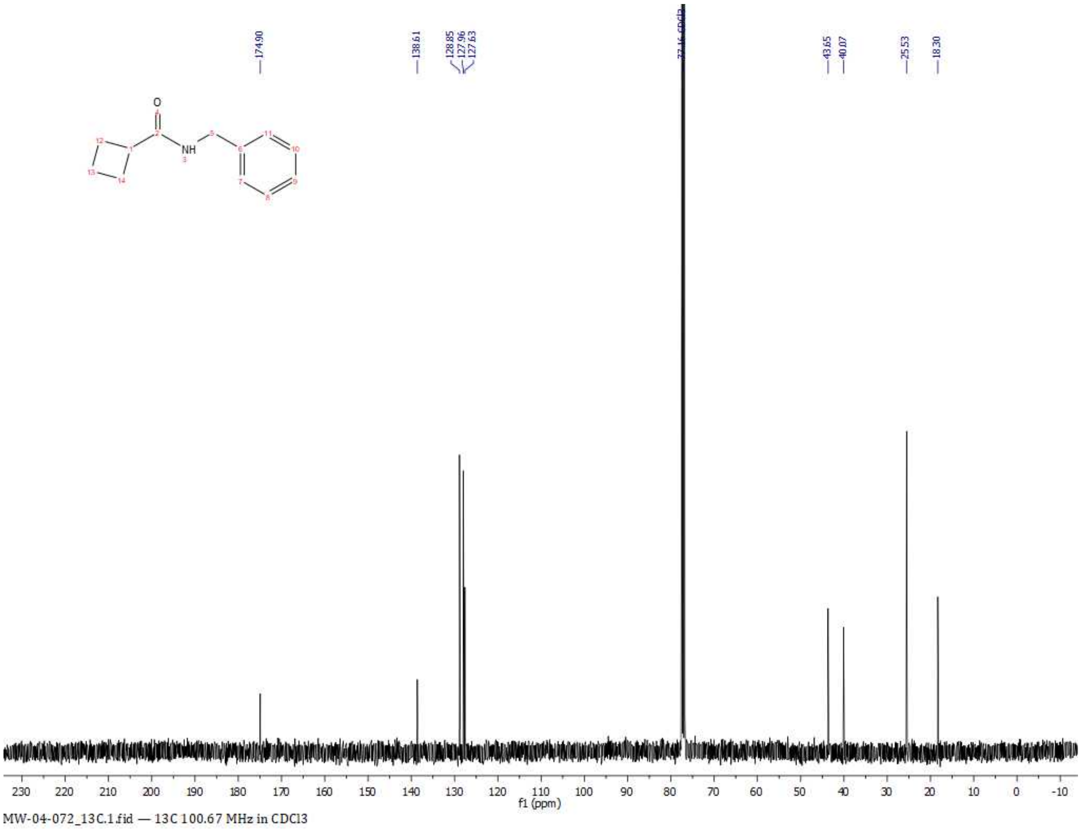

^{1}H NMR (400.15 MHz) and ^{13}C NMR (100.67) for Compound **19** in $CDCl_3$:

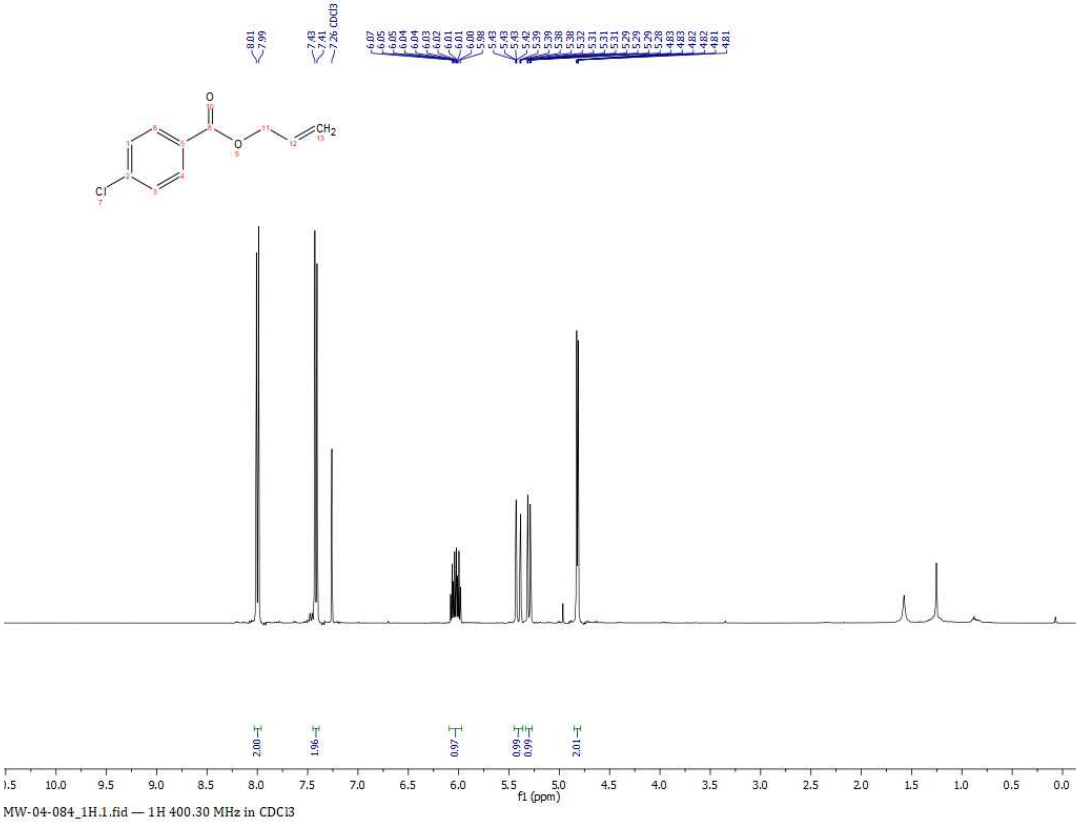

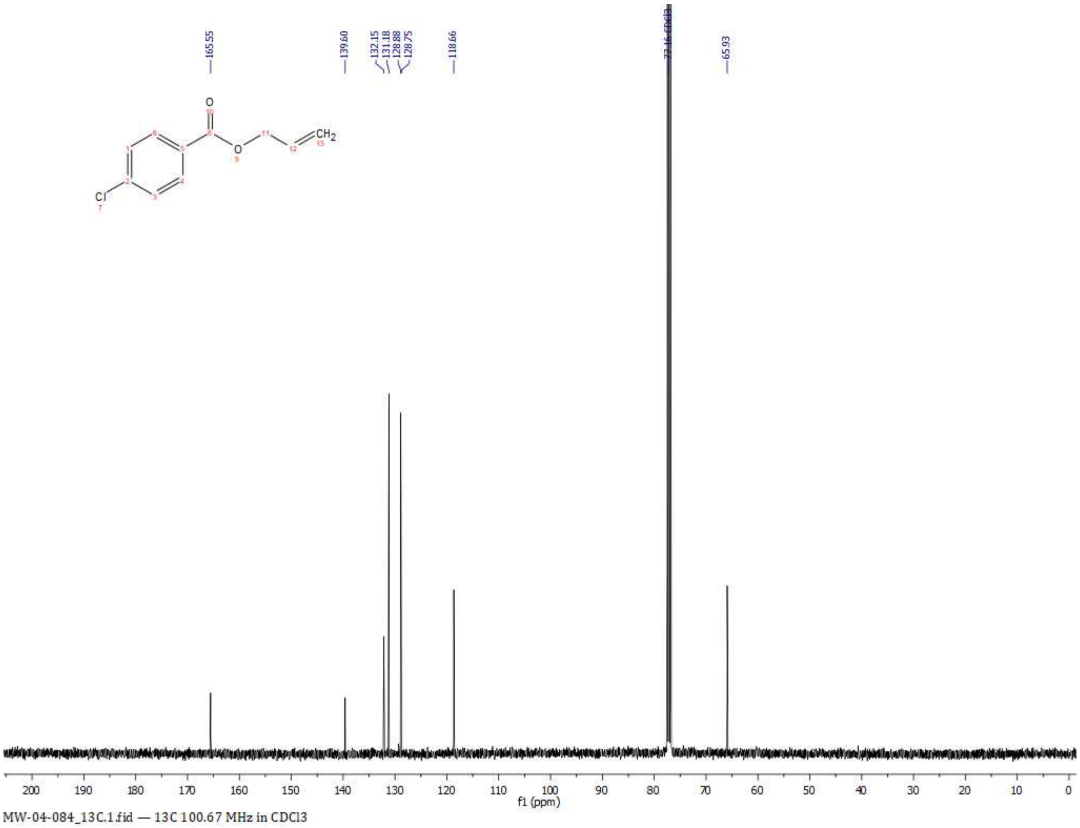

^{1}H NMR (400.15 MHz) and ^{13}C NMR (100.67) for Compound **20** in $CDCl_3$:

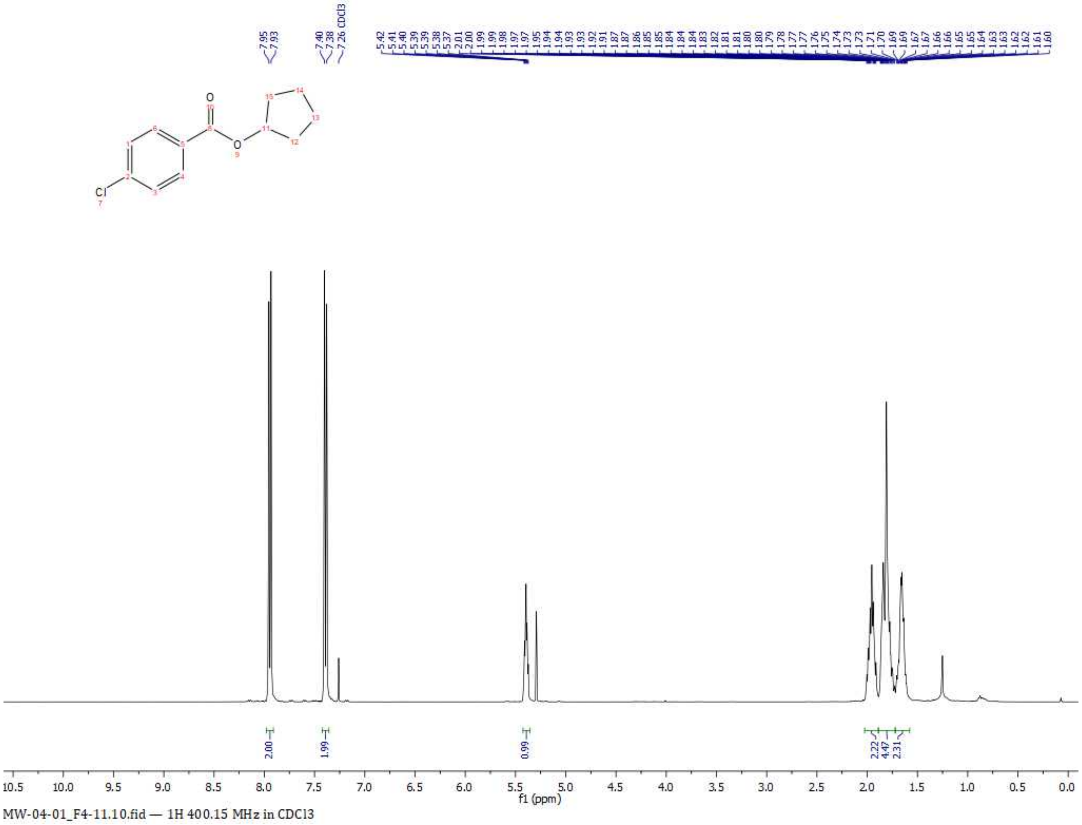

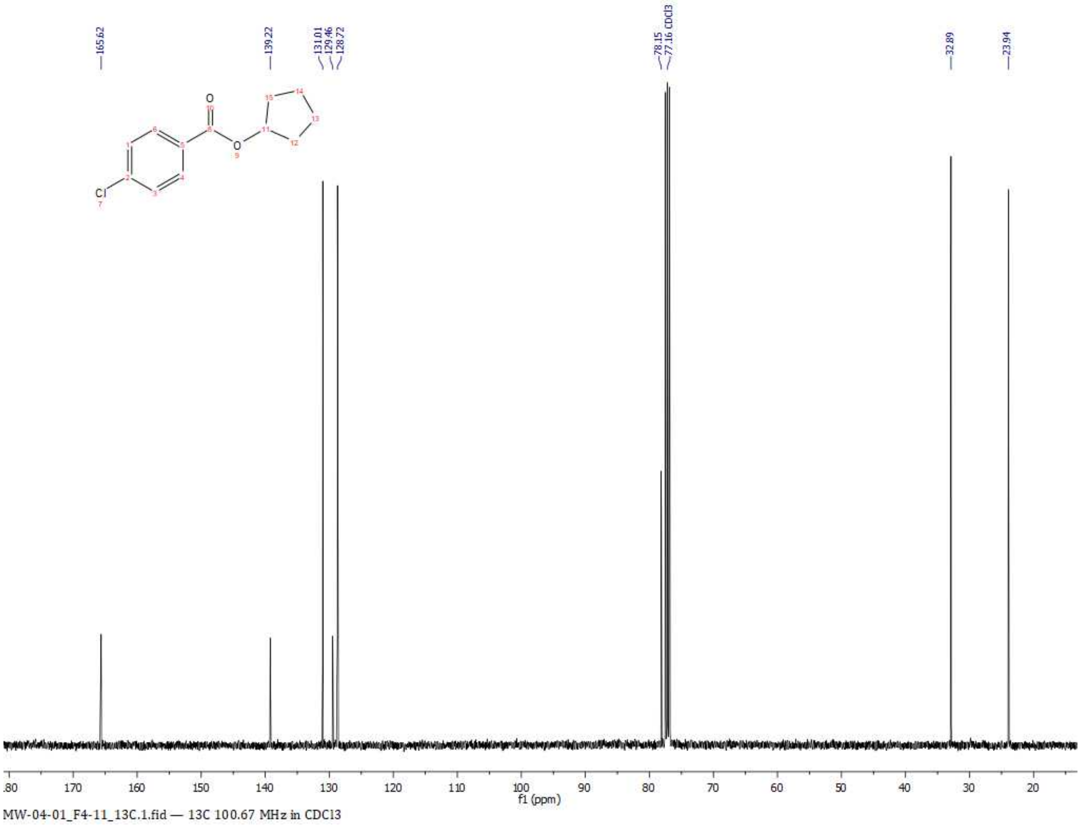

^{1}H NMR (400.15 MHz) and ^{13}C NMR (100.67) for Compound **21** in $CDCl_3$:

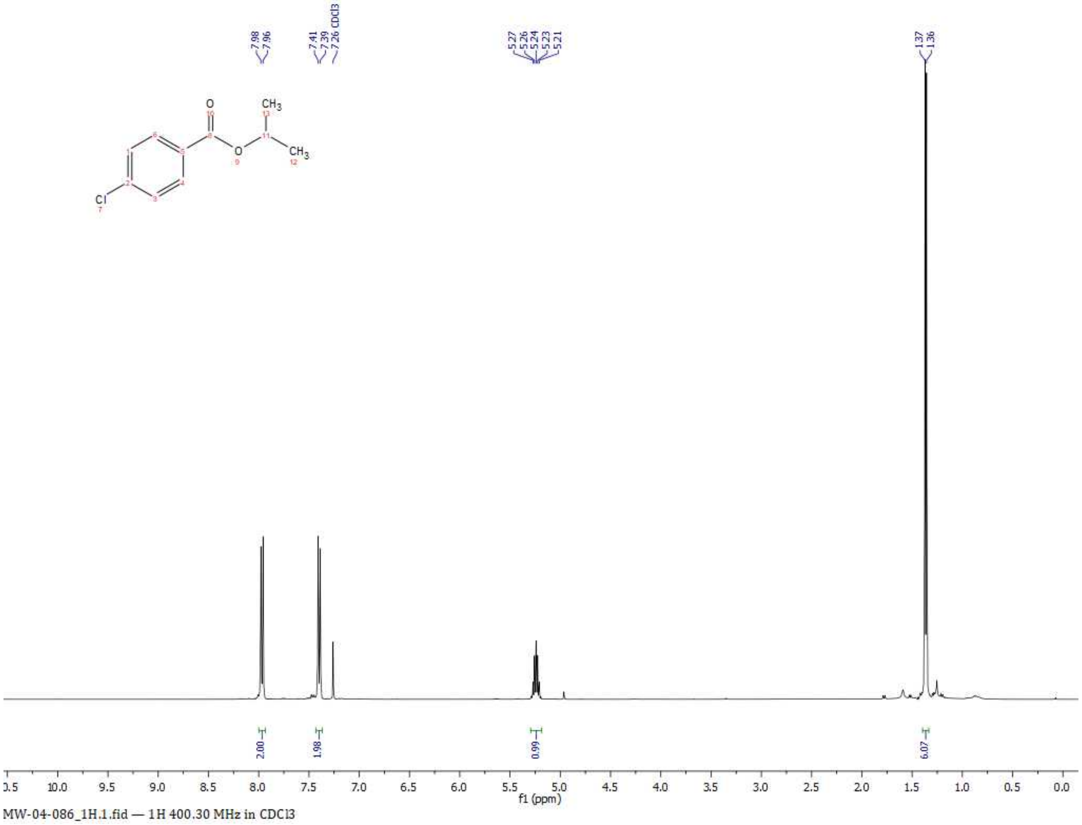

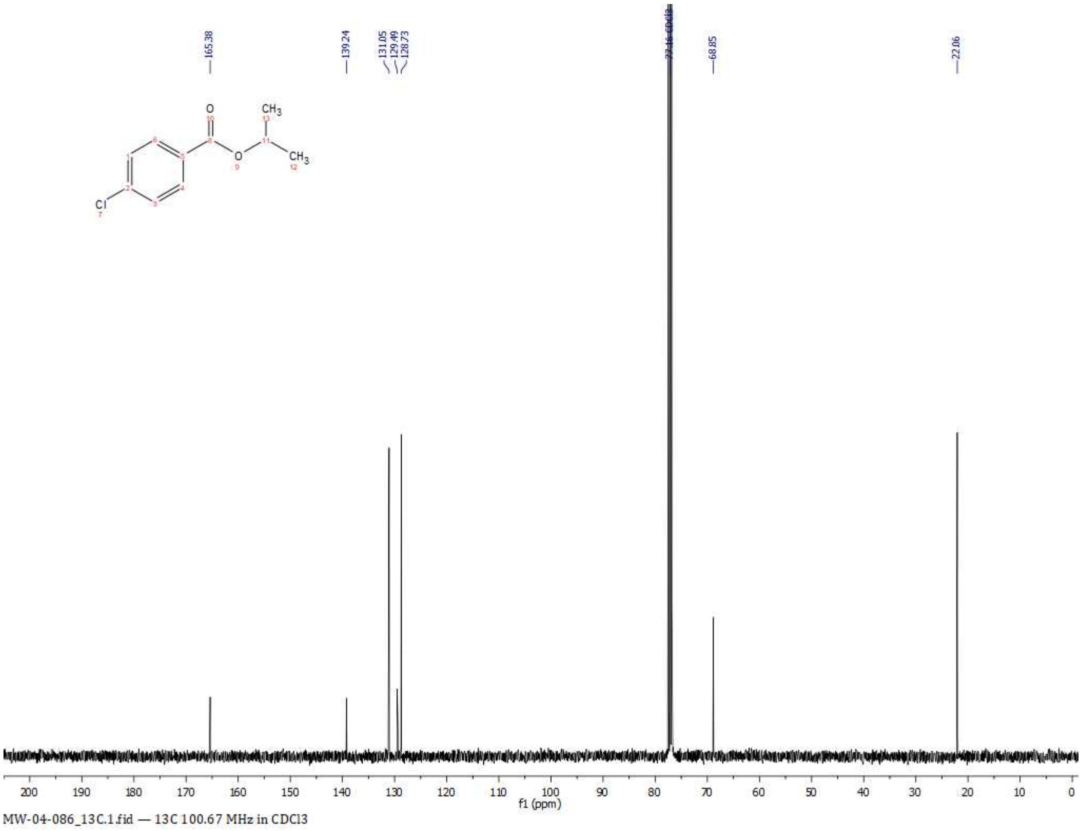

^{1}H NMR (400.15 MHz) and ^{13}C NMR (100.67) for Compound **22** in $CDCl_3$:

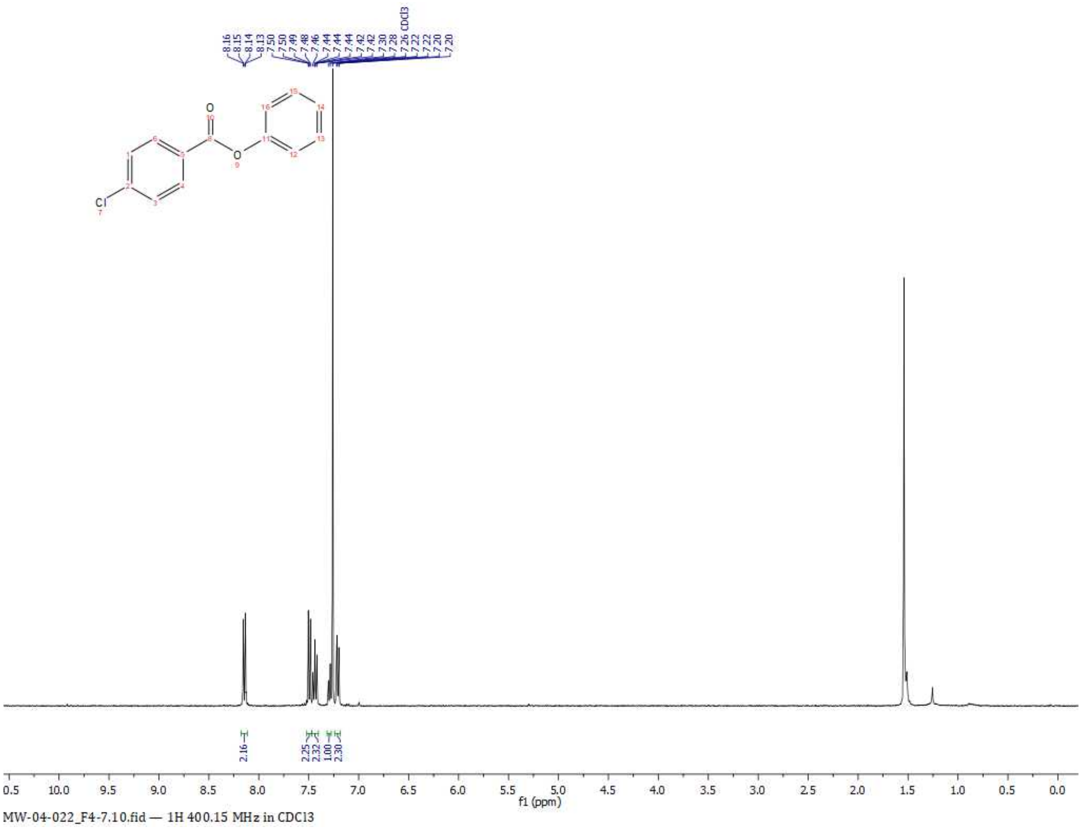

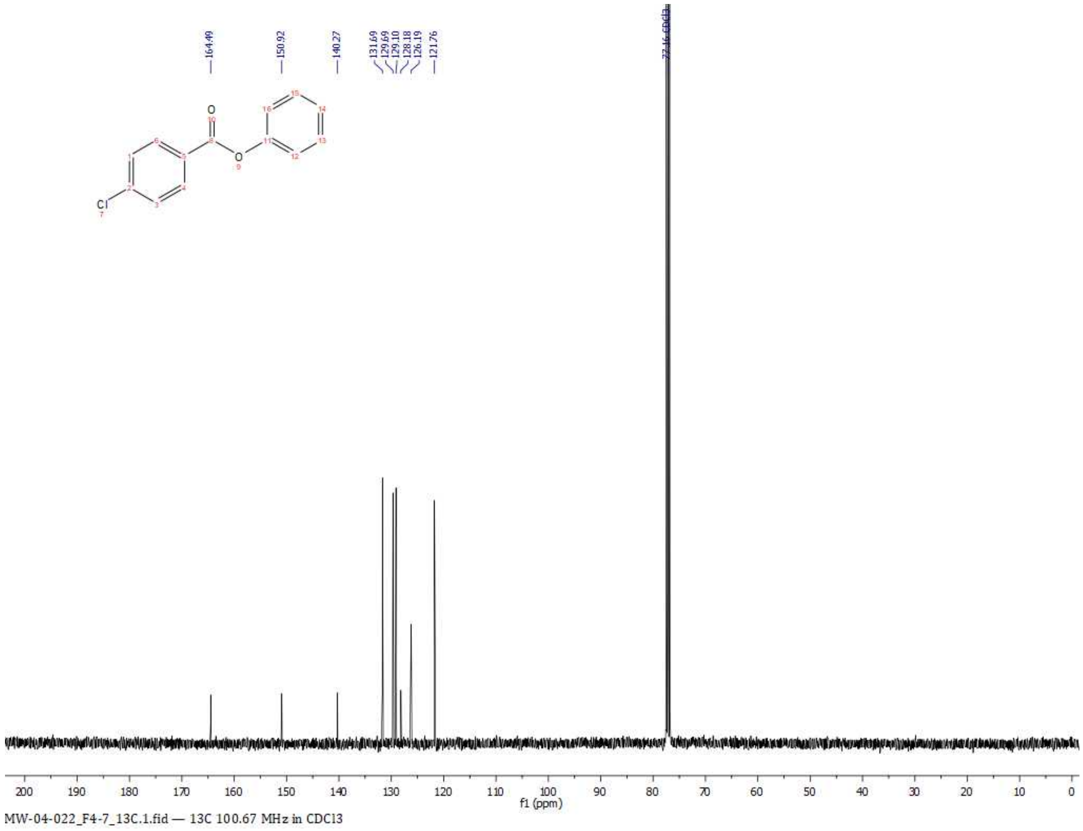

^{1}H NMR (400.15 MHz) and ^{13}C NMR (100.67) for Compound **23** in $CDCl_3$:

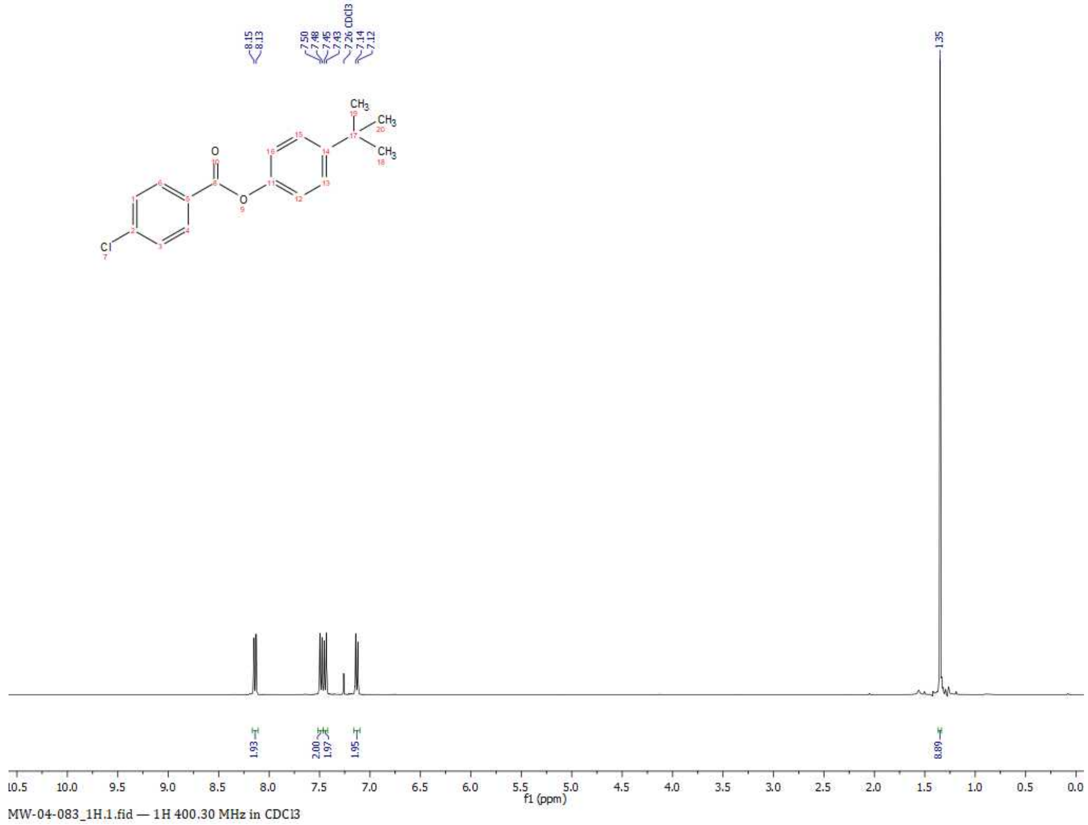

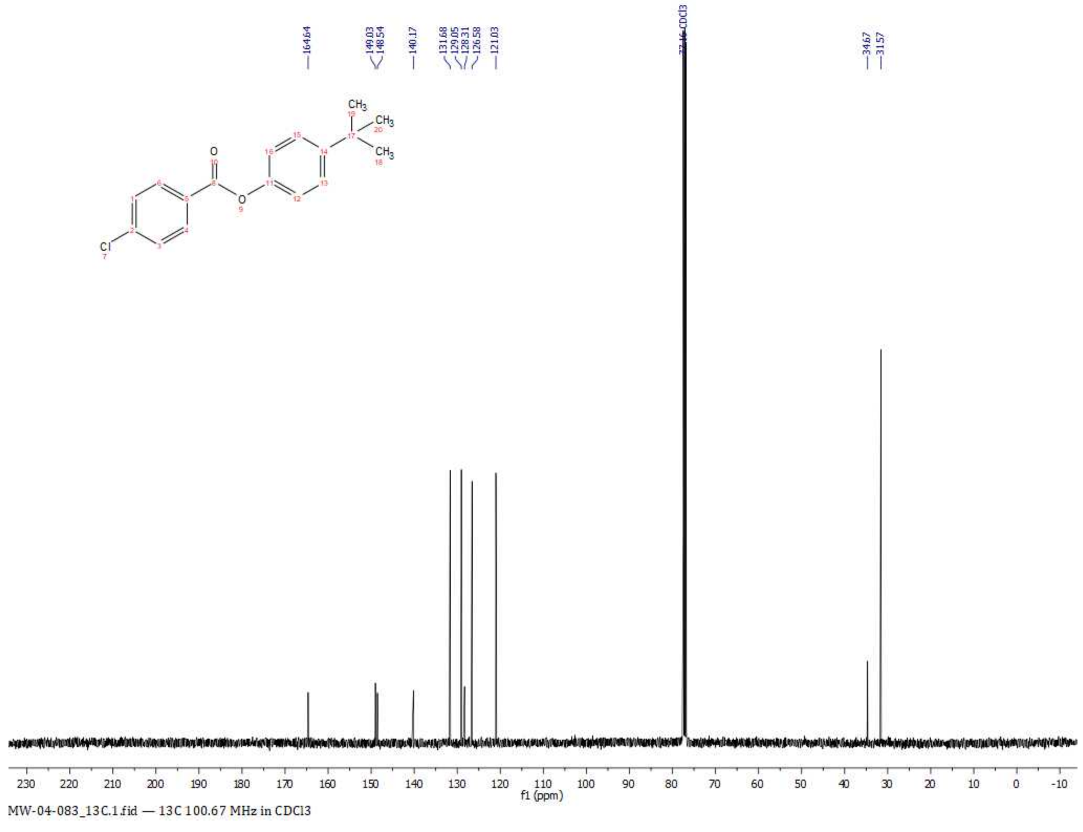

^{1}H NMR (400.15 MHz) and ^{13}C NMR (100.67) for Compound **24** in $CDCl_3$:

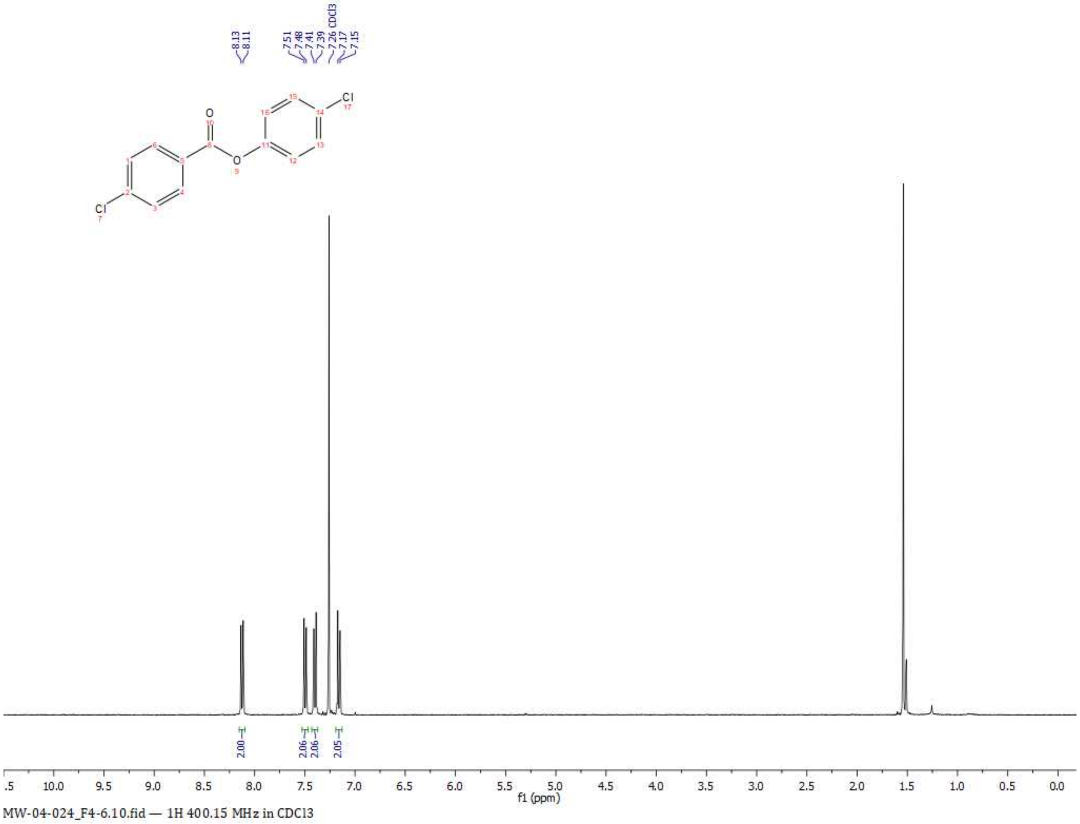

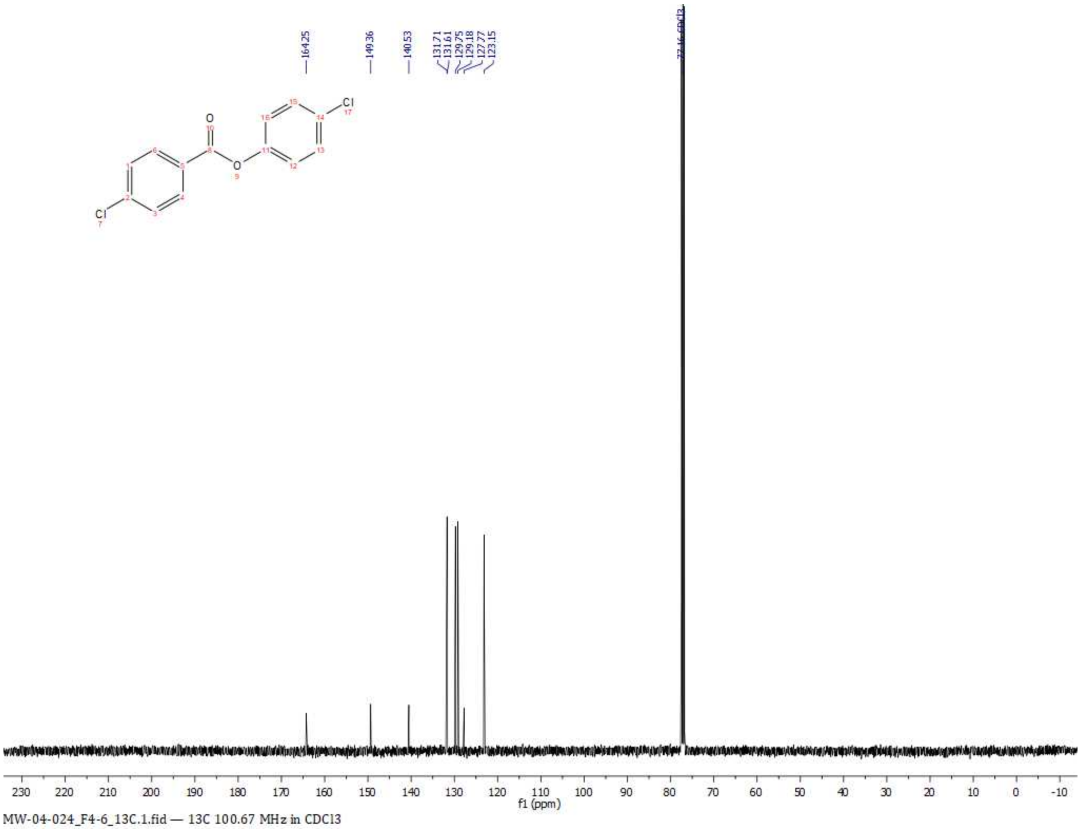

^{1}H NMR (500.3 MHz) and ^{13}C NMR (125.82 MHz) for Compound **25** in $CDCl_3$:

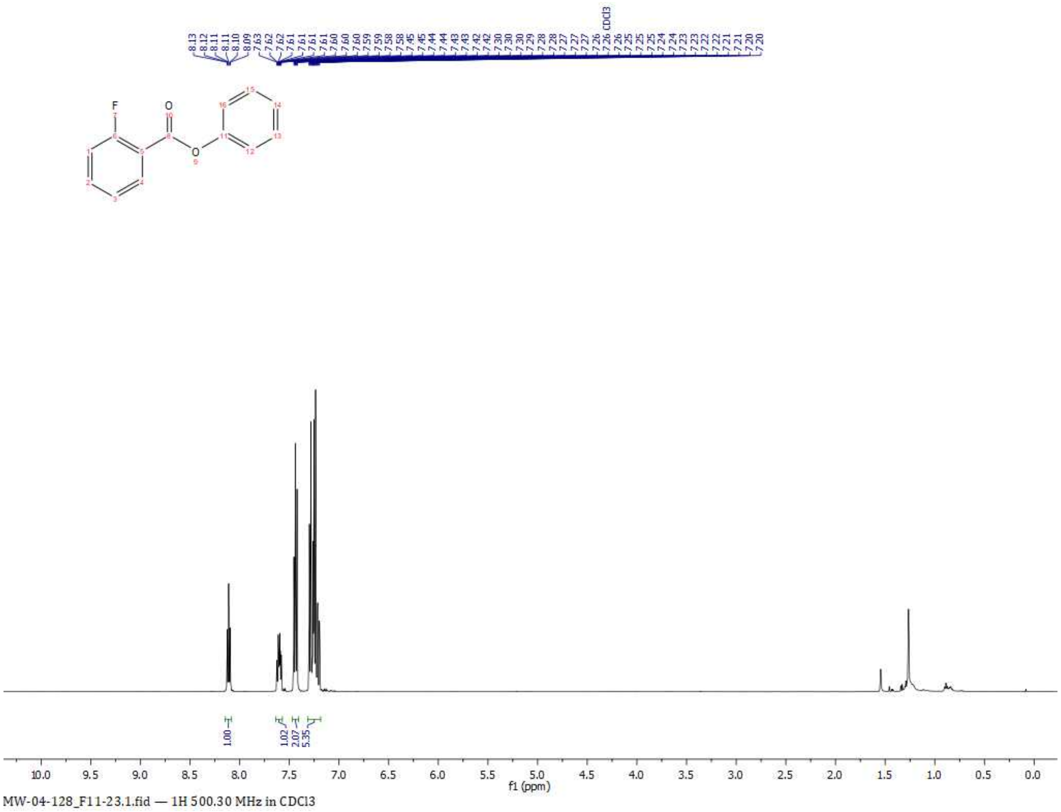

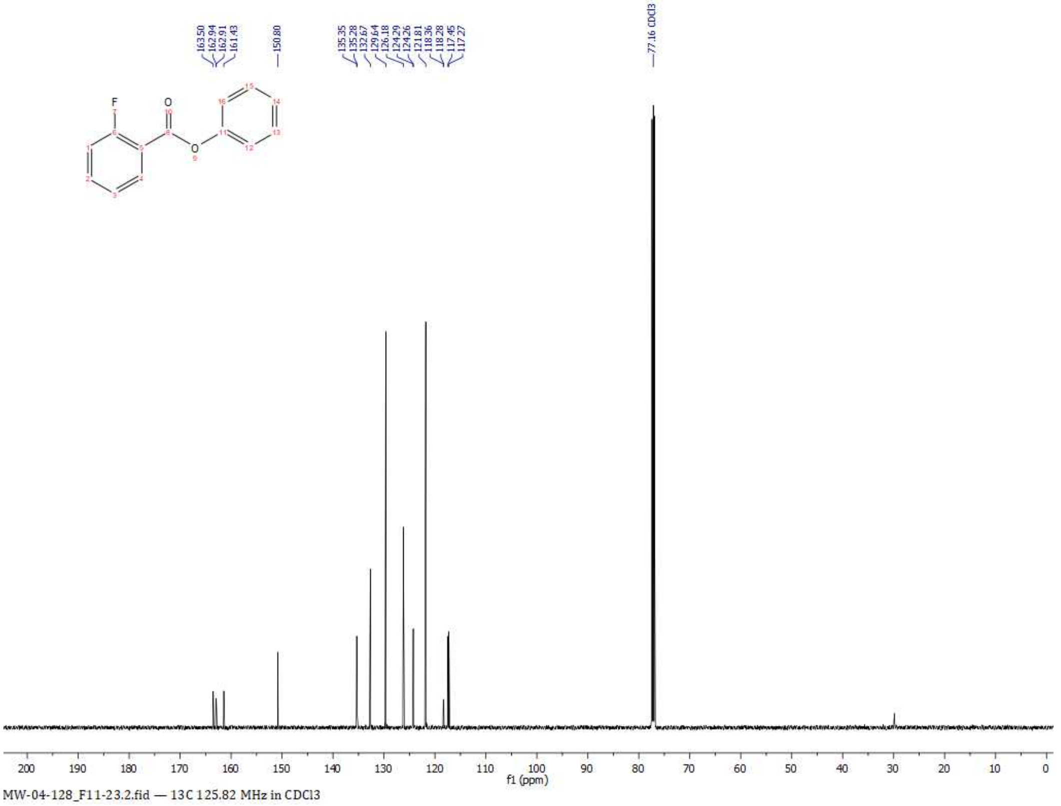

^{1}H NMR (400.15 MHz) and ^{13}C NMR (100.67) for Compound **26** in $CDCl_3$:

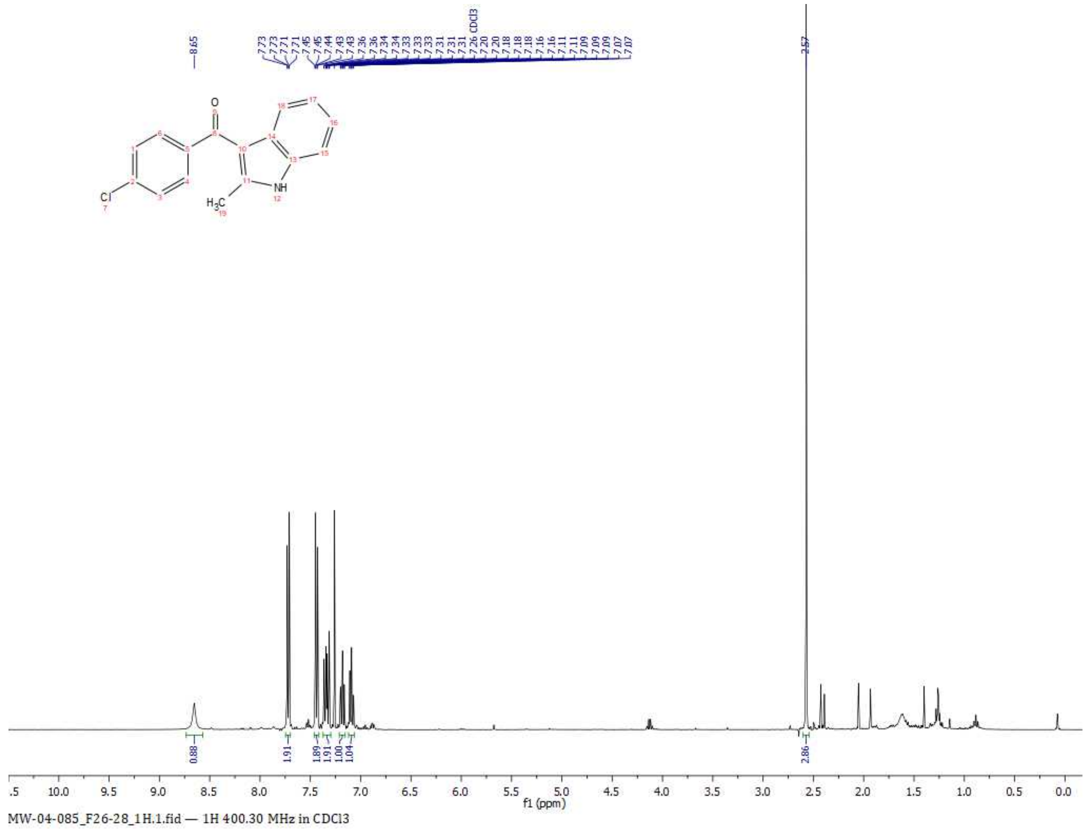

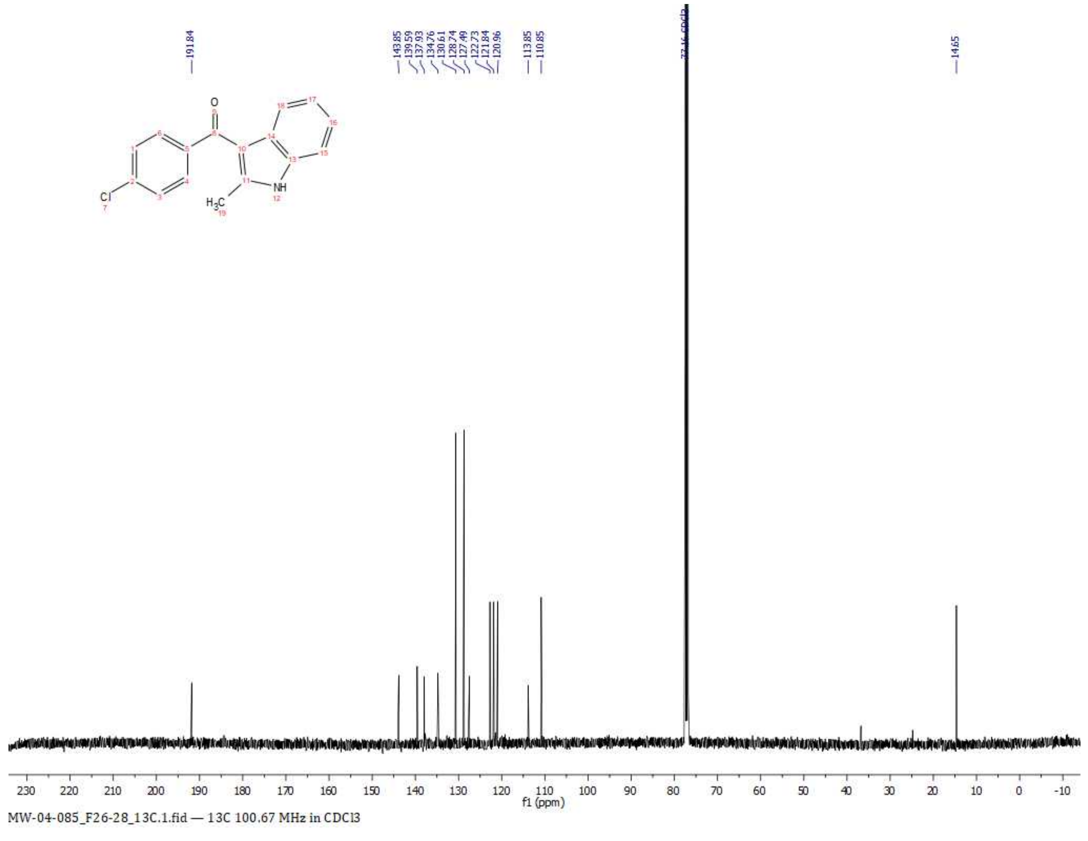

^{1}H NMR (400.15 MHz) and ^{13}C NMR (100.67) for Compound **27** in $CDCl_3$:

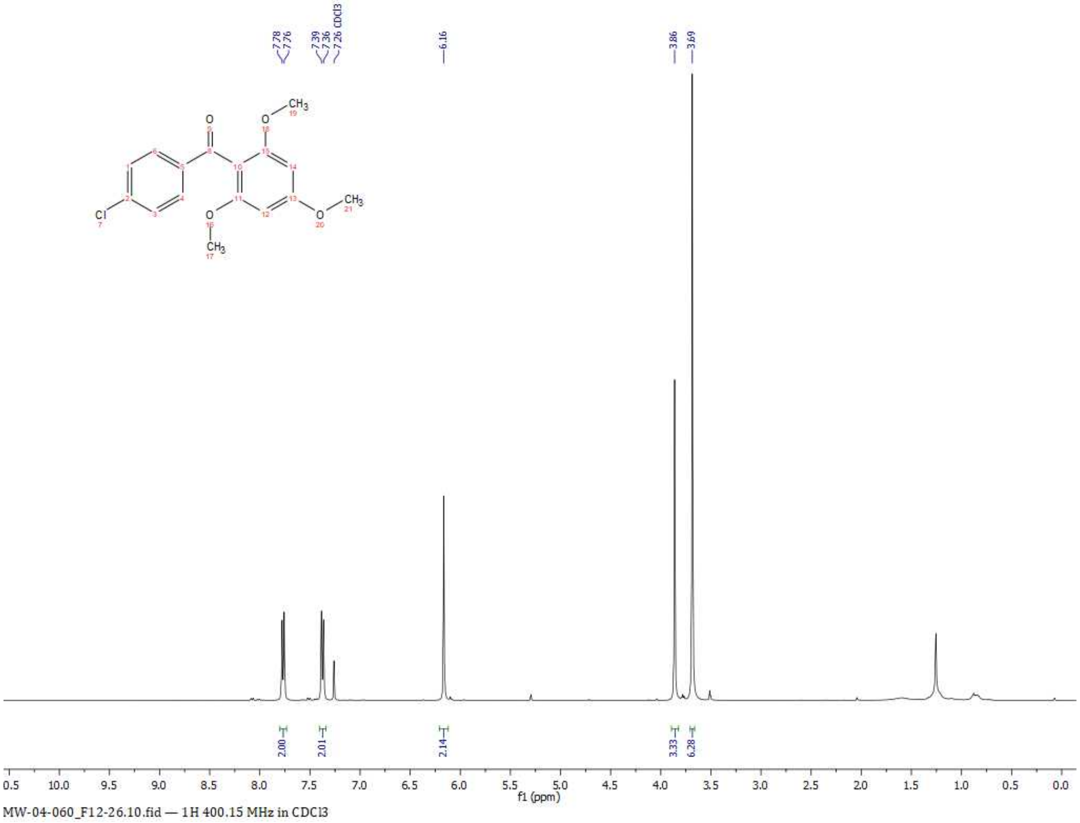

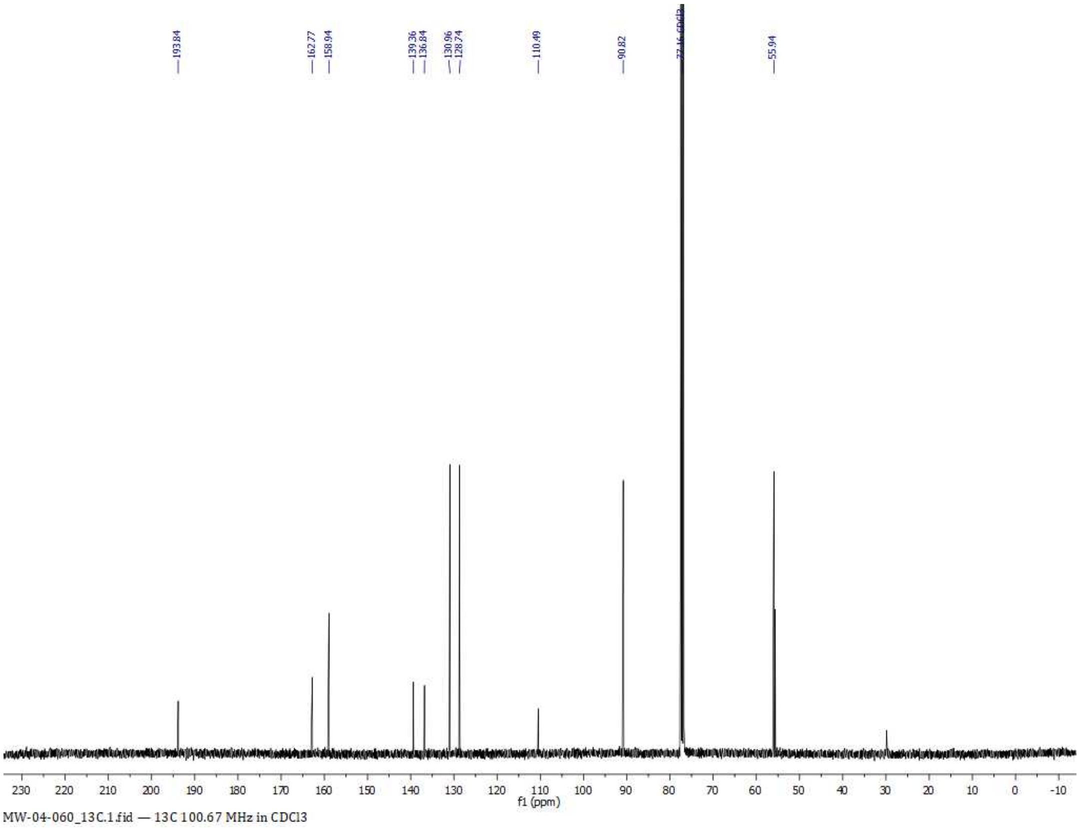

^{1}H NMR (500.3 MHz) and ^{13}C NMR (125.82 MHz) for Compound **28** in $CDCl_3$:

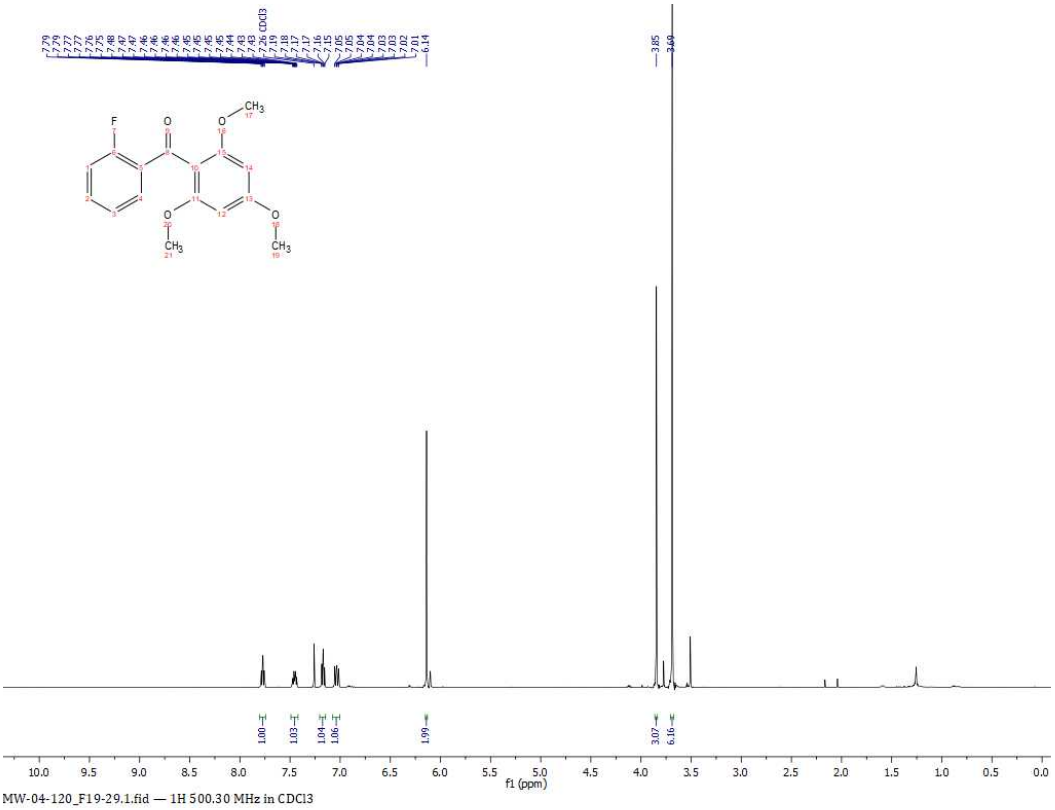

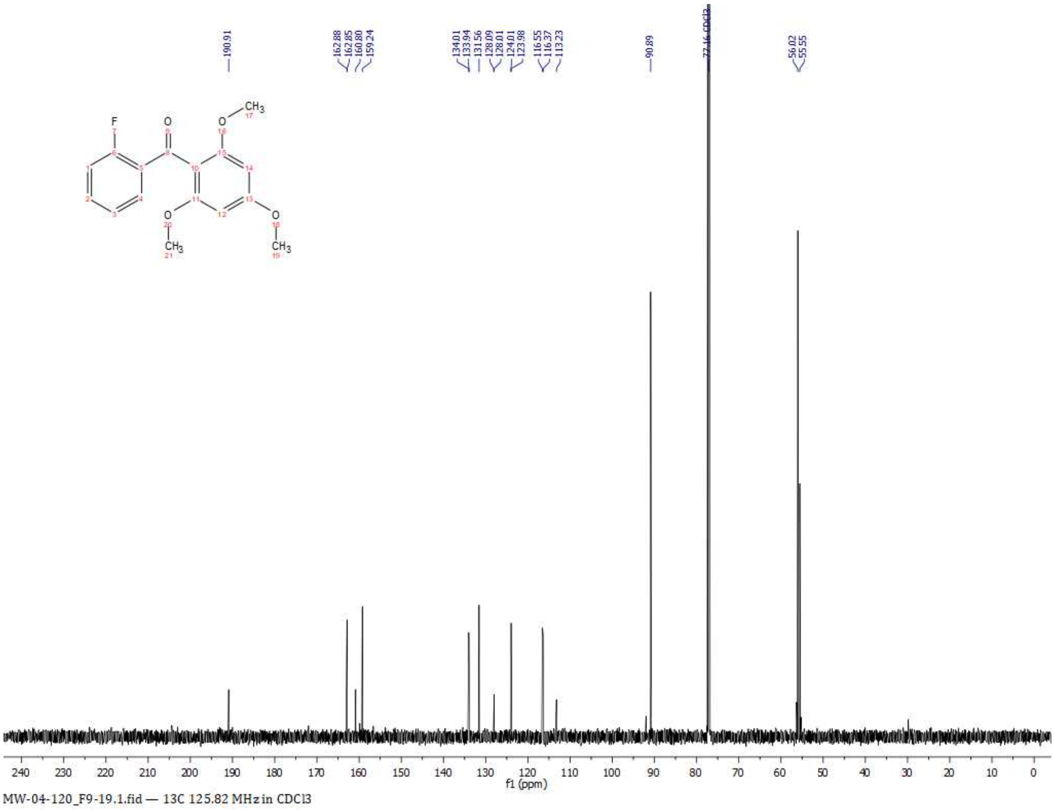

^{1}H NMR (400.15 MHz) and ^{13}C NMR (100.67) for Compound **31** in $CDCl_3$:

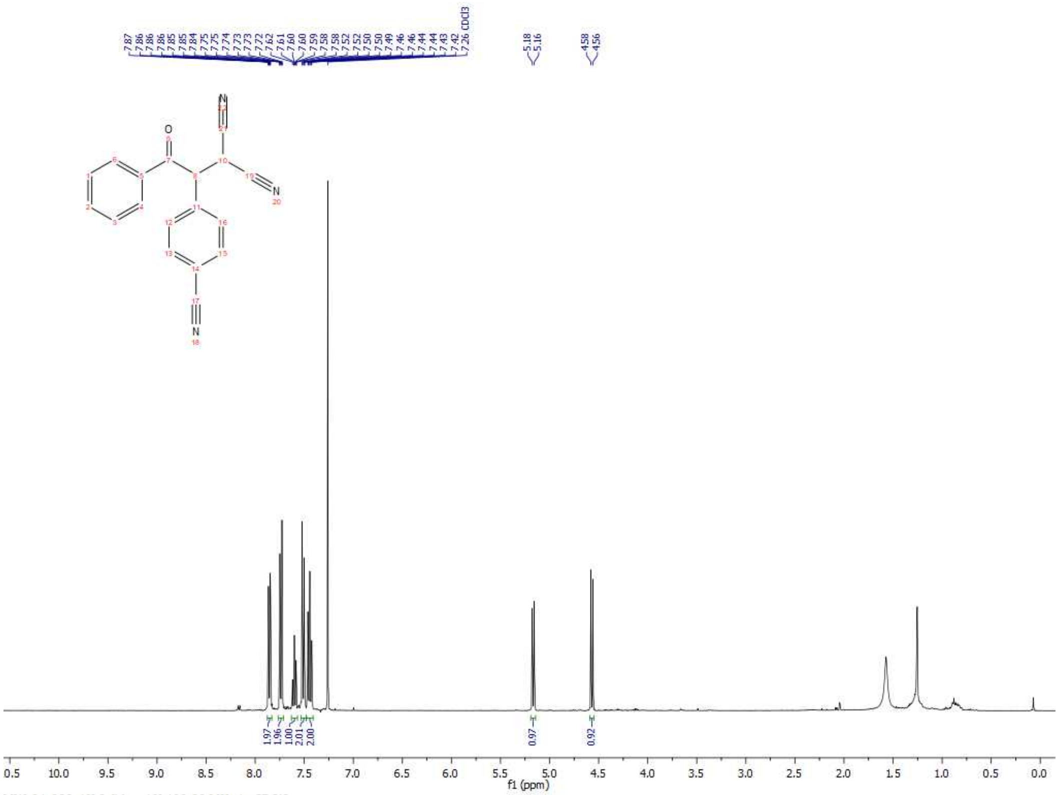

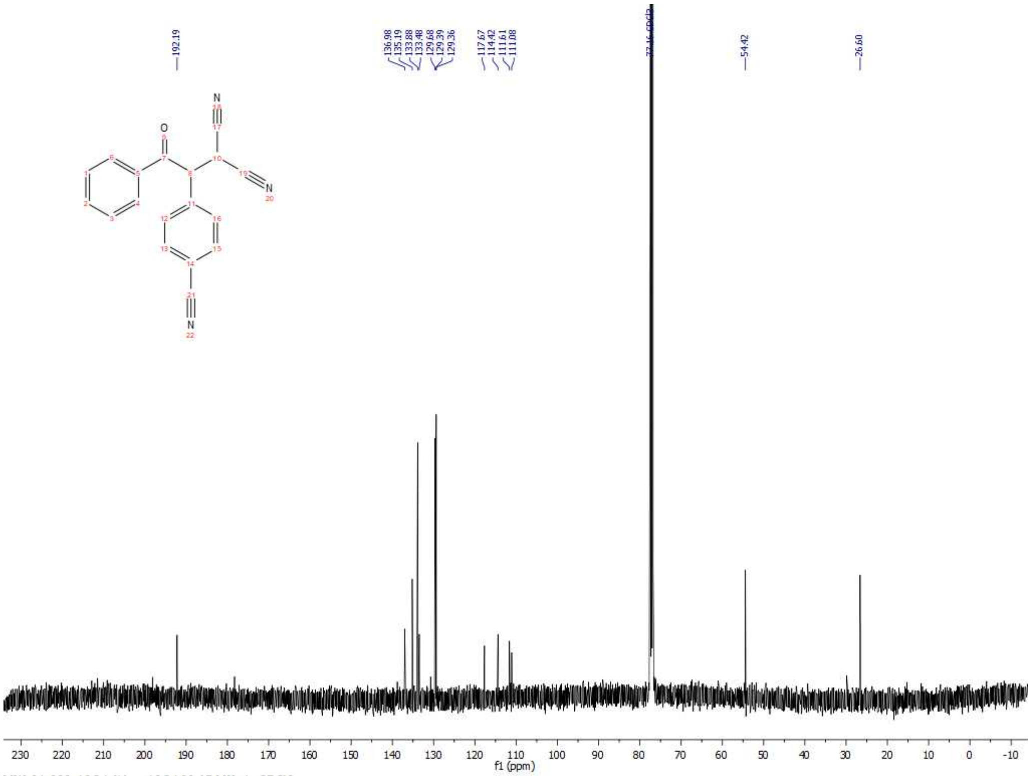

^{1}H NMR (400.15 MHz) and ^{13}C NMR (100.67) for Compound **S1** in $CDCl_3$:

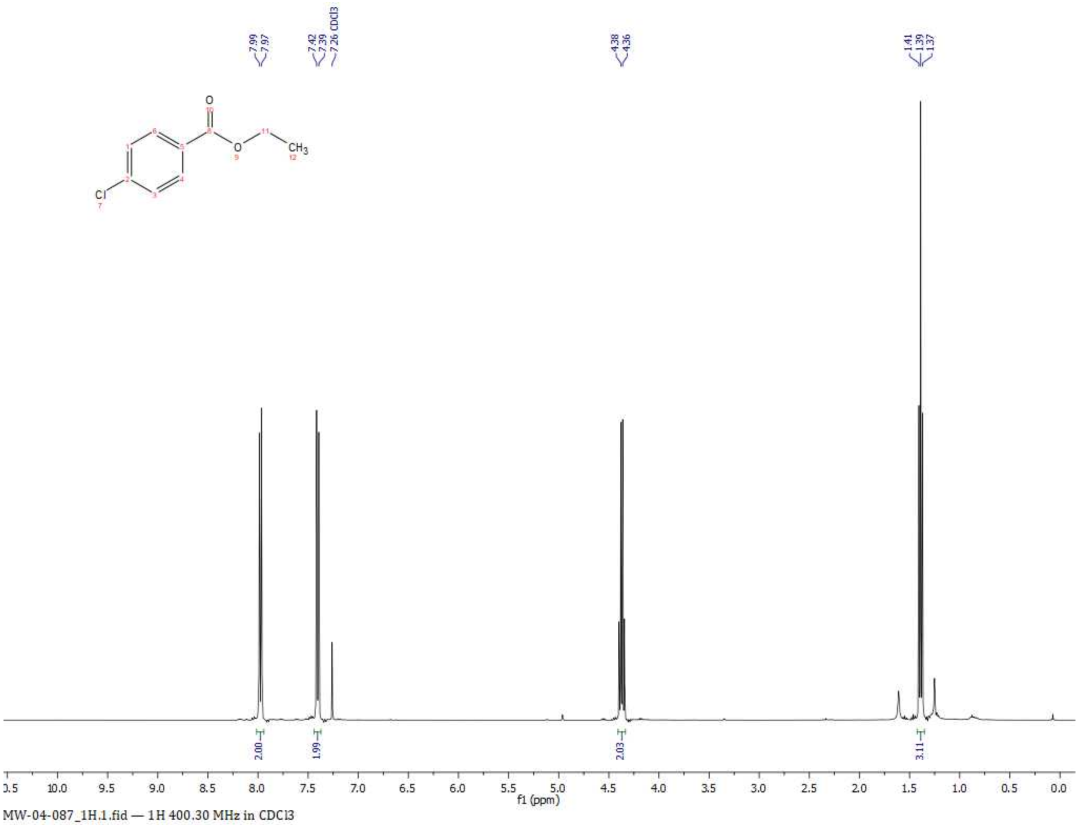

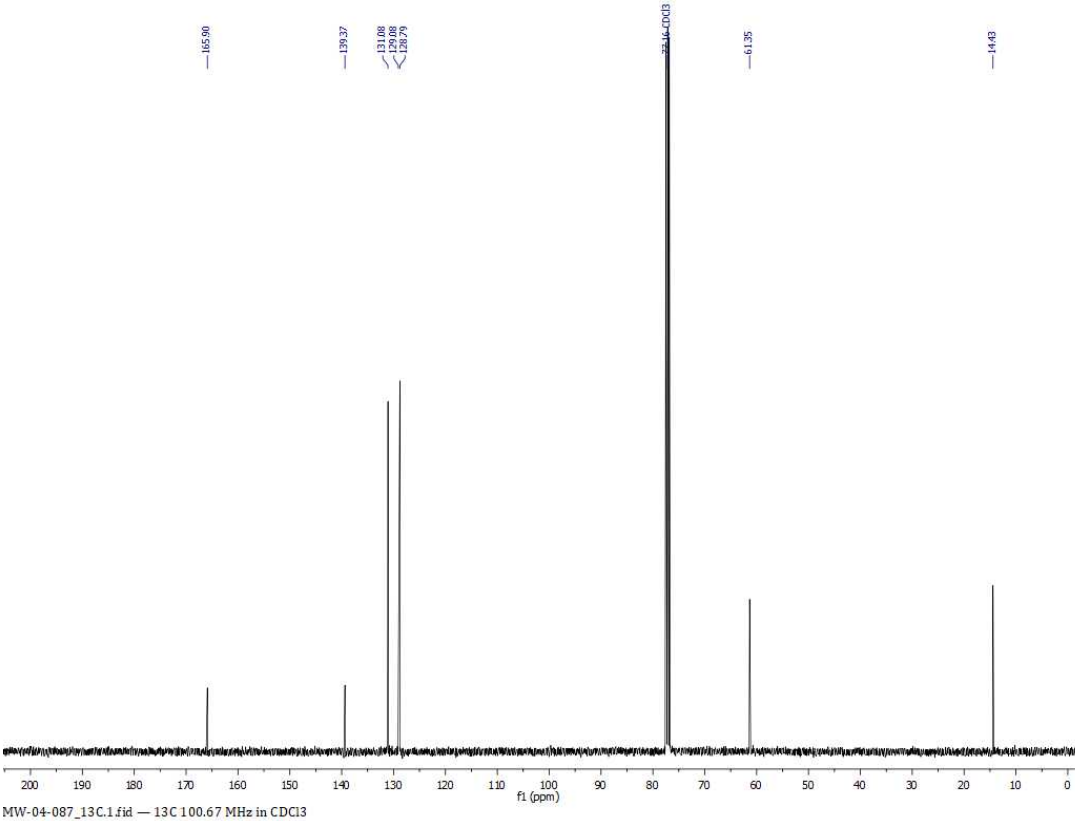

Supplement: Supplementary file 1 [file ol5c04928_si_001.pdf]
